# Optimization of 2,8-Diaryl-1,5-naphthyridines as *Plasmodium falciparum* Phosphatidylinositol 4‑Kinase Inhibitors with Improved ADME Profiles and In Vivo Efficacy

**DOI:** 10.1021/acs.jmedchem.5c02248

**Published:** 2025-10-07

**Authors:** Godwin A. Dziwornu, Donald Seanego, Stephen Fienberg, Venkata S. Sypu, Nicolaas Salomane, Liezl Krugmann, Dale Taylor, Keabetswe Masike, Mathew Njoroge, Nonlawat Boonyalai, Marcus C. S. Lee, Luiz C. Godoy, Charisse Flerida Pasaje, Jacquin C. Niles, Gregory S. Basarab, Lauren B. Coulson, Sandeep R. Ghorpade, Kelly Chibale

**Affiliations:** † Holistic Drug Discovery and Development (H3D) Centre, Department of Chemistry, 37716 University of Cape Town, Rondebosch 7701, South Africa; ‡ Holistic Drug Discovery and Development (H3D) Centre, Institute of Infectious Disease and Molecular Medicine, 37716 University of Cape Town, Observatory, Cape Town 7925, South Africa; § Biological Chemistry and Drug Discovery, Wellcome Centre for Anti-Infectives Research, 3042 University of Dundee, Dundee DD1 5EH, U.K.; ∥ Department of Biological Engineering, 2167 Massachusetts Institute of Technology, Cambridge, Massachusetts 02139, United States; ⊥ South African Medical Research Council Drug Discovery and Development Research Unit, Department of Chemistry and Institute of Infectious Disease and Molecular Medicine, 37716 University of Cape Town, Rondebosch 7701, South Africa

## Abstract

Previously reported antimalarial *Plasmodium* phosphatidylinositol 4-kinase IIIβ 2,8-diaryl-1,5-naphthyridine inhibitors have shown suboptimal physicochemical and pharmacokinetic properties. A focused target-based structure–activity relationship and structure–property optimization studies identified several compounds with good target and whole-cell activities and improved physicochemical properties. A new frontrunner compound **27** showed an improved pharmacokinetic profile and reduced parasitaemia (91% at 4 × 50 mg/kg QD doses) in the humanized NOD-*scid IL-2Rγnull* mouse model of *Plasmodium falciparum* malaria. Compound **27** poses no hERG channel inhibition at high concentrations or mammalian cytotoxicity but shows low selectivity against related human lipid kinases (PI3Kα and PI4Kβ); however, significantly higher selectivity margins were observed against the human MINK1 and MAP4K4 kinases.

## Graphical abstract

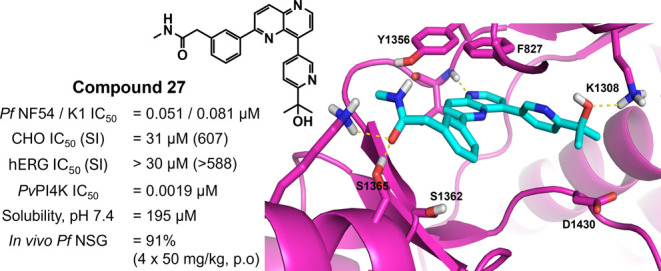

## Introduction

Recent literature has expounded on the importance of exploring and exploiting *Plasmodium* kinases as potential drug targets for malaria.^1–5^ Both protein and lipid kinases have been the focus of malaria drug discovery efforts in recent years. Many of these kinases have been identified as genetically essential,^6,7^ while some have been chemically validated with small molecule inhibitors.^2^ *Plasmodium falciparum* phosphatidylinositol 4-kinase IIIβ (*Pf*PI4K) has been clinically validated with MMV390048.^8^ MMV390048 displays potency against multiple life cycle stages of the parasite, which is a consequence of the importance of the target at all stages.^9^ Other structurally distinct *Pf*PI4K inhibitors have also been reported (Figure 1).^10–13^ The development of MMV390048 as the first clinical drug targeting *Pf*PI4K was halted due to teratogenicity, manifested mainly as diaphragmatic hernias, in rats.^14^ Inhibition of the human PI4Kβ (*Hs*PI4K), MAP4K4, and MINK1 were hypothesized to be linked to these in vivo toxicity findings as the in vivo exposures of MMV390048 in rats was above the in vitro IC_50_ values for these targets.^14^

**Figure 1:**
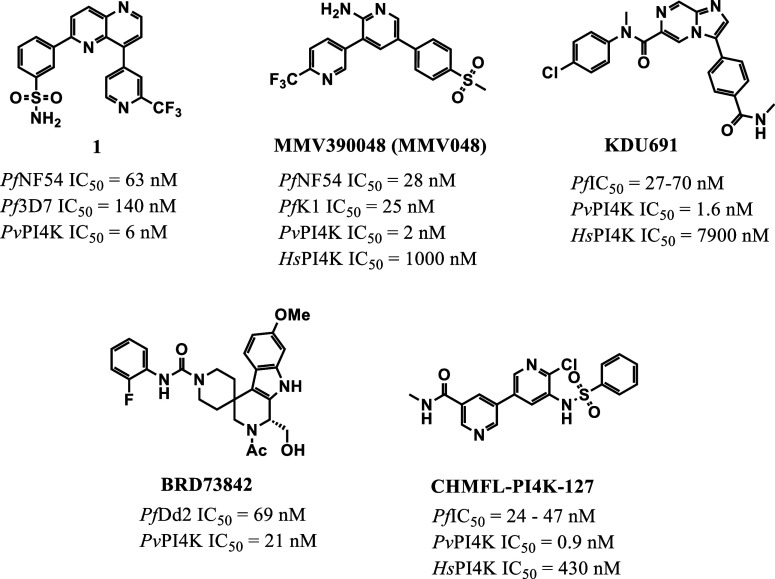
Examples of reported *Pf*PI4K inhibitors with associated in vitro asexual blood stage antiplasmodial activity and in vitro enzyme inhibition data for *P. vivax* PI4Kβ (*Pv*PI4K) and the human orthologue *Hs*PI4K.^9–13^ *Pf*IC_50_ represent activity against a panel of the drug-resistant strains of *P. falciparum*.

Previously, we reported a series of 2,8-disubstituted-1,5-naphthyridine *Pf*PI4K inhibitors, as represented by compound **1** (Figure 1).^11^ The antimalarial properties of **1** and most other compounds in the series were limited by poor physicochemical properties, especially low aqueous solubility, and poor pharmacokinetics properties - high in vivo clearance with low oral bioavailability. Here, we report structure-guided optimization of the series to improve the antiplasmodial activity and physicochemical properties. The design strategy led to compounds with high solubility and improved pharmacokinetics profiles, leading to improved in vivo efficacy, relative to **1**.^11^ The new compounds were confirmed to act through inhibition of *Pf*PI4K as the primary mode of action and were profiled for inhibition of key human kinase off-targets.

## Results and Discussion

### Chemistry

As shown in Scheme 1, compounds **11**–**32** presented herein were synthesized according to procedures previously described,^11,15^ while compound **33**–**38** were synthesized using a modified sequence. To drive the structure–activity relationship (SAR) exploration, C-8 substituents were first installed on **4** via a Suzuki cross-coupling reaction to give **5**, which on demethylation under acidic conditions afforded **6**. The C-2 hydroxyl group in **6** was tosylated with *p*-toluenesulfonyl chloride under basic conditions to give **7**. Alternatively, where the C-2 position was fixed with the C-8 group varied, demethylation of **4** to **8** preceded the tosylation of the hydroxyl group to give **9**. Regioselective Suzuki or nucleophilic aromatic substitution reaction on **9** afforded **10**. The final compounds were obtained via a Suzuki reaction on intermediate **7** or **10**.

**Scheme 1:**
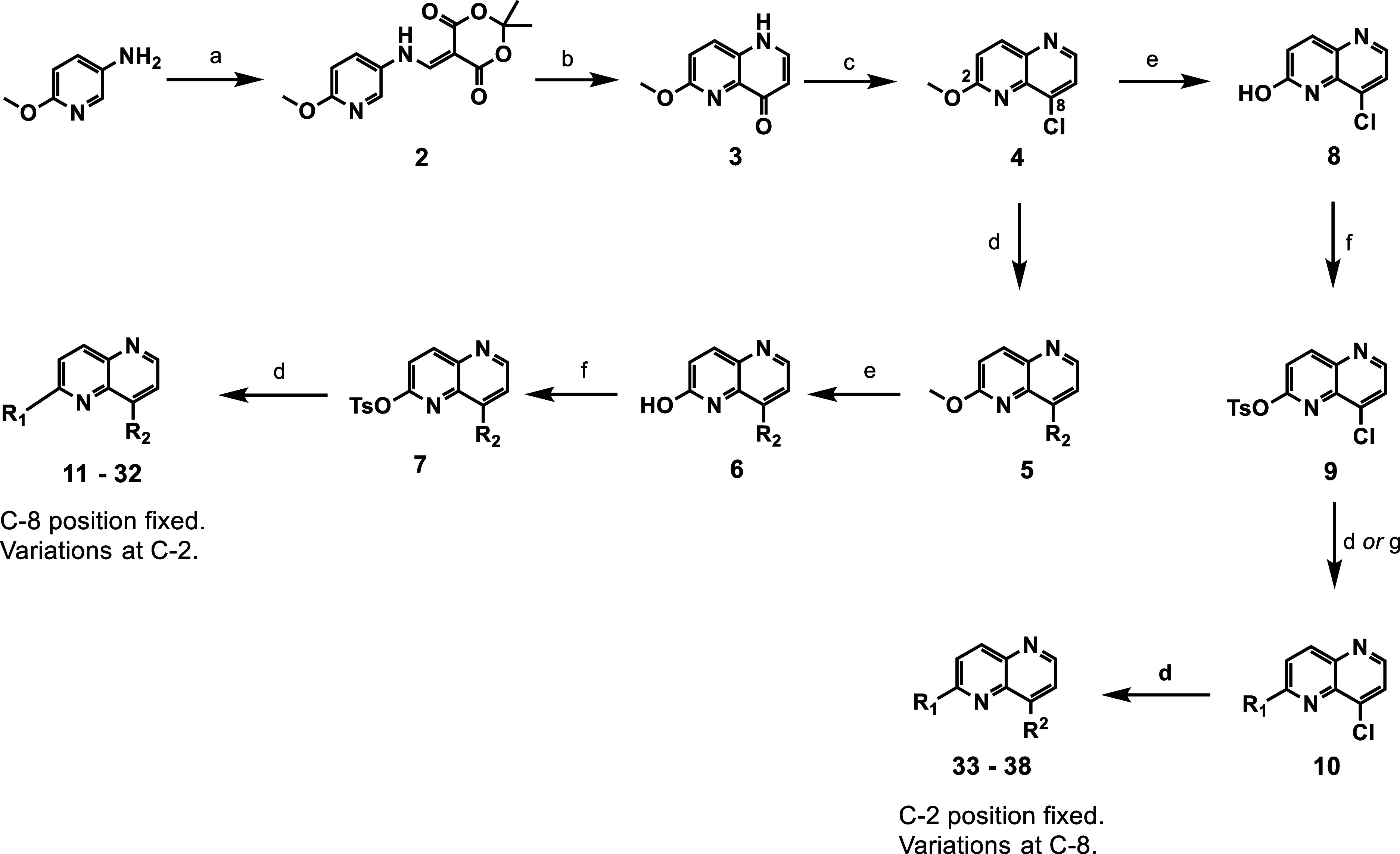
Synthesis of 2,5-Diaryl 1,5-Naphthyridines^a^

a Reagents and reaction conditions: (a) 2,2-dimethyl-1,3-dioxane-4,6-dione, trimethoxymethane, 105 °C, 12 h; (b) diphenyl ether, 220 °C, 2 h; (c) POCl_3_, 100 °C, 1 h; (d) appropriate boronic acid or pinacol ester, PdCl_2_(dppf), K_3_PO_4_ or Cs_2_CO_3_, dioxane/water (9:1), 90 °C or PdCl_2_(PPh_3_)_2_, K_2_CO_3_, DMF/water (9:1), 100 °C, 2 h; (e) conc. HCl, 80 °C, 18 h; (f) *p*-toluenesulfonyl chloride, triethylamine, DMAP, DCM, 20–30 °C, 2 h; (g) relevant amine, Cs_2_CO_3_, DMF, 90 °C, 18 h.

### SAR Exploration Strategies

To improve upon the potency of compound **1**, the published binding mode of the 2,8-disubstituted naphthyridine docked into a *Pf*PI4K homology model^16^ (Figure 2) was used to design new analogues with improved potencies via substitutions at the C-2 and C-8 positions of the 1,5-naphthyridine core. The 2,8 disubstituted 1,5-naphthyridine core is predicted to bind in an orientation that sees the 5-N accepting an H-bond from the backbone amide of the hinge residue V1357, while the C-2 phenyl sulfonamide substitution lies on a vector extending out toward the ribose pocket and the C-8 pyridyl CF_3_ substitution interacts with the catalytic site including K1308 and D1430. Aromatic π-stacking interactions between the heteroaromatic core and both the hinge Y1356 and F827 of the P-loop are predicted to enhance both the potency and specificity toward *Pf*PI4K. The pyridyl-CF_3_ interacts with a lipophilic region that lies near key kinase catalytic residues, K1308 and D1430.

**Figure 2:**
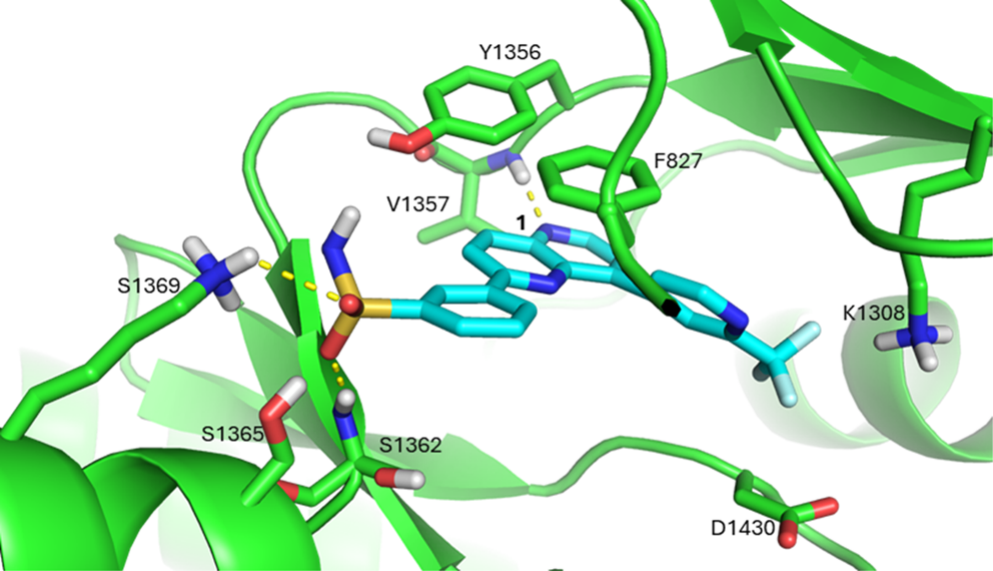
Compound **1** (cyan) docked into the ATP binding site of the *Pf*PI4K homology model (green). The *Pf*PI4K homology model was built off the *Hs*PI4K structure (PDB ID: 4D0L), as previously described.^16^

To improve enzymatic activity, the new analogues were designed to enhance the hydrogen bonding interactions with S1362 and S1365 in the ribose pocket by exploring groups with hydrogen donor/acceptor motifs as bioisosteres of the sulfonamide group in **1**. We investigated a similar analogy to maximize the catalytic site interactions with K1308. Here, we aimed to have an additional hydrogen donor/acceptor group to replace the lipophilic CF_3_ in **1**. The design strategy yielded analogues with increased polarity at both C-2 and C-8 positions of the 1,5-naphthyridine core relative to **1**.

### In Vitro Antiplasmodial Activity

The synthesized analogues were all evaluated for their in vitro antiplasmodial activity against drug-sensitive asexual blood stage (ABS) *P. falciparum* NF54 (*Pf*NF54) parasites, while selected analogues were also tested against the multidrug-resistant *P. falciparum* K1 (*Pf*K1) strain (Table 1). Most of the analogues retained activity against the K1 strain within 2–4-fold of NF54. The *Pf*PI4K enzymatic activity of the synthesized compounds were evaluated using recombinant *Plasmodium vivax*­(*Pv*) PI4K as a surrogate for *P. falciparum*. The catalytic domains of *Pf*PI4K and *Pv*PI4K share 97% sequence identity, and their ATP-binding sites are predicted to be identical.^12,17^

**Table 1:**
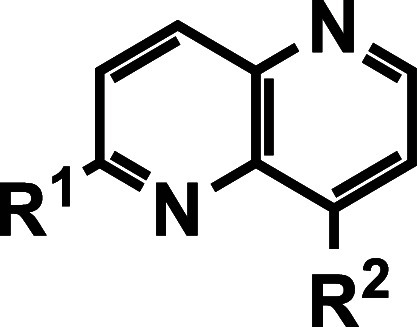

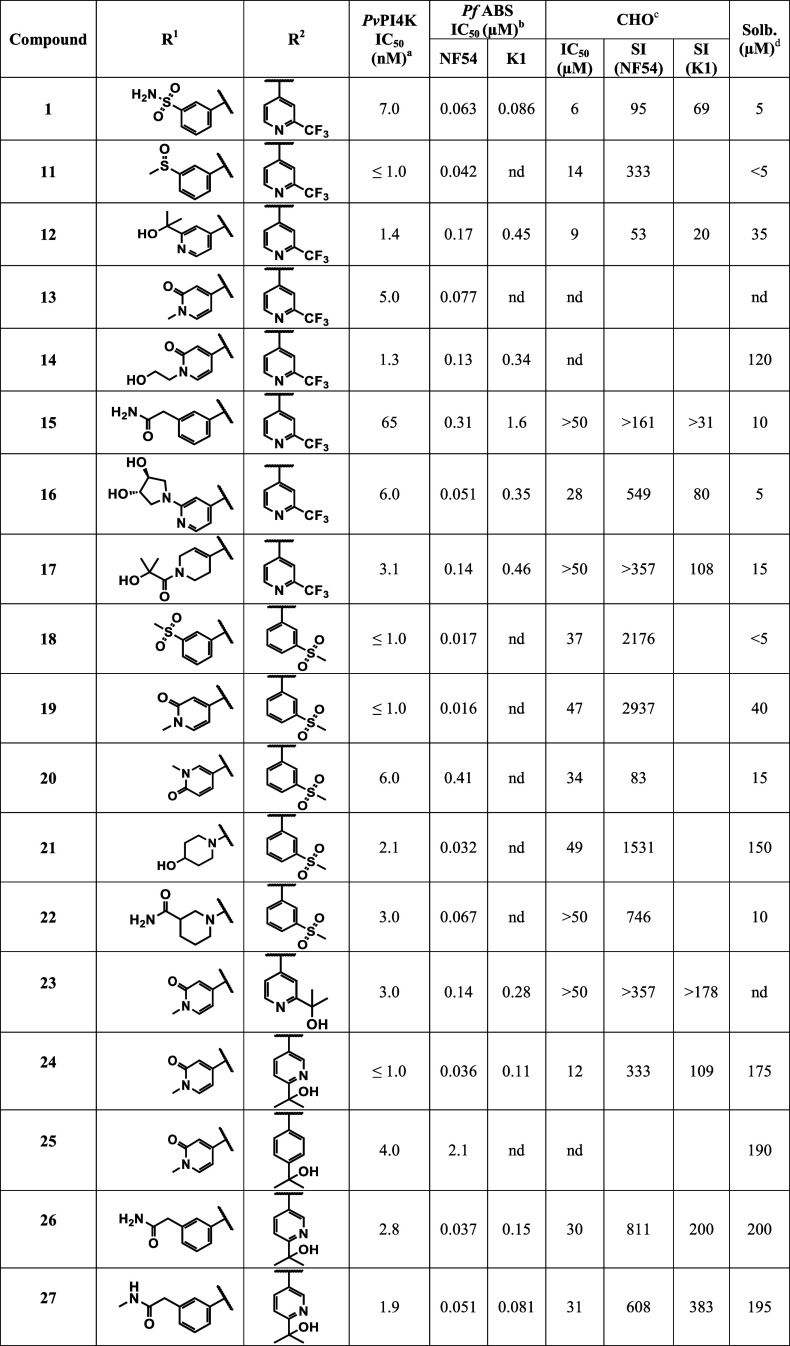

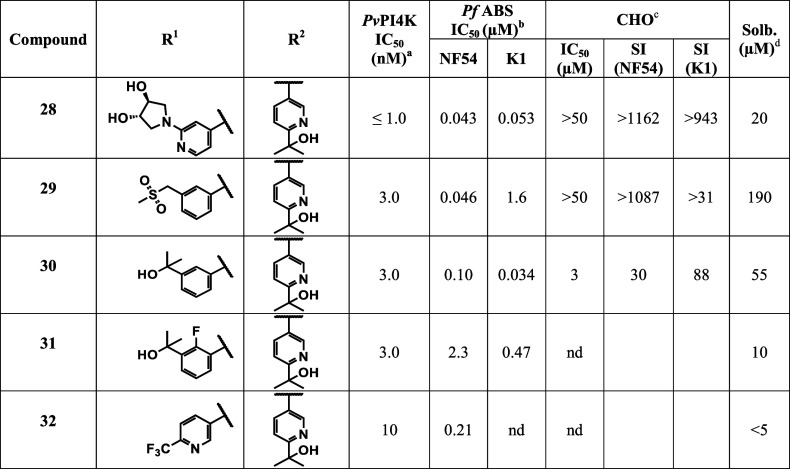
Modifications at C2-Position of 1,5-Naphthyridine Core Scaffold^e^

a Inhibition of purified recombinant *Pv*PI4K was determined in the presence of 10 μM ATP and ADP formation was quantified using the ADP-Glo kinase assay kit; sapanisertib at a concentration of 10 μM was used as the positive control (*Pv*PI4Kβ IC_50_ = 4 nM). Mean IC_50_ values were calculated based on *n* ≥ 2 independent experiments, each with technical duplicates. IC_50_ < 1 nM means below the limit of detection of the assay.

b IC_50_ values were determined using a lactate dehydrogenase (pLDH) assay for 72 h, with means calculated from 2 independent experiments, each with technical triplicates; Chloroquine (*Pf*NF54/*Pf*K1 IC_50_ = 11/143 nM) was used as positive control.

c Chinese hamster ovary cells tested as one biological replicate with technical triplicates; Emetine (IC_50_ < 3 nM) was used as positive control; SI (NF54) means selectivity index with respect to NF54 IC_50_; SI (K1) means selectivity index with respect to K1 IC_50_.

d Thermodynamic solubility (aqueous) at pH_6.5_. In vitro data (mean IC_50_ values ± standard deviation (SD)) are provided as Supporting Information file.

e nd: Not determined.

Like **1**, compounds **11**–**17** retained the C-8 trifluoromethylpyridyl group. Among these compounds, the racemic 3-sulfinyl analogue **11** (IC_50_ *Pv*PI4K/*Pf*NF54 ≤ 0.0010/0.042 μM) and the *N*-methyl 2-pyridone **13** (IC_50_ *Pv*PI4K/*Pf*NF54 = 0.0050/0.077 μM) displayed potent inhibition of *Pv*PI4K and comparable *Pf*NF54 activity to **1**. Similarly, the (3*R*,4*R*)-1-(pyridin-2-yl)­pyrrolidine-3,4-diol **16** (IC_50_ *Pv*PI4K/*Pf*NF54 = 0.0060/0.051 μM) displayed good potency. However, **12** with a 2-hydroxypropylpyridyl group at C2 showed lower activity (IC_50_ *Pv*PI4K/*Pf*NF54/K1 = 0.014/0.17/0.45 μM). Compared to **13**, the *N*-(2-hydroxyethyl) substituted 2-pyridone **14** retained *Pv*PI4K (IC_50_ = 1.3 nM) potency but displayed a ∼2-fold loss in ABS activity (IC_50_ *Pf*NF54/K1 = 0.13/0.34 μM), although solubility (120 μM) was significantly improved. The C2 acetamidophenyl group along with the C-8 trifluoromethylpyridyl group in **15** (IC_50_ *Pv*PI4K/*Pf*NF54/K1 = 0.065/0.31/1.6 μM) was less well tolerated. The aliphatic piperidine amide **17** showed comparable activity to the aromatic congener **12**. It is worth noting that **15**–**17** were predicted to extend much deeper into the ribose pocket of *Pf*PI4K abutting S1362 and S1365 rather than clashing with the similarly positioned and larger glutamine Q606 of the *Hs*PI4K, thereby achieving selectivity over the latter. However, only **16** retained potent *Pv*PI4K and *Pf*NF54 activities.

To improve potency, the C-2 modifications above were explored with a C-8 3-methylsulfonylphenyl group (**18**–**22**), which was predicted to interact with the catalytic K1308 of *Pf*PI4K. Comparatively, these analogues showed improved ABS activity and potent *Pv*PI4K inhibition, the latter near or at the lower limit of resolution by the assay, corroborating the supplementary interaction with K1308. For example, the 3-sulfonyl phenyl analogue **18**, like **11**, maintained good activity (IC_50_ *Pv*PI4K/*Pf*NF54 ≤ 0.0010/0.016 μM). Similarly, compared to **13**, the *N*-methyl 2-pyridone analogue **19** retained potent *Pv*PI4K inhibition (IC_50_ ≤ 1.0 nM) with ∼4-fold improved *Pf*NF54 activity (IC_50_ = 0.016 μM) and moderate aqueous solubility (40 μM). Interestingly, **20**, a regioisomer of **19**, showed a significant loss in *Pf*NF54 activity (IC_50_ 0.41 μM) compared to **19** while still retaining significant *Pv*PI4K inhibitor potency (IC_50_ 6.0 nM). Unlike **17**, the C2-piperidinyl analogues **21** and **22** showed improved potency against *Pv*PI4K, translating to improved *Pf*NF54 activity with IC_50_ values of 0.032 μM and 0.067 μM, respectively. Compound **21** showed significant improvement in solubility (IC_50_ = 150 μM).

The 2-hydroxypropyl substituent on the C8-aryl group was predicted to interact with the catalytic K1308, enhancing *Pv*PI4K inhibition while improving aqueous solubility in most cases as observed for analogues **23**–**32**. Comparison of **23**–**25** revealed that the 2-hydroxypropyl-3-pyridyl substituent of **24** was optimal for potency. The poor activity of **25** with the 3-(2-hydroxypropyl)­phenyl substituent (IC_50_ *Pv*PI4K/*Pf*NF54 = 0.004/2.1 μM) compared to **24** (IC_50_ *Pv*PI4K/*Pf*NF54 ≤ 0.0010/0.036 μM) shows the significant contribution of the pyridyl N to activity.

Like **15**–**17**, compounds **26**–**32** were synthesized to extend deeper into the ribose pocket of *Pf*PI4K to achieve selectivity over *Hs*PI4K. The acetamidophenyl (**26**) and methyl-acetamidophenyl (**27**) with the 2-hydroxypropyl-3-pyridyl at C8 showed enhanced *Pv*PI4K potency (IC_50_ ≈ 2 nM) and *Pf*NF54 activity (IC_50_ = 0.037 μM and 0.051 μM, respectively) with improved solubility (195–200 μM) compared to **15**, in line with the design that the 2-hydroxypropyl substituent on the C8 pyridine ring would enhance potency and increase solubility. Similarly, **28** and **29** retained *Pv*PI4K and *Pf*NF54 activities. Compounds **30**–**32** showed moderate to low *Pf*NF54 activity, although they showed good *Pv*PI4K potency (IC_50_ ≤ 0.010 μM).

In Table 2, with a 3-sulfonylphenyl group at C-2 position, more C-8 groups were explored. Interestingly, the *N*-alkyl-2-pyridones, **33** (*Pv*PI4K, IC_50_ = 19 nM) and **34** (*Pv*PI4K, IC_50_ = 39 nM), showed less inhibition of *Pv*PI4K compared to their congeners **19** and **20** (*Pv*PI4K, IC_50_ ≤ 6.0 nM) at C-2 position. Compounds **36**–**38**, with a 3-amino/hydroxypyrrolidinyl benzamide at the C-8 position, showed potent *Pv*PI4K inhibition (IC_50_ ≤ 2.0 nM) as predicted by the docking hypothesis. However, the 4-carboxamide motif at the C8 position (as in **37** and **38**) is preferred over the 3-regioisomer (as in **36**) for ABS activity.

**Table 2:**
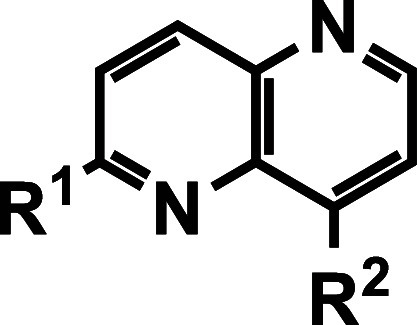

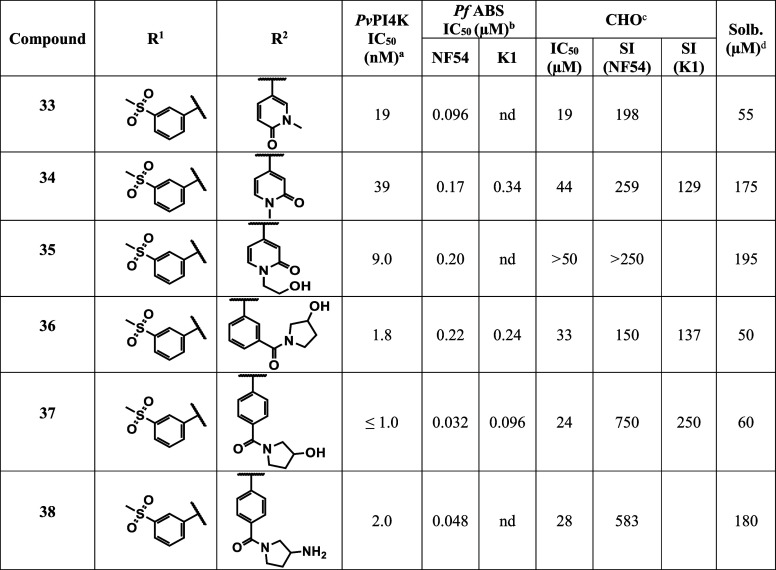
Modifications at C8-Position of 1,5-Naphthyridine Core Scaffold^e^

a Inhibition of purified recombinant *Pv*PI4K was determined in the presence of 10μM ATP and ADP formation was quantified using the ADP-Glo kinase assay kit; sapanisertib at a concentration of 10 μM was used as the positive control (*Pv*PI4Kβ IC_50_ = 4 nM). Mean IC_50_ values were calculated based on *n* ≥ 2 independent experiments, each with technical duplicates. IC_50_ < 1 nM means below the limit of detection of the assay.

b IC_50_ values were determined using a lactate dehydrogenase (pLDH) assay for 72 h, with means calculated from 2 independent experiments, each with technical triplicates; chloroquine (PfNF54/PfK1 IC_50_ = 11/143 nM) was used as positive control.

c Chinese hamster ovary cells tested as one biological replicate with technical triplicates; emetine (IC_50_ < 3 nM) was used as positive control. SI (NF54) means selectivity index with respect to NF54 IC_50_; SI (NF54) means selectivity index with respect to NF54 IC_50_.

d Thermodynamic solubility (aqueous) at pH6.5. In vitro data (Mean IC_50_ values ± standard deviation (SD)) are provided as Supporting Information file.

e nd: not determined.

### *Pf*PI4K as Primary Target

To further support *Pf*PI4K as the primary target, representative compounds were profiled against two laboratory generated mutant lines, PI4K S743F + H1484Y and PI4K S1320L + L1418F, which were selected using the PI4K inhibitor KAI407 (Figure 3).^12^ Compounds **1**, **27**, and **37** all showed IC_50_ shifts against the two mutant lines. These results are similar to those seen for KDU691 (positive control).

**Figure 3:**
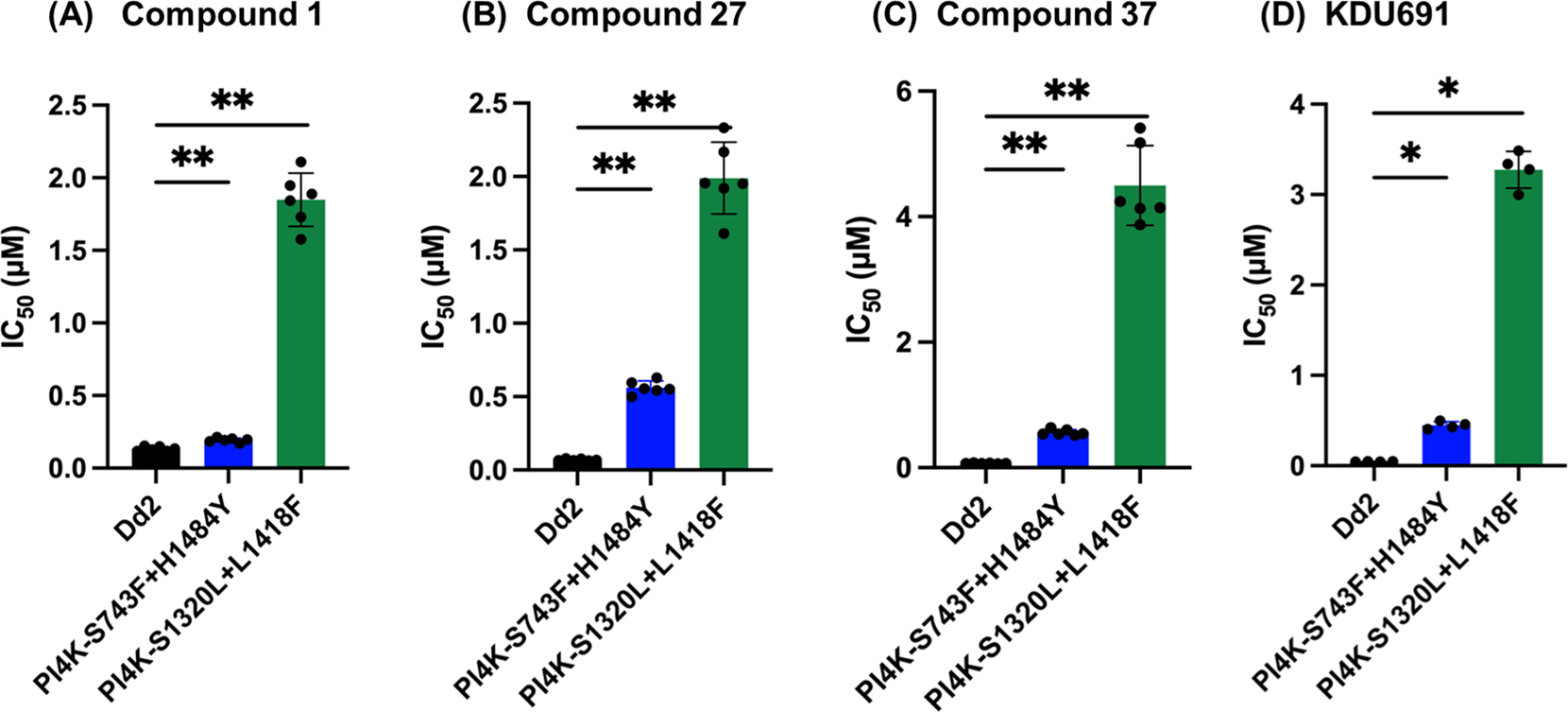
Profiling compounds (A) **1**, (B) **27**, (C) **37**, and (D) KDU691 against two PI4K lab mutant lines relative to parent Dd2 line. Data shown are from 4 to 6 biological replicates (with technical triplicates) and error bars show standard deviation.

Additionally, as previously described for **1** and MMV390048,^15^ compounds **19**, **21**, and **27** were profiled against a *Plasmodium* PI4K conditional knockdown (cKD) line in an asexual blood stage assay (Figure 4).^18^ The sensitivity of the cKD line to the compounds was evaluated in the presence or absence of anhydrotetracycline (aTc) using the TetR (Tet repressor protein)/DOZI (development of zygote inhibited)-RNA aptamer module.^19,20^ PI4K translation occurs in the presence of aTc but not in its absence (knockdown condition). In this study, like **1** and MMV390048, the new analogues **19**, **21**, and **27** showed a 7.6-fold, 6.0-fold, and 8.3-fold decrease in IC_50_, respectively, under the knockdown condition providing further confirmation that PI4K is the primary efficacious target of these compounds (Figure 4).

**Figure 4:**
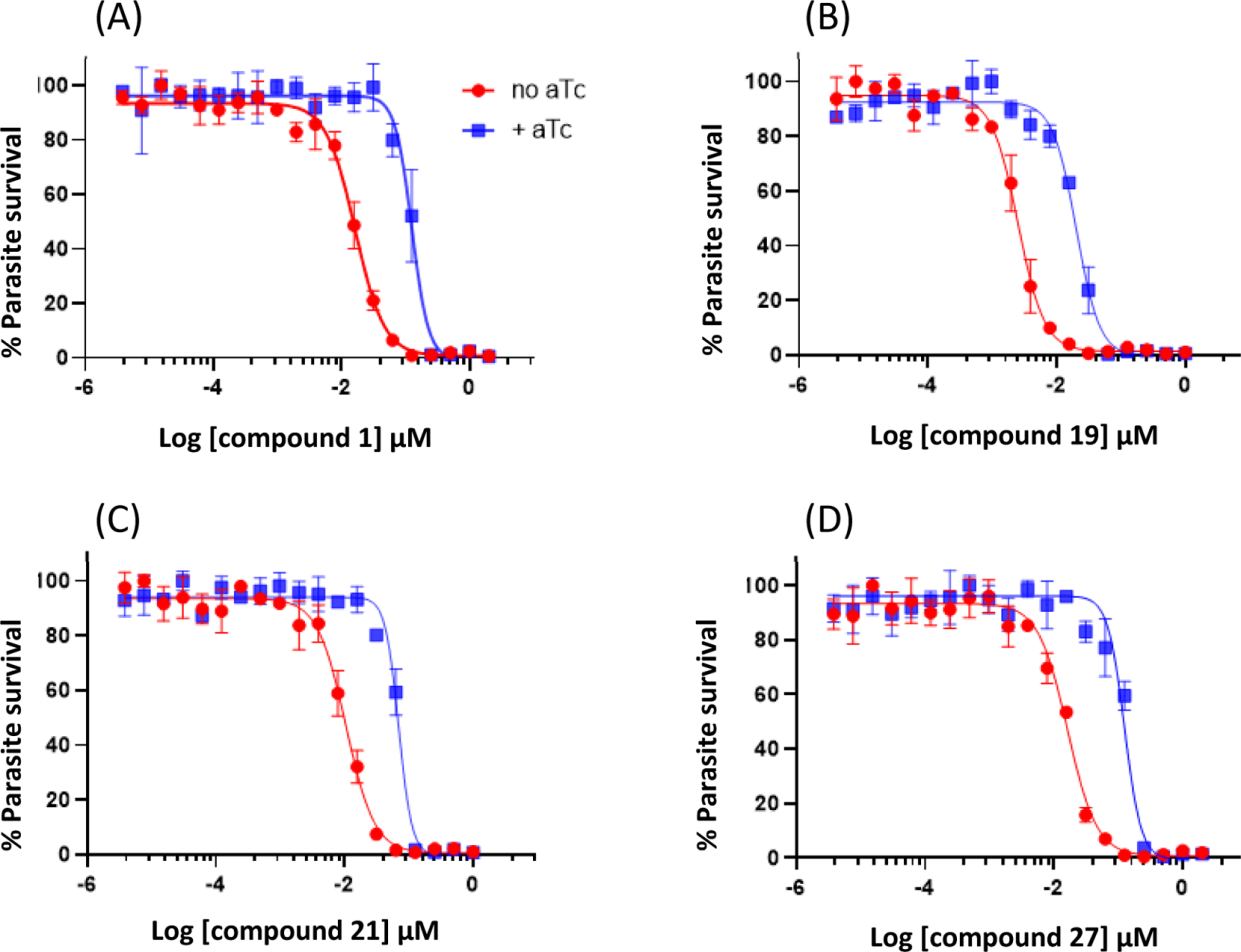
Profiling compounds (A) **1**, (B) **19**, (C) **21**, and (D) **27** against PI4K cKD line under aTc and no aTc conditions. Results were confirmed in *N* = 3 independent experiments. MMV390048 was used as positive control (graph not shown).^15^ Error bars show standard deviation for technical repeats.

### Cytotoxicity, hERG, and Human Kinase Profiling

The toxicity of the compounds to mammalian cells was assessed primarily against the Chinese hamster ovarian (CHO) cell line (Tables 1 and 2). Representative compounds (**21** and **27**) were also tested against the human hepatocellular carcinoma (HepG2) and rat skeletal muscle (L6) cell lines (Table 3). The compounds showed low cytotoxicity relative to *Pf*Nf54 activity, with selectivity indices (SI) > 100-fold. Compound **27** did not pose a cardiotoxicity risk due to inhibition of the human ether-a-go-go-related gene (hERG) potassium channel (Table 3). Inhibition of the hERG channel can lead to long QT syndrome (LQTS), a life-threatening cardiac arrhythmia that can cause sudden cardiac death.^21^

**Table 3:** Cytotoxicity, hERG, and Human Kinase Profiling^a^

compound	**1**	**13**	**21**	**22**	**27**	MMV390048
*Pf*NF54, IC_50_ (μM)	0.063	0.077	0.032	0.067	0.051	0.028
*Pv*PI4K, IC_50_ (μM)	0.0070	0.0048	0.0021	0.0025	0.0019	0.001
CHO, IC_50_ μM (SI)^a^	6 (95)	nd	49 (1531)	>50 (746)	31 (625)	>254 (>9071)
HepG2, IC_50_ μM (SI)^a^	48 (762)	nd	>50 (>1562)	nd	6 (104)	>100 (>3571)
L6, IC_50_ μM (SI)^a^	35 (555)	nd	8 (250)	nd	38 (791)	nd
hERG, IC_50_ μM (SI)^a^	>10	nd	nd	nd	>30 (>588)	>11 (>393)
*Hs*PI3Kα, IC_50_ μM (SI)^b^	nd	0.14 (29)	0.56 (66)	0.15 (60)	0.070 (37)	7.8 (7800)
*Hs*PI4Kβ, IC_50_ μM (SI)^b^	nd	0.55 (114)	0.040 (19)	0.14 (56)	0.13 (68)	1.0 (1000)
*Hs*MINK1, IC_50_ μM (SI)^b^	nd	>10 (>2083)	5.2 (2476)	>10 (>4000)	>10 (>5263)	0.74 (740)
*Hs*MAP4K4, IC_50_ μM (SI)^b^	nd	>10 (>2083)	2.8 (1333)	>10 (>4000)	>10 (>5263)	0.81 (810)

a nd: Not determined. Selectivity index (SI^a^ = IC_50_/*Pf*NF54 IC_50_; SI^b^ = IC_50_/*Pv*PI4K IC_50_); Inhibition of kinase experiments were conducted in the presence of 10 μM ATP concentration; CHO: Chinese Hamster Ovary cells; HepG2: human hepatoblastoma cell line; L6: rat myoblast cell line; hERG: human ether-a-go-go-related gene; *Hs*MINK1: human misshapen-like kinase 1; *Hs*MAP4K4: human mitogen-activated protein kinase kinase kinase kinase 4.

One of the aims of the current work was to identify *Pf*PI4K inhibitors with a better human kinase profile than MMV390048. The study also offered the opportunity to understand the toxicity profile of a different PI4K inhibitor scaffold and glean SAR, if any. The results are presented in Table 3. Compounds **13**, **21**, **22**, and **27** were tested against the key kinase off-targets hypothesized to be responsible for the teratogenicity observed for MMV390048 in rats.^14^ Compound **13** showed comparable *Pv*PI4K and *Pf*NF54 activity and shares a similar C-8 group with **1**, but the C-2 pyridone group of **13** has less interactions with residues (S1362 and S1365) in the ribose pocket compared to **1**. Compounds **21** and **22** share the same group at the C-8 position. The piperidinyl groups at the C-2 position introduces sp^3^ character in the scaffold, reducing the molecular planarity characteristic of many kinase inhibitors and associated with the promiscuity of these inhibitors. We hypothesized that these new analogues with sp^3^ character would be more specific to *Pf*PI4K. Moreover, the amide group of **22** afforded more ribose pocket interactions compared to **21** with just the hydroxyl group. Contrary to **13**, **21**, and **22**, compound **27** bears bulky groups on the C-2 and C-8 aromatic motifs. The bulky groups were designed and hypothesized to clash with the larger residues in the human ortholog of PI4K, especially in the ribose pocket, thereby improving the selectivity margins for *Pf*PI4K. Compound **27** was selected as a representative compound for congeners like **15**, **16**, and **29**.

In Table 3, the compounds displayed much higher selectivity against MINK1 and MAP4K4 than MMV390048 with IC_50_ values >10 μM for **13**, **22**, and **27** whereas **21** showed measurable inhibition below 10 μM. However, MMV390048 was less active against the human orthologous PI3Kα and PI4Kβ compared to the current compounds. The data seem to suggest that the current series shows a different toxicity profile compared to MMV390048.

### Pharmacokinetic Studies

The current series of compounds showed improved solubility compared to **1** (Table 4). Compounds **19** and **21** showed high A-B permeability and low efflux across the Caco-2 cell line, implying high bioavailability expected, contrary to what was observed (Table 4). Compound **37** displayed only moderate A-B permeability and a high efflux ratio. As ultimately an oral drug is essential for the treatment of malaria in the afflicted regions of the world, achieving high oral bioavailability will be key to progressing this compound series. All the compounds assessed in mice and human liver microsomes were stable with little or no metabolism over the time course of the assays (Table 4). However, the in vitro stability was not recapitulated in vivo as all the compounds showed higher unbound clearance in mice.^15^ These results demonstrate that there is an extra-hepatic clearance mechanism in mice, whether it is metabolism otherwise, biliary or renal extraction or gut efflux. The pharmacokinetic parameters of **27** were comparable to those of **1** in mice (Table 4). Though **27** showed lower unbound clearance compared to **1**, its oral bioavailability was comparable to **1**.

**Table 4:** In Vitro and In Vivo DMPK Profiles of Representative Compounds^a^

compound	**1**	**19**	**21**	**24**	**27**	**29**	**37**
*Pf*NF54, IC_50_ (μM)	0.063	0.016	0.032	0.036	0.051	0.046	0.032
Solb., pH 6.5 (μM)	<5	40	150	175	195	190	60
TPSA/log *D*	98/2.9	80/1.46	83/2.27	79/1.98	88/nd	93/nd	100/1.92
Caco-2 (*P*_app_ A > B (10^–6^ cm/s))	13	14	18	nd	nd	nd	1.5
efflux ratio	3	2.3	1.2	nd	nd	nd	32
Mic. CL_int,app_ h/m (mL/min/kg)	<13.4/<45.7	<13.4/<45.7	<13.4/45.7	<13.4/<45.7	<13.4/45.7	<13.4/<45.7	<13.4/<45.7
Mic. CL_int,u_ h/m (mL/min/kg)	<23.5/<80.2	<19.7/<67.2	<17.6/<60.1	<17.4/<59.4	<25.3/<86.2	<47.9/<163	<27.3/<93.2
in vivo mouse CL_b_ (mL/min/kg)	11	102	80	110	55	109	181
in vivo mouse CL_u_ (mL/min/kg)	407	300	533	379	263	519	362
*F* (%)	39	27	37	10	33	12	11

a Solb. = solubility; Mic. = in vitro microsomal stability; TPSA: topological polar surface area; CL_int,app_: intrinsic clearance; CL_b_: total body clearance determined from whole blood; CL_u_: unbound blood clearance determined from CL_b_ and plasma protein binding (assuming blood to plasma ratio of 1); *F* %: bioavailability; nd: not determined.

### In Vivo Efficacy Studies

In the *Pf*SCID mouse model of malaria infection, **1** showed only 80% reduction in parasitemia at 4 × 50 mg/kg QD doses.^11^ With improved in vivo clearance relative to **1** and other congeners (Table 4), **27** was tested in the same model with a similar regimen as examined for **1**. Oral administration of compound **27** started 3 days post infection. The blood samples from treated mice were screened for drug concentration and parasitemia every 24 h from day 3 until day 7 post infection to determine the percentage reduction in parasitemia compared to the untreated control group and the area under the curve (AUC_0–96 h_) levels at 4 × 50 mg/kg QD doses. The data demonstrated slightly improved efficacy for **27** with a 91% reduction in parasitemia (Figure 5A). A simulation of the free oral exposure levels of **27** compared to the free in vitro activity (free IC_50_ = 0.04 μM) is shown in Figure 5B and supports that the slight increase in *F*_abs_ (that is, 0.59 for **27** relative to 0.43 for **1**), results in a higher free exposure to exert its therapeutic effect. More sustained free drug levels that could be achieved with compounds of lower in vivo clearance given the high antiplasmodial activity are needed to expect a better outcome in the efficacy experiment.

**Figure 5:**
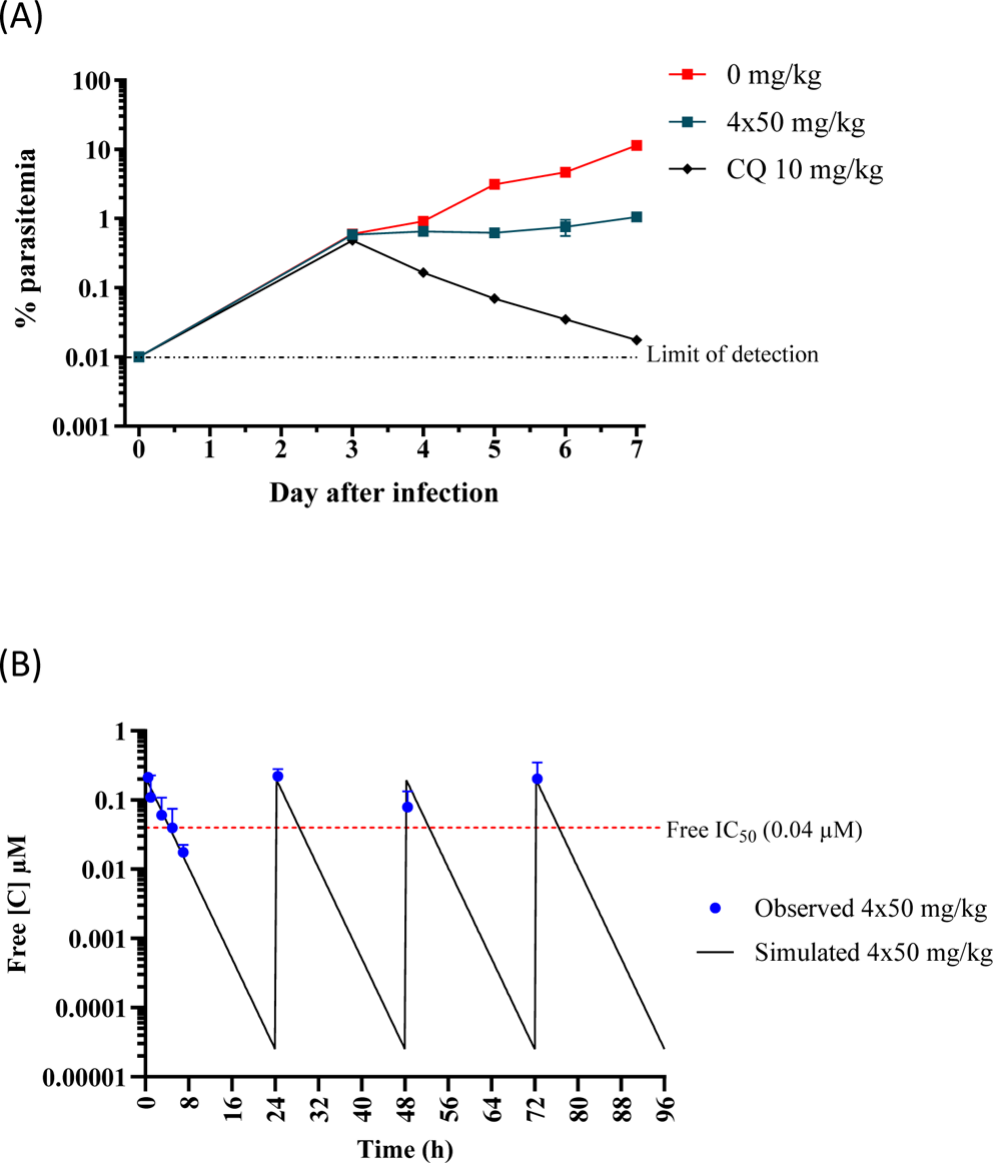
(A) In vivo therapeutic efficacy of **27** in humanized NSG mice infected with *P. falciparum* *Pf*3D7^0087/N9^ cells and (B) free circulating concentration of **27** following four consecutive oral doses of 50 mg/kg dosed 24 h apart. The solid line represents the simulated **27** concentration versus the observed **27** concentration data points displayed as blue dots. The red dashed line represents the free in vitro *Pf*NF54 IC_50_.

## Conclusion

A focused target-based strategy using the *P. falciparum* PI4K homology model was used to guide the design of new 2,8-diaryl-1,5-naphthyridine inhibitors. The compound design, which focused on improving catalytic site and ribose pocket interactions with hydrophilic inhibitor motifs, led to the identification of several compounds with improved potency against the *Plasmodium* kinase target and ABS parasites. The hydrophilic and polar groups afforded the complementary benefit of improving the physicochemical properties, especially aqueous solubility, of the compounds. While retaining *Plasmodium* PI4K as the primary target, the current series presents a different human kinase off-target toxicity profile compared to MMV390048, the clinical candidate whose development was discontinued due to the teratogenicity observed in rats. Frontrunner compound **27** showed significantly higher selectivity margins against the human MINK1 and MAP4K4 kinases, whose inhibition has been hypothesized to be responsible for the teratogenicity observed in rats. On the other hand, the low selectivity against related human lipid kinases (PI3Kα and PI4Kβ) needs to be addressed for the series. Notwithstanding this liability, compound **27** represents a good and alternate starting point for further optimization in the arena of *Pf*PI4K inhibitors toward achieving high antimalarial activity while mitigating off-target liabilities.

## Experimental Section

### Modeling

All docking simulations were run on the *Pf*PI4K homology.^16^ Protein structures were all processed using the Maestro GUI of the Schrodinger 2023-4 (Schrödinger Release 2023-4: Schrödinger, LLC, New York, NY, 2023.) software suite. Protein structures were prepared using the Schrodinger Protein Preparation wizard using default preprocessing, H-bond optimization and minimization settings. Crystallization artifacts were manually removed, and the residues with alternate positions were manually assigned. The prepared ligand binding site was then visually inspected for correct tautomer and H-bond assignment.

Docking grids were then prepared using the GLIDE Receptor Grid Generation tool with a grid centered upon previously docked ligands in the active site of the crystallized ligand from *Hs*PI4K.^22^ A hydrogen bonding constraint on the hinge H-bond donating amide (*Pf*PI4K–V1357) was also set.

All ligands were prepared from SMILES using LigPrep with ionization states determined to a pH of 7 ± 1.0, calculated using the Epik and the OPLS4 atomic force field.^23^ The prepared ligands were then docked into the prepared docking grids using SP docking. Ligands that failed to dock with a plausible pose were redocked at a higher precision with the top 10 poses retained. Every ligand for which a plausible docking pose was determined then had its binding energy scored using Prime MM-GBSA with the minimization radius set to full residues within 5 Å of the ligands using the VSGB implicit solvation model and the OPLS4 force field with the minimize sampling method.

All docking images were generated using open source PyMOL 2.5.0 (Schrodinger, LLC. 2010. The PyMOL Molecular Graphics System, Version 2.5.0).

### Chemistry

All commercially available reagents were purchased from Sigma-Aldrich or Combi-blocks. Unless otherwise stated, all solvents used were either anhydrous or analytical grade. Where stated, microwave synthesis was conducted using a Discover/Explorer12 microwave reactor from CEM Corporation. Column chromatography was performed using a Teledyne ISCO combi flash system in either normal (on prepacked (Silicycle) silica gel cartridges) or reverse phase (on prepacked Silicycle C18 cartridges) modes, while HPLC was performed on a Teledyne ISCO ACCQPrep HP150 system eluting a reverse phase (C18) solvent gradient with an appropriate solvent gradient (water and acetonitrile or methanol, with or without 0.1% formic acid). ^1^H NMR spectra were recorded on a Bruker Spectrometer at 300 MHz. ^13^C NMR spectra were recorded on a Bruker 400 or 600 MHz spectrometer. Chemical shifts are reported in parts per million (ppm) downfield from TMS as the internal standard. Coupling constants, *J*, are reported in Hertz (Hz). Standard acronyms representing multiplicity are used: br s = broad singlet, s = singlet, d = doublet, t = triplet, m = multiplet. Purity was determined using an Agilent 1260 Infinity binary pump, Agilent 1260 Infinity diode array detector (DAD), Agilent 1290 Infinity column compartment, Agilent 1260 Infinity standard autosampler, and Agilent 6120 quadrupole (single) mass spectrometer, equipped with ESI ionization source. All compounds tested for biological activity were confirmed to have ≥ 95% purity. LC purity analyses were performed using one of the following methods: **Method 1**: using a Kinetex 1.7 μM C-18 column, 1 μL injection volume, flow 1.2 mL/min; gradient: 5–100% B in 1.5 min (hold 0.4 min), 100–5% in 0.3 min (hold 0.5 min) (mobile phase A: 0.1% formic acid in H_2_O and mobile phase B: 0.1% formic acid in acetonitrile); **Method 2**: using a Kinetex 2.6 μM C-18 column, 2 μL injection volume, flow 0.7 mL/min; gradient: 15–100% B in 1.2 min (hold 3.3 min), 100–15% in 0.3 min (hold 1.2 min) (mobile phase A: 10 mM buffer (ammonium acetate/acetic acid) in H_2_O and mobile phase B: 10 mM buffer (ammonium acetate/acetic acid) in methanol). All synthesized intermediates were characterized by LCMS, while final compounds were confirmed by LCMS and, at least, a ^1^H NMR data.

Compounds **1** and intermediates **2–4**, **8**, and **9**, and key intermediates 8-chloro-2-(3-(methylsulfonyl)­phenyl)-1,5-naphthyridine (**10a**) and 8-chloro-2-(6-(trifluoromethyl)­pyridin-3-yl)-1,5-naphthyridine (**10b**) were synthesized as previously described (Supporting Information).^11,15^ Similarly, the synthesis of all other intermediates and their respective analytical data are also described in the Supporting Information. The analytical data of all final compounds with the last synthetic step are described below.

### General Synthesis Methods

Method A: the appropriate intermediate (1 equiv), boronic acid or (1.2 equiv), potassium phosphate tribasic (or cesium carbonate) (1.7 equiv) and [1,1′-Bis­(diphenylphosphino)-ferrocene] dichloropalladium­(II) (0.05 equiv) were placed in a reaction vessel and purged with nitrogen gas. To this mixture was added degassed 1,4-dioxane and degassed distilled water (9:1, 5 mL). The reaction mixture was stirred at 100–120 °C for 0.5–18 h (or irradiated under microwave conditions for 30 min at 120 °C, 200 W (dynamic mode)). When complete, the mixture was cooled to room temperature, filtered through a pad of Celite and concentrated under reduced pressure. The crude product was purified by either normal or reverse phase chromatography.

Method B: to a solution of intermediate IV (1 equiv) in DMF (5 mL) was added the relevant boronic acid/ester (1.2 equiv), and Bis­(triphenylphosphine)­palladium­(II) dichloride (0.05 equiv), followed by potassium acetate or potassium carbonate (dissolved in water (10% by volume)). The mixture was degassed by bubbling nitrogen through it. The reaction was heated at 90–110 °C until completion (0.5–2 h). The mixture was cooled to ambient temperature, filtered through a pad of Celite and concentrated under reduced pressure. The crude product was purified by either normal or reverse phase chromatography.

#### 2-(3-Methylsulfinylphenyl)-8-[2-(trifluoromethyl)­pyridin-4-yl]-1,5-naphthyridine (**11**)

To 2-bromo-8-[2-(trifluoromethyl)­pyridin-4-yl]-1,5-naphthyridine (100 mg, 0.28 mmol, synthesized as previously reported in ref 11) and 3-Methylsulfinylphenylboronic acid (57 mg, 0.31 mmol) according to Method A. Brown solid (71 mg, 60% yield); ^1^H NMR (300 MHz, DMSO-*d*_6_, δ): 9.16 (d, *J* = 4.4 Hz, 1H), 8.99 (d, *J* = 5.0 Hz, 1H), 8.69–8.56 (m, 2H), 8.54–8.48 (m, 1H), 8.45 (t, *J* = 1.6 Hz, 1H), 8.35 (dt, *J* = 1.4, 7.7 Hz, 1H), 8.30–8.21 (m, 1H), 8.09 (d, *J* = 4.4 Hz, 1H), 7.88–7.83 (m, 1H), 7.75 (t, *J* = 7.7 Hz, 1H), 2.81 (s, 3H). ^13^C NMR (151 MHz, DMSO): δ 155.6, 151.6, 150.2, 147.6, 146.2, 145.8, 143.2, 142.5, 139.6, 138.9, 138.7, 130.0, 129.5, 128.7, 125.1, 124.9, 122.6, 122.6, 122.5, 122.5, 43.2. LC–MS: *t*_R_ = 2.443 min (method 2, purity 99%); *m*/*z* = 414.1 [M + H]^+^ (anal. calcd for C_21_H_14_F_3_N_3_OS: *m*/*z* = 413.1).

#### 2-[4-[8-[2-(Trifluoromethyl)­pyridin-4-yl]-1,5-naphthyridin-2-yl]­pyridin-2-yl]­propan-2-ol (**12**)

Synthesized from **7a** (100 mg, 0.22 mmol) and [2-(2-hydroxypropan-2-yl)­pyridin-4-yl]­boronic acid (81 mg, 0.45 mmol) according to Method A. Yellow compound (9 mg, 10% yield); ^1^H NMR (400 MHz, DMSO-*d*_6_, δ): 9.21 (d, *J* = 4.2 Hz, 1H), 9.00 (d, *J* = 5.0 Hz, 1H), 8.72 (d, *J* = 8.8 Hz, 1H), 8.66 (d, *J* = 5.1 Hz, 1H), 8.59 (d, *J* = 8.8 Hz, 1H), 8.45 (d, *J* = 20.8 Hz, 2H), 8.30 (d, *J* = 5.2 Hz, 1H), 8.13 (d, *J* = 4.4 Hz, 1H), 7.97 (d, *J* = 5.0 Hz, 1H), 5.30 (s, 1H), 1.50 (s, 6H). ^13^C NMR (151 MHz, DMSO): δ 169.3, 155.0, 152.1, 150.1, 148.9, 146.3, 145.7, 145.1, 143.5, 142.8, 139.8, 139.1, 128.9, 125.1, 122.5, 122.4, 122.4, 119.1, 116.3, 72.4, 30.6 (2C). LC–MS: *t*_R_ = 1.193 min (method 1, purity 100%); *m*/*z* = 411.1 [M + H]^+^ (anal. calcd for C_22_H_17_F_3_N_4_O: *m*/*z* = 410.1).

#### 1-Methyl-4-[8-[2-(trifluoromethyl)­pyridin-4-yl]-1,5-naphthyridin-2-yl]­pyridin-2-one (**13**)

Synthesized from **13a** (85 mg, 0.31 mmol) and [2-(trifluoromethyl)­pyridin-4-yl]­boronic acid (72 mg, 0.38 mmol) according to Method A using dicyclohexylphosphino-2′,6′-diisopropoxybiphenyl (35 mg, 0.075 mmol) and Tris­(dibenzylideneacetone)­dipalladium(0) (28 mg, 0.031 mmol) to give **13** (28 mg, 22% yield) as a brown solid. ^1^H NMR (300 MHz, DMSO-*d*_6_, δ): 9.18 (d, *J* = 4.4 Hz, 1H), 8.99 (d, *J* = 5.0 Hz, 1H), 8.63 (d, *J* = 8.9 Hz, 1H), 8.57–8.43 (m, 2H), 8.19 (dd, *J* = 5.0, 1.6 Hz, 1H), 8.08 (d, *J* = 4.4 Hz, 1H), 7.82 (d, *J* = 7.1 Hz, 1H), 7.23 (d, *J* = 2.0 Hz, 1H), 6.90 (dd, *J* = 7.1, 2.0 Hz, 1H), 3.48 (s, 3H). ^13^C NMR (151 MHz, DMSO-*d*_6_): δ 162.5, 154.4, 152.6, 152.6, 148.4, 146.1, 143.2, 143.2, 140.5, 139.9, 139.3, 129.0, 129.0, 125.4, 123.7, 123.0, 117.8, 117.8, 103.1, 37.0. LC–MS (ESI): *t*_R_ = 0.965 min (method 1, purity 100%); *m*/*z* = 383.1 [M + H]^+^ (anal. calcd for C_20_H_13_F_3_N_4_O: *m*/*z* = 382.0).

#### 1-(2-Hydroxyethyl)-4-[8-[2-(trifluoromethyl)­pyridin-4-yl]-1,5-naphthyridin-2-yl]­pyridin-2-one (**14**)

Synthesized from **7a** (110 mg, 0.25 mmol) and [1-(2-hydroxyethyl)-2-oxopyridin-4-yl]­boronic acid (90 mg, 0.49 mmol) according to Method A to give **14** (18 mg, 17% yield) as a white solid. ^1^H NMR (400 MHz, DMSO-*d*_6_, δ): 9.19 (d, *J* = 4.4 Hz, 1H), 9.00 (d, *J* = 4.9 Hz, 1H), 8.64 (s, 1H), 8.53 (d, *J* = 8.9 Hz, 1H), 8.48 (s, 1H), 8.21 (dd, *J* = 5.0, 1.5 Hz, 1H), 8.10 (d, *J* = 4.4 Hz, 1H), 7.74 (d, *J* = 7.1 Hz, 1H), 7.24 (d, *J* = 2.0 Hz, 1H), 6.92 (dd, *J* = 7.1, 2.1 Hz, 1H), 4.93 (s, 1H), 3.99 (t, *J* = 5.4 Hz, 2H), 3.66 (d, *J* = 4.7 Hz, 2H). ^13^C NMR (151 MHz, DMSO): δ 161.7, 154.1, 152.2, 150.3, 148.0, 146.4, 145.7, 143.5, 142.9, 140.4, 139.6, 138.9, 128.6, 125.0, 122.6 (3), 117.5, 102.4, 58.6, 51.2. LC–MS: *t*_R_ = 0.996 min (method 1, purity 97%); *m*/*z* = 412.3 [M + H]^+^ (anal. calcd for C_21_H_15_F_3_N_4_O_2_: *m*/*z* = 413.1).

#### 2-[3-[8-[2-(Trifluoromethyl)­pyridin-4-yl]-1,5-naphthyridin-2-yl]­phenyl]­acetamide (**15**)

Synthesized from **7b** (100 mg, 0.22 mmol) and [3-(2-amino-2-oxoethyl)­phenyl]­boronic acid (52 mg, 0.29 mmol) according to Method B. White solid (14 mg, 15% yield) as a white solid. ^1^H NMR (300 MHz, DMSO-*d*_6_, δ): 9.38 (s, 1H), 9.12 (d, *J* = 4.4 Hz, 2H), 9.00 (d, *J* = 5.0 Hz, 2H), 8.24 (d, *J* = 5.1 Hz, 2H), 8.04 (s, 1H), 7.58 (s, 1H), 7.42 (d, *J* = 6.6 Hz, 2H), 6.98 (s, 1H), 6.68 (s, 1H), 4.23 (s, 2H). LC–MS: *t*_R_ = 2.478 min (method 2, purity 100%); *m*/*z* = 409.1 [M + H]^+^ (anal. calcd for C_22_H_15_F_3_N_4_O: *m*/*z* = 408.1).

#### (3*S*,4*S*)-1-[4-[8-[2-(Trifluoromethyl)­pyridin-4-yl]-1,5-naphthyridin-2-yl]­pyridin-2-yl]­pyrrolidine-3,4-diol (**16**)

A mixture of **16b** (100 mg, 0.27 mmol), (3*S*,4*S*)-pyrrolidine-3,4-diol (28 mg, 0.27 mmol), and sodium carbonate (114 mg, 1.08 mmol) in DMSO (2 mL) was heated at 120 °C for 24 h. The reaction filtered and the crude product was purified by HPLC on a Teledyne ISCO ACCQPrep HP150 system eluting a reverse phase solvent gradient of acetonitrile in water on a 20 × 250 mm C18 column to afford **16**. Yellow solid (58 mg, 47% yield); ^1^H NMR (300 MHz, DMSO-*d*_6_, δ): 9.17 (d, *J* = 4.4 Hz, 1H), 9.01 (d, *J* = 5.0 Hz, 1H), 8.66 (d, *J* = 8.9 Hz, 1H), 8.58 (d, *J* = 8.9 Hz, 1H), 8.47 (s, 1H), 8.26 (d, *J* = 5.1 Hz, 1H), 8.20 (d, *J* = 5.3 Hz, 1H), 8.08 (d, *J* = 4.4 Hz, 1H), 7.28 (d, *J* = 5.3 Hz, 1H), 7.15 (s, 1H), 5.20 (s, 2H), 4.08 (d, *J* = 3.6 Hz, 2H), 3.60 (dd, *J* = 11.2, 3.9 Hz, 2H), 3.39 (d, *J* = 11.1 Hz, 2H). ^13^C NMR (151 MHz, DMSO): δ 158.0, 155.4, 151.8, 150.2, 148.9, 146.3, 146.0, 145.5, 143.5, 142.8, 139.6, 138.7, 128.8, 124.9, 122.6, 122.5, 122.4, 108.7, 103.6, 74.4 (2C), 52.7 (2C). LC–MS: *t*_R_ = 0.706 min (method 1, purity 100%); *m*/*z* = 454.1 [M + H]^+^ (anal. calcd for C_23_H_18_F_3_N_5_O_2_: *m*/*z* = 453.1).

#### 2-Hydroxy-2-methyl-1-[4-[8-[2-(trifluoromethyl)­pyridin-4-yl]-1,5-naphthyridin-2-yl]-3,6-dihydro-2*H*-pyridin-1-yl]­propan-1-one (**17**)

Synthesized from **17c** (220 mg, 0.62 mmol) and 2-Hydroxyisobutyric acid (77 mg, 0.74 mmol) with HATU (469 mg, 1.23 mmol) and *N*,*N*-diisopropylethylamine (0.32 mL, 1.85 mmol) in DMF (5 mL). The reaction proceeded at 50 °C for 16 h. Purification was achieved by C18 reverse phase chromatography. Yellow solid (76 mg, 27% yield); ^1^H NMR (400 MHz, DMSO-*d*_6_, δ): 9.07 (d, *J* = 4.5 Hz, 1H), 8.96 (d, *J* = 5.0 Hz, 1H), 8.50 (s, 1H), 8.45 (d, *J* = 9.0 Hz, 1H), 8.18 (dd, *J* = 17.9, 7.0 Hz, 2H), 8.02 (d, *J* = 4.5 Hz, 1H), 6.98 (d, *J* = 3.6 Hz, 1H), 5.46 (s, 1H), 4.72 (s, 1H), 4.37–3.97 (m, 2H), 2.64 (s, 2H), 1.34 (s, 6H). LC–MS: *t*_R_ = 2.489 min (method 2, purity 100%); *m*/*z* = 443.1 [M + H]^+^ (anal. calcd for C_23_H_21_F_3_N_4_O_2_: *m*/*z* = 442.1).

#### 2,8-Bis­(3-methylsulfonylphenyl)-1,5-naphthyridine (**18**)

Intermediate **9** (50 mg, 0.15 mmol), dicyclohexyl­[2-(2,4,6-triisopropylphenyl)­phenyl]­phosphane (0.17 mg, 0.0004 mmol), 3-methylsulfonylphenylboronic acid (36 mg, 0.18 mmol), palladium­(II) acetate (0.07 mg, 0.0003 mmol) were placed in a sealed tube and degassed using nitrogen balloon for 5 min. Sparged 1-butanol (2 mL) was added to mixture and solution was stirred at 25 °C for 5 min before sodium hydroxide (5.9 mg, 0.15 mmol) in water (0.5 mL). The reaction was completed after 3 h at 25 °C. Excess butanol was removed in vacuo and the residue was extracted with ethyl acetate (2 × 100 mL) and water (100 mL). The organics layers were combined and dried over Magnesium sulfate, filtered and concentrated in vacuo to give a brown crude product which was purified by normal phase chromatography eluting a gradient of ethyl acetate in petroleum ether. Pale brown solid (16 mg, 24% yield); ^1^H NMR (600 MHz, DMSO-*d*_6_, δ): 9.14 (d, *J* = 4.4 Hz, 1H), 8.73 (t, *J* = 1.8 Hz, 1H), 8.68 (d, *J* = 8.8 Hz, 1H), 8.66–8.62 (m, 2H), 8.57 (t, *J* = 1.8 Hz, 1H), 8.33 (dt, *J* = 7.6, 1.3 Hz, 1H), 8.13 (ddd, *J* = 7.9, 1.9, 1.1 Hz, 1H), 8.09–8.01 (m, 2H), 7.89 (t, *J* = 7.8 Hz, 1H), 7.82 (t, *J* = 7.8 Hz, 1H), 3.36–3.30 (m, 6H). ^13^C NMR (151 MHz, DMSO-*d*_6_): δ 155.1, 152.2, 145.2, 143.8, 142.2, 141.2, 140.5, 139.4, 139.4, 137.4, 136.1, 132.8, 130.6, 129.8, 128.7, 127.5, 126.1, 125.3, 122.7, 43.9, 43.8, 40.5. LC–MS: *t*_R_ = 0.979 min (method 1, purity 100%); *m*/*z* = 439.1 [M + H]^+^ (anal. calcd for C_22_H_18_N_2_O_4_S_2_: *m*/*z* = 438.0).

#### 1-Methyl-4-[8-(3-methylsulfonylphenyl)-1,5-naphthyridin-2-yl]­pyridin-2-one (**19**)

Synthesized from **13a** (100 mg, 0.36 mmol) and 3-methylsulfonylphenylboronic acid (147 mg, 0.74 mmol) according to Method A. Beige solid (9 mg, 6% yield); ^1^H NMR (600 MHz, DMSO-*d*_6_, δ): 9.15 (d, *J* = 4.4 Hz, 1H), 8.62 (d, *J* = 8.8 Hz, 1H), 8.56 (t, *J* = 1.8 Hz, 1H), 8.51 (d, *J* = 8.9 Hz, 1H), 8.23 (dt, *J* = 7.8, 1.4 Hz, 1H), 8.11 (dt, *J* = 8.1, 1.3 Hz, 1H), 8.01 (d, *J* = 4.4 Hz, 1H), 7.89 (t, *J* = 7.8 Hz, 1H), 7.80 (d, *J* = 7.1 Hz, 1H), 7.25 (d, *J* = 2.0 Hz, 1H), 7.13–6.99 (m, 1H), 3.48 (s, 3H), 3.34 (s, 3H). ^13^C NMR (151 MHz, DMSO-*d*_6_): δ 162.5, 154.1, 152.6, 145.4, 144.0, 141.1, 140.4, 139.2, 138.0, 137.3, 135.8, 130.1, 128.5, 127.6, 125.9, 125.3, 122.7, 117.6, 103.6, 44.1, 37.0. LC–MS: *t*_R_ = 0.849 min (method 1, purity 100%); *m*/*z* = 392.0 [M + H]^+^ (anal. calcd for C_21_H_17_N_3_O_3_S: *m*/*z* = 391.0).

#### 1-Methyl-5-[8-(3-methylsulfonylphenyl)-1,5-naphthyridin-2-yl]­pyridin-2-one (**20**)

Synthesized from intermediate **7b** (120 mg, 0.26 mmol) and *N*-methyl-1*H*-pyridin-2-one-5-boronic acid, pinacol ester (74 mg, 0.32 mmol) according to Method A. Brown solid (20 mg, 19% yield). ^1^H NMR (300 MHz, DMSO-*d*_6_, δ): 9.03 (d, *J* = 4.5 Hz, 1H), 8.76–8.62 (m, 2H), 8.52 (d, *J* = 8.9 Hz, 1H), 8.37–8.27 (m, 2H), 8.22 (d, *J* = 7.7 Hz, 1H), 8.14–8.05 (m, 1H), 7.95 (d, *J* = 4.5 Hz, 1H), 7.88 (t, *J* = 7.8 Hz, 1H), 6.49 (d, *J* = 9.5 Hz, 1H), 3.57 (s, 3H), 3.33 (s, 4H). ^13^C NMR (151 MHz, DMSO-*d*_6_): δ 162.0, 154.0, 150.9, 144.2, 143.3, 141.0, 140.9, 140.3, 138.8, 138.5, 137.5, 135.5, 130.2, 129.9, 127.4, 124.9, 121.1, 119.4, 116.8, 43.9, 37.8. LC–MS: *t*_R_ = 2.348 min (method 2, purity 100%); *m*/*z* = 392.0 [M + H]^+^ (anal. calcd for C_21_H_17_N_3_O_3_S: *m*/*z* = 391.0).

#### 1-[8-(3-Methylsulfonylphenyl)-1,5-naphthyridin-2-yl]­piperidin-4-ol (**21**)

Synthesized from **21a** (100 mg, 0.379 mmol) and 3-methylsulfonylphenylboronic acid (114 mg, 0.57 mmol) according to Method A. Yellow solid (108 mg, 74% yield); ^1^H NMR (300 MHz, DMSO-*d*_6_, δ): 8.65 (d, *J* = 4.5 Hz, 1H), 8.59 (s, 1H), 8.19–8.07 (m, 2H), 8.01 (d, *J* = 7.9 Hz, 1H), 7.80 (t, *J* = 7.8 Hz, 1H), 7.70 (d, *J* = 4.5 Hz, 1H), 7.54 (d, *J* = 9.5 Hz, 1H), 4.70 (d, *J* = 4.2 Hz, 1H), 4.21–4.07 (m, 2H), 3.82–3.69 (m, 1H), 3.26 (s, 3H), 1.88–1.74 (m, 2H), 1.48–1.32 (m, 2H). ^13^C NMR (101 MHz, DMSO-*d*_6_): δ 156.8, 145.9, 140.8, 140.8, 140.6, 140.2, 139.0, 138.5, 135.2, 129.6, 129.4, 126.8, 124.3, 113.9, 66.4, 44.1, 42.9, 34.4. LC–MS: *t*_R_ = 0.717 min (method 1, purity 100%); *m*/*z* = 384.1 [M + H]^+^ (anal. calcd for C_20_H_21_N_3_O_3_S: *m*/*z* = 383.1).

#### 1-[8-(3-Methylsulfonylphenyl)-1,5-naphthyridin-2-yl]­piperidine-3-carboxamide (**22**)

Synthesized from **22a** (75 mg, 0.26 mmol) and 3-methylsulfonylphenylboronic acid (77 mg, 0.39 mmol) according to Method A. Beige powder (35 mg, 33% yield); ^1^H NMR (300 MHz, DMSO-*d*_6_, δ): 8.65 (d, *J* = 4.5 Hz, 1H), 8.44 (s, 1H), 8.23 (d, *J* = 7.8 Hz, 1H), 8.12 (d, *J* = 9.4 Hz, 1H), 7.99 (d, *J* = 7.8 Hz, 1H), 7.76 (t, *J* = 7.8 Hz, 1H), 7.69 (d, *J* = 4.6 Hz, 1H), 7.56 (d, *J* = 9.5 Hz, 1H), 7.31 (s, 1H), 6.85 (s, 1H), 4.49–4.32 (m, 2H), 3.27 (s, 3H), 3.00 (dt, *J* = 25.3, 12.1 Hz, 2H), 2.43–2.27 (m, 1H), 1.97–1.82 (m, 1H), 1.78–1.63 (m, 2H), 1.53–1.39 (m, 1H). ^13^C NMR (101 MHz, DMSO-*d*_6_): δ 174.8, 156.2, 145.4, 140.5, 140.4, 140.1, 139.7, 138.5, 138.0, 135.0, 128.9, 128.4, 126.3, 123.8, 113.5, 47.4, 44.9, 43.5, 41.6, 27.7, 23.9. LC–MS: *t*_R_ = 0.717 min (method 1, purity 100%); *m*/*z* = 411.1 [M + H]^+^ (anal. calcd for C_21_H_22_N_4_O_3_S: *m*/*z* = 410.1).

#### 4-[8-[2-(2-Hydroxypropan-2-yl)­pyridin-4-yl]-1,5-naphthyridin-2-yl]-1-methylpyridin-2-one (**23**)

Synthesized from **13a** (70 mg, 0.26 mmol) and [2-(2-hydroxypropan-2-yl)­pyridin-4-yl]­boronic acid (70 mg, 0.39 mmol) according to Method B. White solid (8 mg, 8% yield); ^1^H NMR (300 MHz, DMSO-*d*_6_, δ): 8.29 (d, *J* = 4.5 Hz, 1H), 7.89 (d, *J* = 5.1 Hz, 1H), 7.78 (d, *J* = 8.8 Hz, 1H), 7.59 (d, *J* = 8.9 Hz, 1H), 7.43 (s, 1H), 7.14 (d, *J* = 4.5 Hz, 1H), 6.95 (d, *J* = 6.4 Hz, 2H), 6.51 (s, 1H), 6.43 (d, *J* = 7.1 Hz, 1H), 3.86 (s, 1H), 2.82 (s, 3H), 0.87 (s, 6H). LC–MS: *t*_R_ = 2.297 min (method 2, purity 100%); *m*/*z* = 372.2 [M + H]^+^ (anal. calcd for C_22_H_20_N_4_O_2_: *m*/*z* = 373.2).

#### 4-[8-[6-(2-Hydroxypropan-2-yl)­pyridin-3-yl]-1,5-naphthyridin-2-yl]-1-methylpyridin-2-one (**24**)

Synthesized from **13a** (100 mg, 0.37 mmol) and [6-(2-hydroxypropan-2-yl)­pyridin-3-yl]­boronic acid (133 mg, 0.74 mmol) according to Method A. Brown solid (35 mg, 25% yield); ^1^H NMR (400 MHz, DMSO-*d*_6_, δ): 9.10 (dd, *J* = 4.4, 1.3 Hz, 1H), 8.98 (t, *J* = 1.6 Hz, 1H), 8.58 (dd, *J* = 8.8, 1.3 Hz, 1H), 8.46 (dd, *J* = 8.9, 1.3 Hz, 1H), 8.33 (dt, *J* = 8.2, 1.8 Hz, 1H), 7.96 (dd, *J* = 4.4, 1.3 Hz, 1H), 7.87 (dd, *J* = 7.9, 3.7 Hz, 2H), 7.21 (d, *J* = 1.7 Hz, 1H), 6.95 (dt, *J* = 7.1, 1.6 Hz, 1H), 5.40 (d, *J* = 1.3 Hz, 1H), 3.48 (d, *J* = 1.3 Hz, 3H), 1.54 (d, *J* = 1.4 Hz, 6H). ^13^C NMR (151 MHz, DMSO-*d*_6_): δ 168.9, 162.5, 154.1, 152.5, 149.5, 148.7, 144.5, 143.9, 140.7, 140.6, 139.1, 139.1, 129.8, 125.0, 122.7, 118.2, 117.5, 103.4, 72.9, 40.4, 40.2, 40.1, 39.9, 39.8, 39.7, 39.5, 37.0, 31.1. LC–MS: *t*_R_ = 0.726 min (method 1, purity 100%); *m*/*z* = 373.2 [M + H]^+^ (anal. calcd for C_22_H_20_N_4_O_2_: *m*/*z* = 372.2).

#### 4-[8-[4-(2-Hydroxypropan-2-yl)­phenyl]-1,5-naphthyridin-2-yl]-1-methylpyridin-2-one (**25**)

Synthesized from **13a** (98 mg, 0.36 mmol) and 2-[4-(4,4,5,5-tetramethyl-1,3,2-dioxaborolan-2-yl)­phenyl]­propan-2-ol (113 mg, 0.43 mmol) according to Method B. White solid (60 mg, 44% yield); ^1^H NMR (300 MHz, DMSO-*d*_6_, δ): 9.06 (d, *J* = 4.5 Hz, 1H), 8.56 (d, *J* = 8.8 Hz, 1H), 8.45 (d, *J* = 8.8 Hz, 1H), 7.87 (d, *J* = 7.0 Hz, 4H), 7.67 (d, *J* = 8.0 Hz, 2H), 7.24 (d, *J* = 2.0 Hz, 1H), 6.99 (dd, *J* = 7.1, 2.1 Hz, 1H), 5.21 (s, 1H), 3.48 (s, 3H), 1.52 (s, 6H). ^13^C NMR (151 MHz, DMSO): δ 162.1, 153.3, 152.0, 151.3, 148.4, 146.9, 143.6, 140.5, 140.1, 138.7, 133.7, 130.3 (2C), 124.4 (2C), 122.0, 117.0, 103.1, 70.7, 36.6, 31.9 (2C). LC–MS: *t*_R_ = 0.890 min (method 1, purity 100%); *m*/*z* = 372.2 [M + H]^+^ (anal. calcd for C_23_H_21_N_3_O_2_: *m*/*z* = 371.2).

#### 2-[3-[8-[6-(2-Hydroxypropan-2-yl)­pyridin-3-yl]-1,5-naphthyridin-2-yl]­phenyl]­acetamide (**26**)

Synthesized from **26a** (120 mg, 0.38 mmol) and [6-(2-hydroxypropan-2-yl)­pyridin-3-yl]­boronic acid (82 mg, 0.45 mmol) according to Method B. White solid (30 mg, 19% yield); ^1^H NMR (300 MHz, DMSO-*d*_6_, δ): 9.06 (d, *J* = 4.4 Hz, 1H), 9.00 (d, *J* = 2.2 Hz, 1H), 8.59 (d, *J* = 8.7 Hz, 1H), 8.48–8.37 (m, 2H), 8.15–8.03 (m, 2H), 7.96 (d, *J* = 4.5 Hz, 1H), 7.90 (d, *J* = 8.2 Hz, 1H), 7.58 (s, 1H), 7.49 (t, *J* = 7.6 Hz, 1H), 7.40 (d, *J* = 7.6 Hz, 1H), 6.97 (s, 1H), 5.41 (s, 1H), 3.50 (s, 2H), 1.55 (s, 6H). ^13^C NMR (151 MHz, DMSO-*d*_6_): δ 172.4, 168.7, 156.8, 151.5, 149.4, 144.1, 143.6, 140.9, 139.3, 138.9, 138.4, 137.7, 131.1, 130.0, 129.2, 128.7, 125.8, 124.6, 122.5, 118.2, 72.9, 42.7, 31.1 (2C). LC–MS: *t*_R_ = 0.713 min (method 1, purity 100%); *m*/*z* = 399.2 [M + H]^+^ (anal. calcd for C_24_H_22_N_4_O_2_: *m*/*z* = 398.2).

#### 2-[3-[8-[6-(2-Hydroxypropan-2-yl)­pyridin-3-yl]-1,5-naphthyridin-2-yl]­phenyl]-*N*-methylacetamide (**27**)

Synthesized from **27a** (120 mg, 0.38 mmol) and [6-(2-hydroxypropan-2-yl)­pyridin-3-yl]­boronic acid (84 mg, 0.46 mmol) according to Method B. Yellow solid (67 mg, 42% yield); ^1^H NMR (300 MHz, DMSO-*d*_6_, δ): 9.06 (d, *J* = 4.4 Hz, 1H), 9.00 (d, *J* = 2.2 Hz, 1H), 8.58 (d, *J* = 8.8 Hz, 1H), 8.47–8.37 (m, 2H), 8.14–8.00 (m, 3H), 7.96 (d, *J* = 4.4 Hz, 1H), 7.90 (d, *J* = 8.2 Hz, 1H), 7.48 (t, *J* = 7.6 Hz, 1H), 7.39 (d, *J* = 7.6 Hz, 1H), 5.40 (s, 1H), 3.51 (s, 2H), 2.61 (d, *J* = 4.5 Hz, 3H), 1.55 (s, 6H). ^13^C NMR (151 MHz, DMSO-*d*_6_): δ 170.7, 168.7, 156.8, 151.5, 149.4, 144.1, 143.6, 140.9, 139.2, 138.9, 138.5, 137.6, 131.0, 130.0, 129.3, 128.7, 125.9, 124.7, 122.5, 118.2, 72.9, 42.8, 31.1 (2C), 26.1. LC–MS: *t*_R_ = 0.819 min (method 1, purity 100%); *m*/*z* = 413.2 [M + H]^+^ (anal. calcd for C_25_H_24_N_4_O_2_: *m*/*z* = 412.4).

#### (3*S*,4*S*)-1-[4-[8-[6-(2-Hydroxypropan-2-yl)­pyridin-3-yl]-1,5-naphthyridin-2-yl]­pyridin-2-yl]­pyrrolidine-3,4-diol (**28**)

Synthesized from **28a** (75 mg, 0.21 mmol) and (*3S*, *4S*)-pyrrolidine-3,4-diol (86 mg, 0.83 mmol) according to method described for **16** above. Yellow solid (65 mg, 69% yield); ^1^H NMR (300 MHz, DMSO-*d*_6_, δ): 9.09 (dd, *J* = 6.7, 3.3 Hz, 2H), 8.65–8.50 (m, 2H), 8.35 (dd, *J* = 8.3, 1.7 Hz, 1H), 8.23 (d, *J* = 5.3 Hz, 1H), 7.99 (d, *J* = 4.5 Hz, 1H), 7.90 (d, *J* = 8.2 Hz, 1H), 7.34 (d, *J* = 5.4 Hz, 1H), 7.28 (s, 1H), 5.40 (s, 1H), 5.17 (s, 2H), 4.07 (d, *J* = 3.5 Hz, 2H), 3.61 (dd, *J* = 11.3, 3.8 Hz, 2H), 3.47–3.38 (m, 1H), 3.20–3.14 (m, 1H), 1.56 (s, 6H). ^13^C NMR (101 MHz, DMSO-*d*_6_): δ 168.8, 158.4, 155.0, 152.2, 150.0, 149.4, 145.8, 144.5, 144.0, 140.7, 139.0, 129.8, 124.7, 122.4, 118.3, 109.1, 104.0, 74.9 (2C), 72.9, 53.2 (2C), 31.1 (2C). LC–MS: *t*_R_ = 0.562 min (method 1, purity 99%); *m*/*z* = 444.2 [M + H]^+^ (anal. calcd for C_25_H_25_N_5_O_3_: *m*/*z* = 443.2).

#### 2-[5-[6-[3-(Methylsulfonylmethyl)­phenyl]-1,5-naphthyridin-4-yl]­pyridin-2-yl]­propan-2-ol (**29**)

Synthesized from **29a** (100 mg, 0.29 mmol) and [6-(2-hydroxypropan-2-yl)­pyridin-3-yl]­boronic acid (65 mg, 0.36 mmol) according to Method B. White solid (49 mg, 37% yield); ^1^H NMR (300 MHz, DMSO-*d*_6_, δ): 9.12–9.05 (m, 1H), 9.01 (d, *J* = 2.2 Hz, 1H), 8.62 (d, *J* = 8.8 Hz, 1H), 8.42 (dd, *J* = 8.5, 2.4 Hz, 2H), 8.25 (s, 1H), 8.21 (d, *J* = 7.3 Hz, 1H), 7.97 (d, *J* = 4.5 Hz, 1H), 7.89 (d, *J* = 8.2 Hz, 1H), 7.66–7.52 (m, 2H), 5.38 (s, 1H), 4.60 (s, 2H), 2.96 (s, 3H), 1.55 (s, 6H). ^13^C NMR (101 MHz, DMSO-*d*_6_): δ 168.8, 156.4, 151.7, 149.4, 144.1, 143.6, 140.9, 139.4, 139.1, 138.9, 132.9, 130.5, 130.4, 130.0, 129.7, 127.8, 124.8, 122.6, 118.3, 72.9, 59.9, 31.1 (2C). LC–MS: *t*_R_ = 0.742 min (method 1, purity 100%); *m*/*z* = 434.1 [M + H]^+^ (anal. calcd for C_24_H_23_N_3_O_3_S: *m*/*z* = 433.1).

#### 2-[3-[8-[6-(2-Hydroxypropan-2-yl)­pyridin-3-yl]-1,5-naphthyridin-2-yl]­phenyl]­propan-2-ol (**30**)

Synthesized from **30a** (100 mg, 0.33 mmol) and [6-(2-hydroxypropan-2-yl)­pyridin-3-yl]­boronic acid (73 mg, 0.40 mmol) according to Method B. White solid (64 mg, 47% yield); ^1^H NMR (300 MHz, DMSO-*d*_6_, δ): 9.06 (d, *J* = 3.4 Hz, 2H), 8.57 (d, *J* = 8.8 Hz, 1H), 8.47 (d, *J* = 8.9 Hz, 1H), 8.42–8.33 (m, 2H), 8.05 (d, *J* = 7.7 Hz, 1H), 7.95 (d, *J* = 4.5 Hz, 1H), 7.88 (d, *J* = 8.2 Hz, 1H), 7.67 (d, *J* = 7.7 Hz, 1H), 7.49 (t, *J* = 7.7 Hz, 1H), 5.38 (s, 1H), 5.14 (s, 1H), 1.54 (s, 6H), 1.49 (s, 6H). ^13^C NMR (101 MHz, DMSO-*d*_6_): δ 168.7, 156.9, 151.7, 151.5, 149.8, 144.2, 143.6, 140.8, 139.1, 138.8, 137.8, 130.0, 128.9, 126.9, 125.4, 124.6, 124.2, 122.4, 118.2, 72.9, 71.1, 32.3 (2C), 31.1 (2C). LC–MS: *t*_R_ = 0.830 min (method 1, purity 100%); *m*/*z* = 400.2 [M + H]^+^ (anal. calcd for C_25_H_25_N_3_O_2_: *m*/*z* = 399.2).

#### 2-[2-Fluoro-3-[8-[6-(2-hydroxypropan-2-yl)­pyridin-3-yl]-1,5-naphthyridin-2-yl]­phenyl]­propan-2-ol (**31**)

Synthesized from **31a** (100 mg, 0.32 mmol) and [6-(2-hydroxypropan-2-yl)­pyridin-3-yl]­boronic acid (68 mg, 0.38 mmol) according to Method B. White solid (60 mg, 45% yield); ^1^H NMR (300 MHz, DMSO-*d*_6_, δ): 9.10 (d, *J* = 4.4 Hz, 1H), 9.00 (d, *J* = 2.2 Hz, 1H), 8.64–8.54 (m, 1H), 8.43–8.30 (m, 1H), 8.17 (dd, *J* = 8.8, 2.7 Hz, 1H), 7.96 (d, *J* = 4.4 Hz, 1H), 7.87–7.69 (m, 3H), 7.33 (t, *J* = 7.7 Hz, 1H), 5.42 (s, 1H), 5.36 (s, 1H), 1.56 (s, 6H), 1.51 (s, 6H). ^13^C NMR (151 MHz, DMSO): δ 168.3, 156.9, 154.5, 151.6, 149.2, 143.9, 142.8, 140.6, 138.7, 137.9, 137.6, 129.8, 129.5, 128.6, 127.5, 125.8, 124.4, 124.1, 117.8, 72.5, 69.9, 30.7 (2C), 30.1 (2C). LC–MS: *t*_R_ = 0.846 min (method 1, purity 100%); *m*/*z* = 418.2 [M + H]^+^ (anal. calcd for C_25_H_24_FN_3_O_2_: *m*/*z* = 417.2).

#### 2-[5-[6-[6-(Trifluoromethyl)­pyridin-3-yl]-1,5-naphthyridin-4-yl]­pyridin-2-yl]­propan-2-ol (**32**)

Synthesized from **10a** (100 mg, 0.32 mmol) and [6-(2-hydroxypropan-2-yl)­pyridin-3-yl]­boronic acid (117 mg, 0.65 mmol) according to Method A. Brown solid (23 mg, 17% yield); ^1^H NMR (400 MHz, DMSO-*d*_6_, δ): 9.54 (s, 1H), 9.12 (dd, *J* = 4.6, 1.6 Hz, 1H), 9.01 (d, *J* = 2.1 Hz, 1H), 8.81 (d, *J* = 8.3 Hz, 1H), 8.68 (dd, *J* = 8.9, 1.7 Hz, 1H), 8.61 (dd, *J* = 8.8, 1.7 Hz, 1H), 8.41 (dd, *J* = 8.2, 2.3 Hz, 1H), 8.11 (d, *J* = 8.2 Hz, 1H), 8.00 (dd, *J* = 4.6, 1.6 Hz, 1H), 7.91 (d, *J* = 8.2 Hz, 1H), 5.39 (d, *J* = 1.7 Hz, 1H), 1.55 (s, 6H). ^13^C NMR (151 MHz, DMSO): δ 168.6, 152.9, 152.1, 149.1, 149.0, 146.9, 144.0, 143.4, 140.6, 139.1, 138.9, 136.9, 136.6, 129.4, 124.7, 122.7, 121.0 (2C), 117.9, 72.5, 30.7 (2C). LC–MS: *t*_R_ = 2.526 min (method 2, purity 100%); *m*/*z* = 411.1 [M + H]^+^ (anal. calcd for C_22_H_17_F_3_N_4_O: *m*/*z* = 410.1).

#### 1-Methyl-5-[6-(3-methylsulfonylphenyl)-1,5-naphthyridin-4-yl]­pyridin-2-one (**33**)

Synthesized from **10b** (100 mg, 0.31 mmol) and *N*-Methyl-1*H*-pyridin-2-one-5-boronic acid, pinacol ester (111 mg, 0.47 mmol) according to Method A. Yellow solid (7 mg, 5% yield); ^1^H NMR (300 MHz, DMSO-*d*_6_, δ): 9.00 (d, *J* = 4.6 Hz, 1H), 8.83 (d, *J* = 2.3 Hz, 1H), 8.60 (d, *J* = 13.4 Hz, 4H), 8.17–8.03 (m, 2H), 7.88 (dd, *J* = 6.1, 4.3 Hz, 2H), 6.59 (d, *J* = 9.4 Hz, 1H), 3.63 (s, 3H). ^13^C NMR (151 MHz, DMSO-*d*_6_): δ 161.7, 154.5, 151.9, 143.9, 142.7, 142.5, 142.0, 140.8, 139.6, 139.4, 132.4, 130.6, 128.4, 126.1, 123.0, 122.3, 118.6, 113.8, 44.0, 37.5. LC–MS: *t*_R_ = 0.869 min (method 1, purity 100%); *m*/*z* = 392.1 [M + H]^+^ (anal. calcd for C_21_H_17_N_3_O_3_S: *m*/*z* = 391.1).

#### 1-Methyl-4-[6-(3-methylsulfonylphenyl)-1,5-naphthyridin-4-yl]­pyridin-2-one (**34**)

Synthesized from **10b** (100 mg, 0.31 mmol) and 1-methyl-4-(4,4,5,5-tetramethyl-1,3,2-dioxaborolan-2-yl)­pyridin-2-one (147 mg, 0.63 mmol) according to Method A. Yellow solid (28 mg, 22% yield); ^1^H NMR (300 MHz, DMSO-*d*_6_, δ): 9.09 (d, *J* = 4.4 Hz, 1H), 8.77 (d, *J* = 1.8 Hz, 1H), 8.60 (dd, *J* = 15.5, 6.7 Hz, 3H), 8.07 (d, *J* = 7.8 Hz, 1H), 7.94–7.74 (m, 3H), 6.88 (d, *J* = 1.8 Hz, 1H), 6.78 (dd, *J* = 7.0, 1.9 Hz, 1H), 3.55 (s, 3H), 3.34 (s, 3H). ^13^C NMR (151 MHz, DMSO-*d*_6_): δ 162.1, 155.0, 152.1, 148.3, 144.4, 143.6, 142.3, 140.4, 139.5, 139.3, 138.8, 132.5, 130.6, 128.5, 126.2, 124.6, 122.7, 120.6, 108.1, 43.8, 37.0. LC–MS: *t*_R_ = 0.869 min (method 1, purity 100%); *m*/*z* = 392.1 [M + H]^+^ (anal. calcd for C_21_H_17_N_3_O_3_S: *m*/*z* = 391.1).

#### 1-(2-Hydroxyethyl)-4-[6-(3-methylsulfonylphenyl)-1,5-naphthyridin-4-yl]­pyridin-2-one (**35**)

Synthesized from **10b** (50 mg, 0.16 mmol) with tris­(dibenzylideneacetone)­dipalladium(0) (14.36 mg, 0.02 mmol), and tricyclohexylphosphine (11 mg, 0.04 mmol) according to Method A. Brown solid (21 mg, 32% yield); ^1^H NMR (300 MHz, DMSO-*d*_6_): δ 9.10 (d, *J* = 4.4 Hz, 1H), 8.78 (t, *J* = 1.8 Hz, 1H), 8.68–8.53 (m, 3H), 8.07 (dt, *J* = 8.1, 1.2 Hz, 1H), 7.92 (d, *J* = 4.4 Hz, 1H), 7.85 (t, *J* = 7.8 Hz, 1H), 7.75 (d, *J* = 7.0 Hz, 1H), 6.89 (d, *J* = 1.9 Hz, 1H), 6.78 (dd, *J* = 7.0, 2.0 Hz, 1H), 4.95 (t, *J* = 5.3 Hz, 1H), 4.06 (td, *J* = 5.3, 2.2 Hz, 2H), 3.73 (q, *J* = 5.5 Hz, 2H), 3.33 (s, 3H). ^13^C NMR (151 MHz, DMSO): δ 161.8, 155.0, 152.2, 148.4, 144.5, 143.6, 142.4, 140.5, 139.5, 139.4, 139.2, 132.6, 130.7, 128.5, 126.4, 124.6, 122.8, 120.9, 107.9, 59.2, 51.7, 43.8. LC–MS: *t*_R_ = 0.810 min (method 1, purity 100%); *m*/*z* = 422.1 [M + H]^+^ (anal. calcd for C_22_H_18_N_3_O_4_S: *m*/*z* = 421.1).

#### [3-[6-(3-Methylsulfonylphenyl)-1,5-naphthyridin-4-yl]­phenyl]-(3-hydroxypyrrolidin-1-yl)­methanone (**36**)

Synthesized from **10b** (100 mg, 0.31 mmol) and [(3*R*)-3-hydroxypyrrolidin-1-yl]-[3-(4,4,5,5-tetramethyl-1,3,2-dioxaborolan-2-yl)­phenyl]­methanone (149 mg, 0.47 mmol) according to Method A. Yellow solid (23 mg, 15% yield); ^1^H NMR (600 MHz, DMSO-*d*_6_, δ): 9.09 (d, *J* = 4.4 Hz, 1H), 8.75–8.69 (m, 1H), 8.65 (d, *J* = 8.8 Hz, 1H), 8.64–8.54 (m, 2H), 8.16–8.06 (m, 1H), 8.07–8.01 (m, 2H), 7.94 (dd, *J* = 4.4, 3.1 Hz, 1H), 7.83 (tt, *J* = 7.8, 3.9 Hz, 1H), 7.77–7.64 (m, 2H), 4.92 (d, *J* = 3.2 Hz, 1H), 4.39–4.07 (m, 1H), 3.67–3.49 (m, 3H), 3.39 (dt, *J* = 12.7, 1.8 Hz, 1H), 3.34–3.25 (m, 3H), 1.95 (dtd, *J* = 13.4, 9.1, 4.4 Hz, 1H), 1.85 (tdd, *J* = 13.2, 8.8, 4.4 Hz, 1H). ^13^C NMR (151 MHz, DMSO): δ 168.2, 168.1, 154.6, 154.5, 151.7, 146.1, 146.0, 143.4, 141.8, 141.8, 140.5, 140.4, 139.2, 139.1, 138.9, 137.0, 136.8, 136.0, 132.3, 132.2, 132.2, 132.0, 130.7, 130.2, 129.6, 129.5, 128.1, 128.1, 128.0, 127.5, 125.8, 125.7, 124.7, 124.7, 122.2, 122.1, 69.4, 68.0, 57.2, 54.4, 47.1, 44.2, 43.4, 43.4, 40.0, 34.3, 32.2. LC–MS: *t*_R_ = 2.356 min (method 2, purity 100%); *m*/*z* = 474.1 [M + H]^+^ (anal. calcd for C_26_H_23_N_3_O_4_S: *m*/*z* = 473.1).

#### (3-Hydroxypyrrolidin-1-yl)-[4-[6-(3-methylsulfonylphenyl)-1,5-naphthyridin-4-yl]­phenyl]­methanone (**37**)

Synthesized from **10b** (80 mg, 0.25 mmol) and [4-[(3*R*)-3-hydroxypyrrolidine-1-carbonyl]­phenyl]­boronic acid (71 mg, 0.30 mmol) according to Method A using a mixture of 1-butanol/water (4:1, 5 mL) as solvent. Brown powder (44 mg, 37% yield); ^1^H NMR (300 MHz, DMSO-*d*_6_, δ): 9.11 (d, *J* = 4.5 Hz, 1H), 8.82 (s, 1H), 8.71–8.54 (m, 3H), 8.12–8.01 (m, 3H), 7.95 (d, *J* = 4.5 Hz, 1H), 7.85 (t, *J* = 7.8 Hz, 1H), 7.73 (d, *J* = 7.9 Hz, 2H), 7.51 (s, 1H), 4.99 (dd, *J* = 29.7, 3.5 Hz, 1H), 4.34 (d, *J* = 25.3 Hz, 1H), 3.75–3.56 (m, 3H), 1.94 (d, *J* = 39.6 Hz, 2H). ^13^C NMR (101 MHz, DMSO-*d*_6_): δ 168.1, 154.2, 151.5, 145.8, 143.3, 141.8, 140.2, 138.8, 137.0, 131.8, 130.5, 130.1, 127.9, 126.8, 125.7, 124.4, 121.8, 69.3, 68.0, 57.0, 54.3, 46.9, 44.0, 43.3, 39.5, 34.3, 32.1. LC–MS: *t*_R_ = 0.882 min (method 1, purity 100%); *m*/*z* = 474.1 [M + H]^+^ (anal. calcd for C_26_H_23_N_3_O_4_S: *m*/*z* = 473.1).

#### [(3*S*)-3-Aminopyrrolidin-1-yl]-[4-[6-(3-methylsulfonylphenyl)-1,5-naphthyridin-4-yl]­phenyl]­methanone (**38**)

A mixture of *tert*-butyl (*S*)-(1-(4-(6-(3-(methylsulfonyl)­phenyl)-1,5-naphthyridin-4-yl)­benzoyl)­pyrrolidin-3-yl)­carbamate (167 mg, 0.29 mmol) and hydrogen chloride solution (1.5 mL, 5.83 mmol) in 1,4-dioxane in 5 mL DCM was stirred at 23 °C for 16 h. The solvent was removed in vacuo. The residue was diluted with 15 mL MeOH/DCM (1:9) and neutralized by stirring with Amberlyst 21 resin for 1 h. The resin was filtered and recrystallized from hot DCM/MeOH solution to give **38**. Beige solid (12 mg, 8% yield); ^1^H NMR (300 MHz, MeOH-*d*_4_, δ): 9.03 (d, *J* = 4.4 Hz, 1H), 8.92 (s, 1H), 8.62–8.46 (m, 3H), 8.05 (d, *J* = 7.6 Hz, 3H), 7.91 (d, *J* = 4.2 Hz, 1H), 7.80 (t, *J* = 7.9 Hz, 3H), 4.10–3.65 (m, 4H), 3.18 (d, *J* = 16.1 Hz, 3H), 2.46 (s, 1H), 2.15 (s, 1H). ^13^C NMR (151 MHz, DMSO): δ 168.6, 168.5, 154.9, 154.8, 152.2, 146.4, 146.3, 143.9, 142.3, 140.8, 139.5 (2C), 139.4, 138.0, 137.3, 137.0, 132.5, 131.3, 131.2, 130.8, 130.8, 128.6, 127.4, 127.3, 126.3, 125.1, 122.5, 122.4, 53.4, 50.7, 50.5, 49.1, 47.2, 44.3, 43.9 (2C), 30.9, 29.1. LC–MS: *t*_R_ = 0.725 min (method 1, purity 100%); *m*/*z* = 473.2 [M + H]^+^ (anal. calcd for C_26_H_24_N_4_O_3_S: *m*/*z* = 472.1).

### DMPK

All protocols for in vitro DMPK studies, in vivo DMPK and NSG efficacy studies are available in the Supporting Information document. Animal studies were conducted following guidelines and policies as stipulated in the UCT Research Ethics Code for Use of Animals in Research and Teaching, after review and approval of the experimental protocol by the UCT Senate Animal Ethics Committee (protocol FHS-AEC 022_004 (Mouse PK)) and FHS-AEC 021_015 (In vivo NSG mouse model for malaria).

## Supplementary Material











## Notes

Synthesis of intermediates, analytical spectra for the compounds tested in vivo, In vitro biological assays, in vitro ADME assays, mouse pharmacokinetic studies, and in vivo efficacy (PDF)
Table S1 (XLSX)
In vitro data of all final compounds (mean IC_50_ values ± standard deviation (SD)) (CSV)
Molecular formula strings (CSV)
PDB file of the in silico docking image (PDB)

## Abbreviations

ABS: asexual blood stage
ADME: absorption, distribution, metabolism, and excretion
ADP: adenosine diphosphate
ATP: adenosine triphosphate
AUC: area under the curve
Cs_2_CO_3_: Cesium carbonate
DCM: dichloromethane
DMAP: 4-(dimethylamino)­pyridine
DMF: *N*,*N*-dimethylformamide
DMPK: drug metabolism and pharmacokinetics
HCl: hydrochloric acid
K_2_CO_3_: potassium carbonate
K_3_PO_4_: tripotassium phosphate
MAP4K4: mitogen-activated protein kinase kinase kinase kinase 4
MeOH: methanol
MINK1: misshapen-like kinase 1
NMR: nuclear magnetic resonance
PdCl_2_(dppf): 1,1′-bis­(diphenylphosphino)­ferrocene]­dichloropalladium­(II), complex with dichloromethane
PdCl_2_(PPh_3_)_2_: bis­(triphenylphosphine)­palladium­(II) chloride
PI4K: phosphatidylinositol 4-kinase IIIβ
POCl_3_: phosphorus­(V) oxychloride
SAR: structure–activity relationship
TPSA: topological polar surface area
