# Supplementary material for: Optimization of 2,8-Diaryl-1,5-naphthyridines as Plasmodium falciparum Phosphatidylinositol 4‑Kinase Inhibitors with Improved ADME Profiles and In Vivo Efficacy
Source: J Med Chem. 2025 Oct 7;68(20):21878–91. doi: 10.1021/acs.jmedchem.5c02248 (PMC12557380; doi:10.1021/acs.jmedchem.5c02248)
Supplement: Supplementary file 1 [file jm5c02248_si_001.pdf]

SUPPORTING INFORMATION

Optimization of 2,8-Diaryl-1,5-naphthyridines as *Plasmodium falciparum* phosphatidylinositol 4-kinase inhibitors with improved ADME profiles and *in vivo* efficacy

Godwin A. Dziwornu,[a] *Donald Seanego,*[a] *Stephen Fienberg,*[a] *Venkata S. Sypu,*[a] *Nicolaas Salomane,*[b] *Liezl Krugmann,*[b] *Dale Taylor,*[b] *Keabetswe Masike,*[b] *Mathew Njoroge,*[b] *Nonlawat Boonyalai,*[c,] *Marcus C. S. Lee,*[c] *Luiz C. Godoy,*[d] *Charisse Flerida Pasaje,*[d] *Jacquin C. Niles*[d] *Gregory S. Basarab,*[a] *Lauren B. Coulson,*[b] *Sandeep R. Ghorpade,*[a] *and Kelly Chibale*[a, e*]

[a]Holistic Drug Discovery and Development (H3D) Centre, Department of Chemistry, University of Cape Town, Rondebosch 7701, South Africa.

[b]Holistic Drug Discovery and Development (H3D) Centre, Institute of Infectious Disease and Molecular Medicine, University of Cape Town, Observatory, Cape Town 7925, South Africa.

[c]Biological Chemistry and Drug Discovery, Wellcome Centre for Anti-Infectives Research, University of Dundee, Dundee DD1 5EH, U.K.

[d]Department of Biological Engineering, Massachusetts Institute of Technology; Cambridge, MA 02139, USA.

[e]South African Medical Research Council Drug Discovery and Development Research Unit, Department of Chemistry and Institute of Infectious Disease and Molecular Medicine, University of Cape Town, Rondebosch 7701, South Africa.

*Corresponding author: Kelly Chibale (kelly.chibale@uct.ac.za)

Contents

1. Additional experimental data S3
2. Analytical data of representative target compounds S9
3. *In vitro* biological assays S30
4. *In vitro* ADME assays S32
5. *In vivo* studies S33
6. References S36

1. Additional Experimental Data

General synthesis methods

Method A: The appropriate intermediate (1 eq), boronic acid or (1.2 eq), potassium phosphate tribasic (or cesium carbonate) (1.7 eq.) and [1,1'-Bis(diphenylphosphino)-ferrocene] dichloropalladium (II) (0.05 eq.) were placed in a reaction vessel and purged with nitrogen gas. To this mixture was added degassed 1,4-dioxane and degassed distilled water (9:1, 5 mL). The reaction mixture was stirred at 100-120 ˚C for 0.5-18 h (or irradiated under microwave conditions for 30 min at 120 ˚C, 200 W (dynamic mode)). When complete, the mixture was cooled to room temperature, filtered through a pad of celite and concentrated under reduced pressure. The crude product was purified by either normal or reverse phase chromatography.

Method B: To a solution of intermediate IV (1 eq) in DMF (5 mL) was added the relevant boronic acid/ester (1.2 eq), and Bis(triphenylphosphine)palladium (II) dichloride (0.05 eq), followed by potassium acetate or potassium carbonate (dissolved in water (10% by volume)). The mixture was degassed by bubbling nitrogen through it. The reaction was heated at 90-110 ˚C until completion (0.5-2 h). The mixture was cooled to ambient temperature, filtered through a pad of celite and concentrated under reduced pressure. The crude product was purified by either normal or reverse phase chromatography.

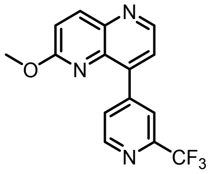

2-methoxy-8-(2-(trifluoromethyl)pyridin-4-yl)-1,5-naphthyridine (5a)

Synthesized from **4** (3000 mg, 15.41 mmol) and 2-(Trifluoromethyl)pyridine-4-boronic acid (3530 mg, 18.5 mmol) as previously described.[1] Beige solid (2500 mg, 43% yield). LC-MS: t_R = 1.145 min (method 1, purity 82%); *m/z* = 306.1 $[M+H]^+$ (anal. calcd for $C_{15}H_{10}F_3N_3O$: *m/z* = 305.1).

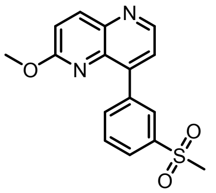

2-methoxy-8-(3-(methylsulfonyl)phenyl)-1,5-naphthyridine (5b)

Synthesized from **4** (1000 mg, 5.14 mmol) and 3-methylsulfonylphenylboronic acid (1130 mg, 5.65 mmol) as previously described.[1] Beige solid (912 mg, 56% yield). LC-MS: t_R = 2.367 min (method 2, purity 100%); *m/z* = 315.0 $[M+H]^+$ (anal. calcd for $C_{16}H_{14}N_2O_3S$: *m/z* = 314.0).

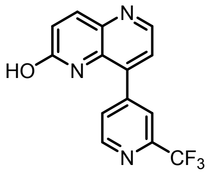

8-(2-(trifluoromethyl)pyridin-4-yl)-1,5-naphthyridin-2-ol (6a)

Synthesized from **5a** (1705 mg, 5.59 mmol) as previously described.[1] Beige solid (1159 mg, 71% yield). LC-MS: t_R = 0.856 min (method 1, purity 100%); *m/z* = 292.1 [M+H]$^+$ (anal. calcd for $C_{14}H_8F_3N_3O$: *m/z* = 291.1).

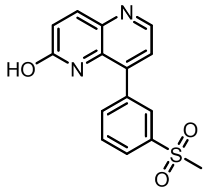

8-(3-(methylsulfonyl)phenyl)-1,5-naphthyridin-2-ol (6b)

Synthesized from **5b** (1000 mg, 3.18 mmol) as previously described.[1] Beige solid (720 mg, 75% yield). LC-MS: t_R = 0.679 min (method 1, purity 100%); *m/z* = 301.1 [M+H]$^+$ (anal. calcd for $C_{15}H_{12}N_2O_3S$: *m/z* = 300.1).

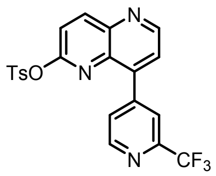

[8-[2-(trifluoromethyl)pyridin-4-yl]-1,5-naphthyridin-2-yl] 4-methylbenzenesulfonate (7a)

Synthesized from **6a** (1159.mg, 3.98 mmol) as previously described.[1] White solid (1581 mg, 89% yield); ^{1}H NMR (600 MHz, DMSO) δ 9.13 (d, *J* = 4.4 Hz, 1H), 8.91 (d, *J* = 5.0 Hz, 1H), 8.69 (d, *J* = 8.9 Hz, 1H), 8.17 (s, 1H), 8.00 (d, *J* = 4.4 Hz, 1H), 7.87 (dd, *J* = 5.0, 1.6 Hz, 1H), 7.69 (d, *J* = 9.0 Hz, 1H), 7.63 – 7.54 (m, 2H), 7.17 (d, *J* = 8.0 Hz, 2H), 2.32 (s, 3H). LC-MS: t_R = 1.271 min (method 1, purity 100%); *m/z* = 446.1 [M+H]$^+$ (anal. calcd for $C_{21}H_{14}F_3N_3O_3S$: *m/z* = 445.1).

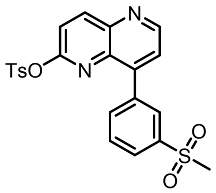

[8-(3-methylsulfonylphenyl)-1,5-naphthyridin-2-yl] 4-methylbenzenesulfonate (7b)

Synthesized from **6b** (2170.mg, 7.23mmol) as previously described.[1] Yellow solid (2480 mg, 75% yield); ^{1}H NMR (300 MHz, DMSO-d_6) δ 9.09 (d, J = 4.5 Hz, 1H), 8.67 (d, J = 9.0 Hz, 1H), 8.18 (d, J = 1.7 Hz, 1H), 8.13 (d, J = 7.8 Hz, 1H), 7.91 (dd, J = 8.4, 3.0 Hz, 2H), 7.80 (t, J = 7.8 Hz, 1H), 7.63 (dd, J = 14.3, 8.6 Hz, 3H), 7.21 (d, J = 8.1 Hz, 2H), 3.33 (s, 3H), 2.33 (s, 3H). LC-MS: t_R = 2.483 min (method 2, purity 100%); m/z = 455.0 [M+H]$^+$ (anal. calcd for $C_{22}H_{18}N_2O_5S_2$: m/z = 454.1).

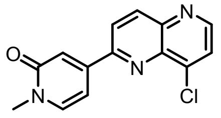

4-(8-chloro-1,5-naphthyridin-2-yl)-1-methylpyridin-2(1H)-one (13a)

Synthesized from **9** (500 mg, 1.49 mmol), (1-methyl-2-oxopyridin-4-yl)boronic acid (274 mg, 1.79 mmol) according to Method A at 25 °C for 2 h. Brown solid (310 mg, 73% yield); LC-MS: t_R = 2.224 min (method 2, purity 96%); m/z = 272.1 [M+H]$^+$ (anal. calcd for $C_{14}H_{10}ClN_3O$: m/z = 271.1).

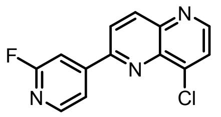

8-chloro-2-(2-fluoropyridin-4-yl)-1,5-naphthyridine (16a)

Synthesized from **9** (785 mg, 2.34 mmol) and 2-fluoropyridine-4-boronic acid (396 mg, 2.81 mmol) according to Method A. Yellow solid ((535 mg, 80% yield); LC-MS: t_R = 0.991 min (method 1, purity 91%); m/z = 260.1 [M+H]$^+$ (anal. calcd for $C_{13}H_7ClFN_3$: m/z = 259.1).

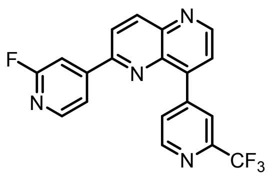

2-(2-fluoropyridin-4-yl)-8-(2-(trifluoromethyl)pyridin-4-yl)-1,5-naphthyridine (16b)

Synthesized from **16a** (187 mg, 0.66 mmol) and 2-(Trifluoromethyl)pyridine-4-boronic acid pinacol ester (214 mg, 0.79 mmol) according to Method B at 70 °C for 2 h. Yellow solid ((144 mg, 52% yield); LC-MS: t_R = 1.088 min (method 1, purity 89%); m/z = 371.1 [M+H]$^+$ (anal. calcd for $C_{19}H_{10}F_4N_4$: m/z = 370.1).

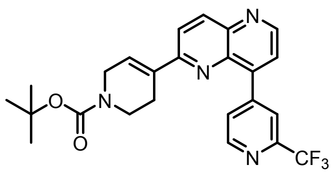

tert-butyl 4-(8-(2-(trifluoromethyl)pyridin-4-yl)-1,5-naphthyridin-2-yl)-3,6-dihydropyridine-1(2H)-carboxylate (17a)

Synthesized from **7a** (300.mg, 0.67 mmol) and 3,6-Dihydro-2H-pyridine-1-N-Boc-4-boronic acid, pinacol ester (250 mg, 0.81 mmol) according to Method A. Beige solid (297 mg, 96% yield); ^{1}H NMR (400 MHz, DMSO) δ 9.08 (d, *J* = 4.4 Hz, 1H), 8.97 (d, *J* = 5.0 Hz, 1H), 8.55 – 8.42 (m, 2H), 8.26 – 8.13 (m, 2H), 8.04 (d, *J* = 4.5 Hz, 1H), 7.03 – 6.87 (m, 1H), 4.12 (d, *J* = 3.7 Hz, 2H), 3.52 (t, *J* = 5.7 Hz, 2H), 2.65 – 2.57 (m, 2H), 1.43 (s, 9H). LC-MS: t_R = 1.423 min (method 1, purity 100%); *m/z* = 457.2 $[M+H]^+$ (anal. calcd for $C_{24}H_{23}F_3N_4O_2$: *m/z* = 456.2).

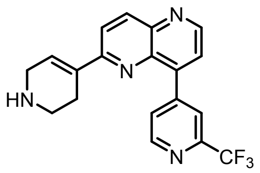

2-(1,2,3,6-tetrahydropyridin-4-yl)-8-(2-(trifluoromethyl)pyridin-4-yl)-1,5-naphthyridine (17b)

To a solution of **17a** (455 mg, 1.00 mmol) in DCM (5 mL) was added Hydrogen chloride solution (10500 mg, 287.99 mmol). The reaction mixture was stirred at 25 °C for 16 h. Purification was achieved by normal phase column chromatography eluting a gradient of ethyl acetate and hexane to give **17b**. Brown solid (320 mg, 89% yield); LC-MS: t_R = 2.124 min (method 2, purity 99%); *m/z* = 357.1 $[M+H]^+$ (anal. calcd for $C_{19}H_{15}F_3N_4$: *m/z* = 356.1).

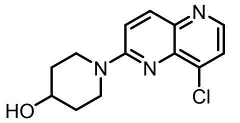

1-(8-chloro-1,5-naphthyridin-2-yl)piperidin-4-ol (21a)

Synthesized from **9** (400 mg, 1.19 mmol), 4-Hydroxypiperidine (145 mg, 1.43 mmol) and Cesium carbonate (973 mg, 2.99 mmol) in DMF (3 mL) at 90 °C for 18 h. Purification was achieved by normal phase chromatography eluting a gradient of methanol and DCM. Yellow solid (180 mg, 53% yield); ^{1}H NMR (300 MHz, DMSO-d_6) δ 8.43 (d, *J* = 4.7 Hz, 1H), 8.05 (d, *J* = 9.4 Hz, 1H), 7.71 (d, *J* = 4.7 Hz, 1H), 7.55 (d, *J* = 9.5 Hz, 1H), 4.74 (d, *J* = 4.3 Hz, 1H), 4.31 – 4.19 (m, 2H), 3.84 – 3.73 (m, 1H), 3.45 – 3.33 (m, 2H), 1.91 – 1.79 (m, 2H), 1.50 – 1.34 (m, 2H). LC-MS: t_R = 0.755 min (method 1, purity 93%); *m/z* = 264.1 $[M+H]^+$ (anal. calcd for $C_{13}H_{14}ClN_3O$: *m/z* = 263.1).

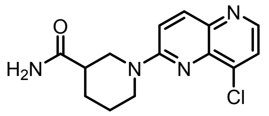

1-(8-chloro-1,5-naphthyridin-2-yl)piperidine-3-carboxamide (22a)

Synthesized from **9** (150 mg, 0.45 mmol) and Piperidine-3-carboxamide (69 mg, 0.54 mmol) as described for **21a** above. Beige solid (75 mg, 57% yield); ^{1}H NMR (300 MHz, DMSO) δ 8.44 (d, *J* = 4.8 Hz, 1H), 8.06 (d, *J* = 9.4 Hz, 1H), 7.72 (d, *J* = 4.7 Hz, 1H), 7.58 (d, *J* = 9.5 Hz, 1H), 7.37 (s, 1H), 6.87 (s, 1H), 4.74 – 4.41 (m, 2H), 3.24 – 2.94 (m, 2H), 2.46 – 2.28 (m, 1H), 2.02 – 1.86 (m, 1H), 1.86 – 1.60 (m, 2H), 1.59 – 1.38 (m, 1H). LC-MS: t_R = 0.764 min (method 1, purity 100%); *m/z* = 291.1 $[M+H]^+$ (anal. calcd for $C_{14}H_{15}ClN_4O$: *m/z* = 290.1).

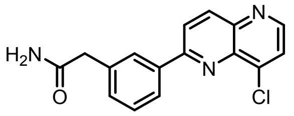

2-(3-(8-chloro-1,5-naphthyridin-2-yl)phenyl)acetamide (26a)

Synthesized from **9** (250 mg, 0.75 mmol) and 3-(2-Acetamidyl) phenylboronic acid pinacol ester (234 mg, 0.89 mmol) according to Method B at 40 °C for 2 h. White solid (243 mg, 94%). LC-MS: t_R = 0.838 min (method 1, purity 94%); *m/z* = 298.1 $[M+H]^+$ (anal. calcd for $C_{16}H_{12}ClN_3O$: *m/z* = 297.1).

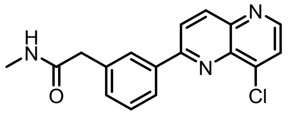

2-(3-(8-chloro-1,5-naphthyridin-2-yl)phenyl)-N-methylacetamide (27a)

Synthesized from **9** (250 mg, 0.75 mmol) and *N*-methyl-2-[3-(4,4,5,5-tetramethyl-1,3,2-dioxaborolan-2-yl)phenyl]acetamide (265 mg, 0.9 mmol) according to Method B. White solid (120 mg, 51%). LC-MS: t_R = 0.944 min (method 1, purity 100%); *m/z* = 312.1 $[M+H]^+$ (anal. calcd for $C_{17}H_{14}ClN_3O$: *m/z* = 311.1).

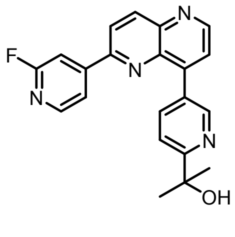

2-(5-(6-(2-fluoropyridin-4-yl)-1,5-naphthyridin-4-yl)pyridin-2-yl)propan-2-ol (28a)

Synthesized from **16a** (160 mg, 0.62 mmol) and [6-(2-hydroxypropan-2-yl)pyridin-3-yl]boronic acid (133 mg, 0.74 mmol) according to Method B. White solid (75 mg, 33%); LC-MS: t_R = 0.799 min (method 1, purity 100%); *m/z* = 361.1 $[M+H]^+$ (anal. calcd for $C_{21}H_{17}FN_4O$: *m/z* = 360.1).

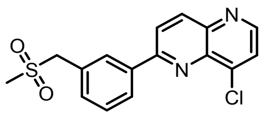

8-chloro-2-(3-((methylsulfonyl)methyl)phenyl)-1,5-naphthyridine (29a)

Synthesized from **9** (250 mg, 0.75 mmol) and 4,4,5,5-tetramethyl-2-[3-(methylsulfonylmethyl)phenyl]-1,3,2-dioxaborolane (265 mg, 0.89 mmol) according to Method B. White solid (132 mg, 52%). LC-MS: t_R = 0.910 min (method 1, purity 100%); *m/z* = 333.1 $[M+H]^+$ (anal. calcd for $C_{16}H_{13}ClN_2O_2S$: *m/z* = 332.1).

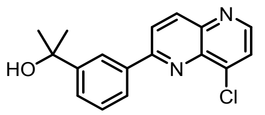

2-(3-(8-chloro-1,5-naphthyridin-2-yl)phenyl)propan-2-ol (30a)

Synthesized from **9** (400 mg, 1.19 mmo) 3-(2-hydroxypropan-2-yl)phenylboronic acid pinacol ester (376 mg, 1.43 mmol) according to Method B. White solid (200 mg, 56%). LC-MS: t_R = 2.722 min (method 2, purity 100%); *m/z* = 299.1 $[M+H]^+$ (anal. calcd for $C_{17}H_{15}ClN_2O$: *m/z* = 298.1).

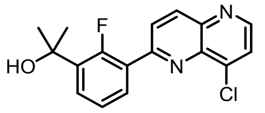

2-(3-(8-chloro-1,5-naphthyridin-2-yl)-2-fluorophenyl)propan-2-ol (31a)

Synthesized from **9** (468 mg, 1.40 mmol) 2-[2-fluoro-3-(4,4,5,5-tetramethyl-1,3,2-dioxaborolan-2-yl)phenyl]propan-2-ol (467 mg, 1.68 mmol) according to Method B. White solid (390 mg, 88%). LC-MS: t_R = 1.022 min (method 1, purity 100%); *m/z* = 317.1 $[M+H]^+$ (anal. calcd for $C_{17}H_{14}ClFN_2O$: *m/z* = 316.1).

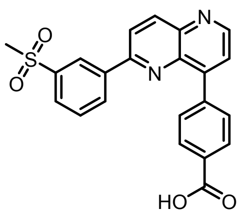

4-(6-(3-(methylsulfonyl)phenyl)-1,5-naphthyridin-4-yl)benzoic acid (38a)

Synthesized from intermediate **10b** (200 mg, 0.63 mmol) and 4-Carboxyphenylboronic acid (156 mg, 0.94 mmol) according to Method A. Brown solid (175 mg, 63% yield); LC-MS: t_R = 2.310 min (method 1, purity 92%); m/z = 405.1 $[M+H]^+$ (anal. calcd for $C_{22}H_{16}N_2O_4S$: m/z = 404.1).

2. Analytical data of representative target compounds

*2.1 Analytical spectra for compound **19**.*

HPLC_UV and MS spectra of **19**

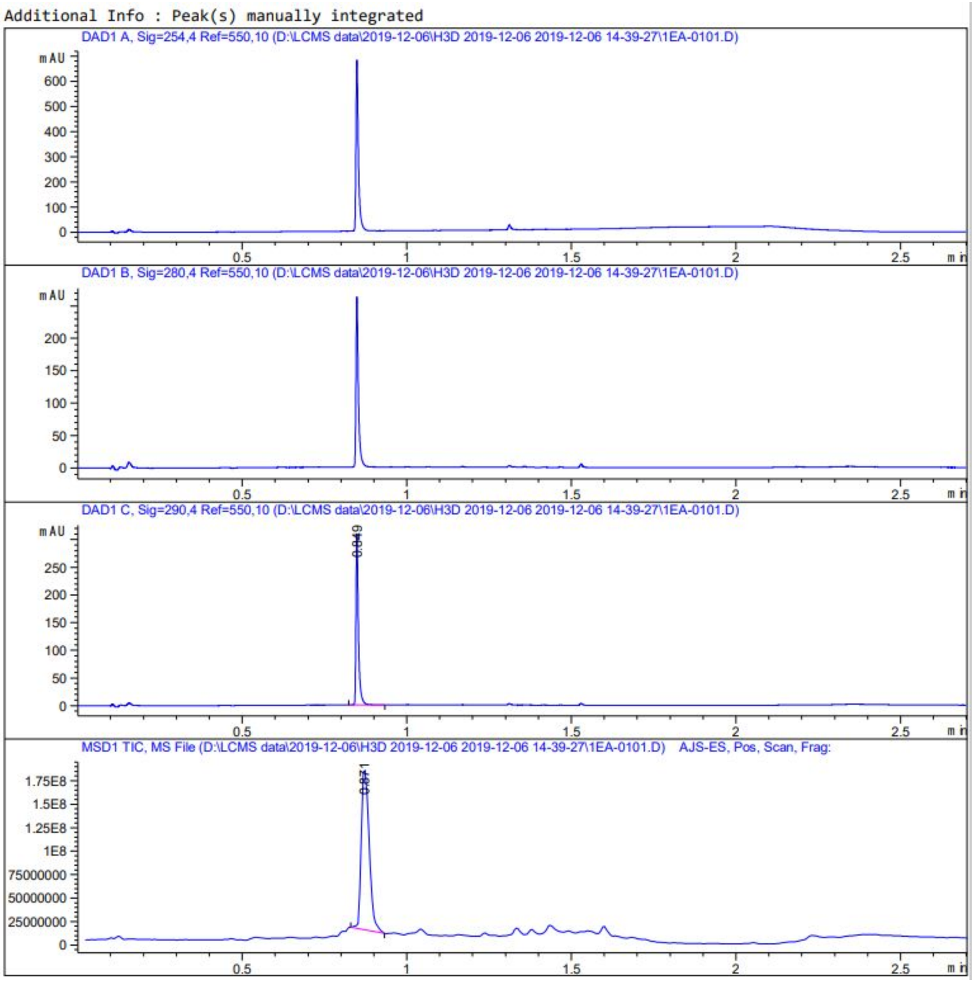

Signal 3: DAD1 C, Sig=290,4 Ref=550,10

Peak #	RetTime [min]	Type	Width [min]	Area [mAU*s]	Height [mAU]	Area %
1	0.849	BB	8.50e-3	165.60667	297.02542	100.0000
Totals :				165.60667	297.02542	

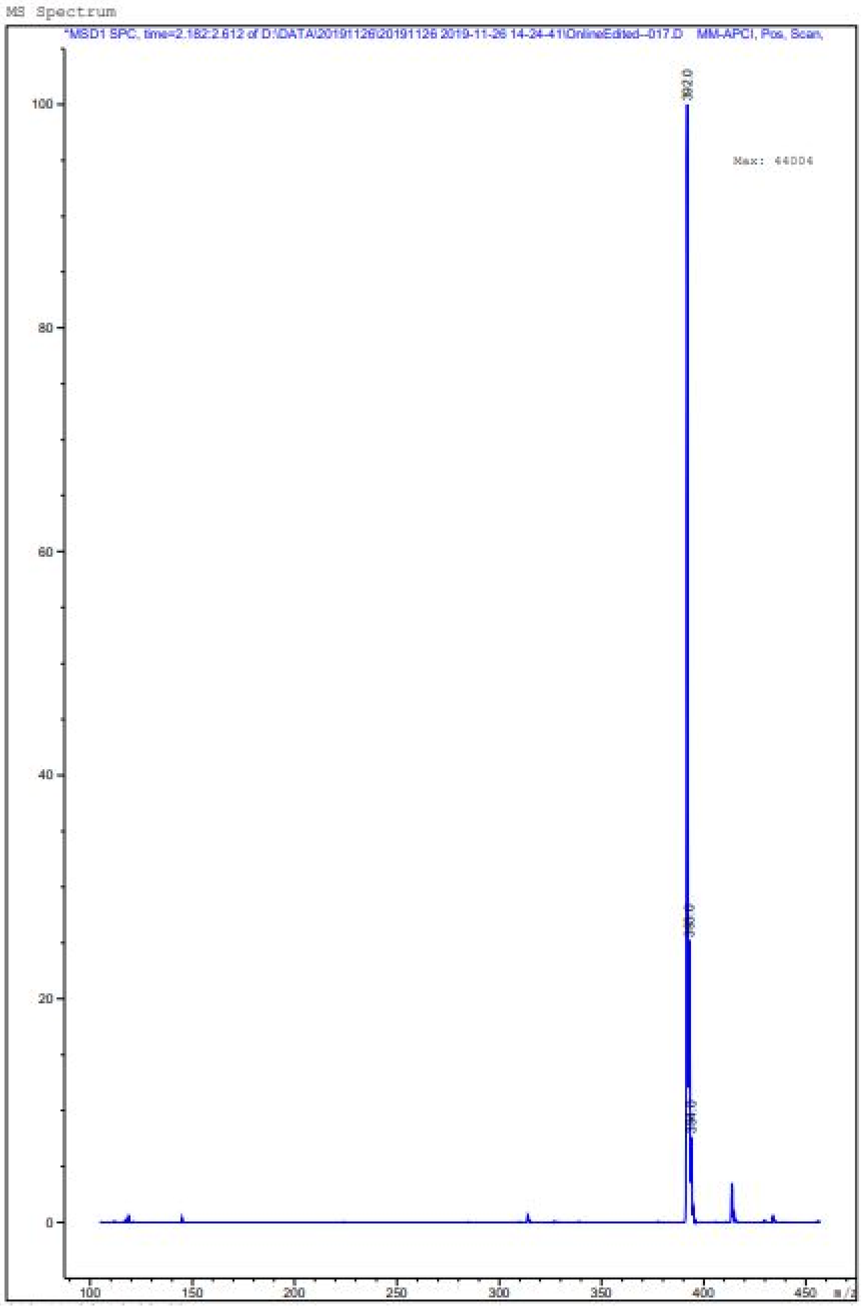
MS Spectrum
*MSD1 SPC, time=2.182:2.612 of D:\DATA\20191126\20191126 2019-11-26 14-24-41\OnlineEdited--017.D MM-APCI, Pos, Scan,
Max: 44004
392.0
0
20
40
60
80
100
100
150
200
250
300
350
400
450
m/z

^{1}H NMR spectrum of **21**

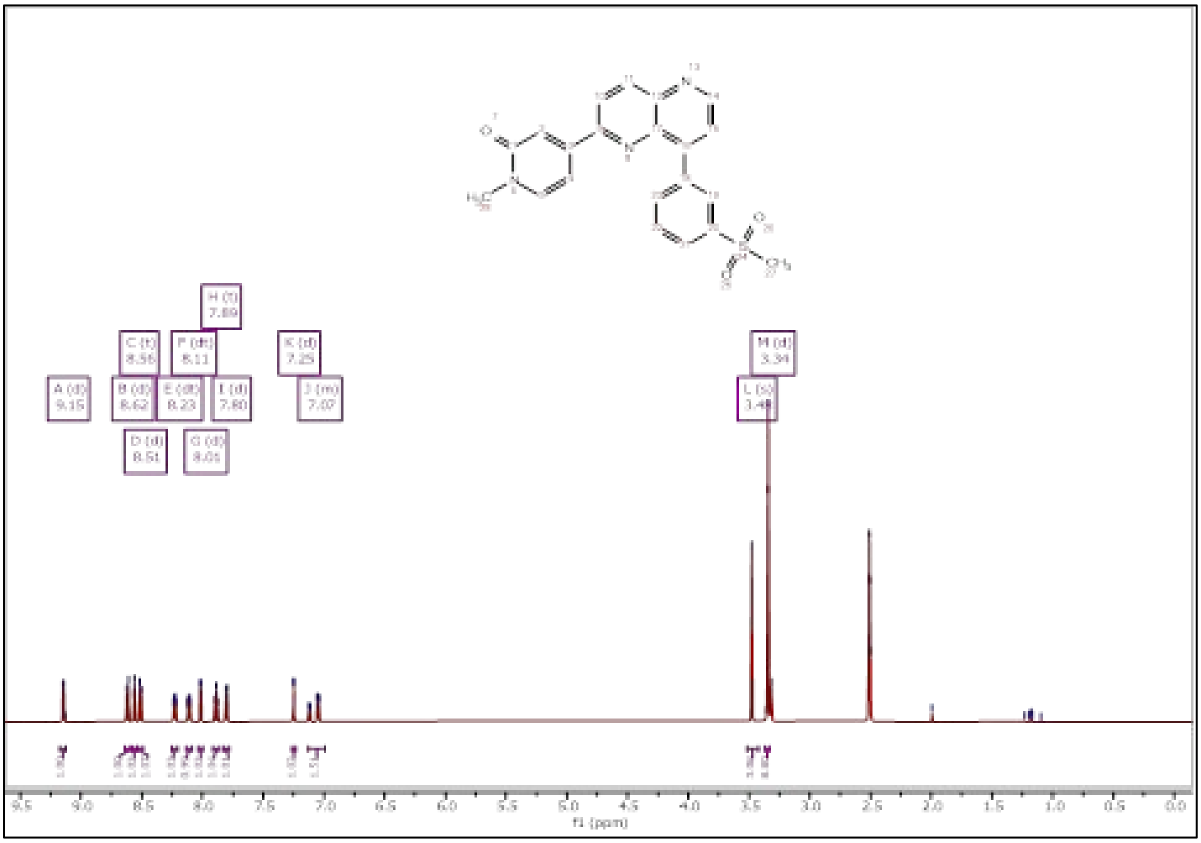

^{13}C NMR spectrum of **19**

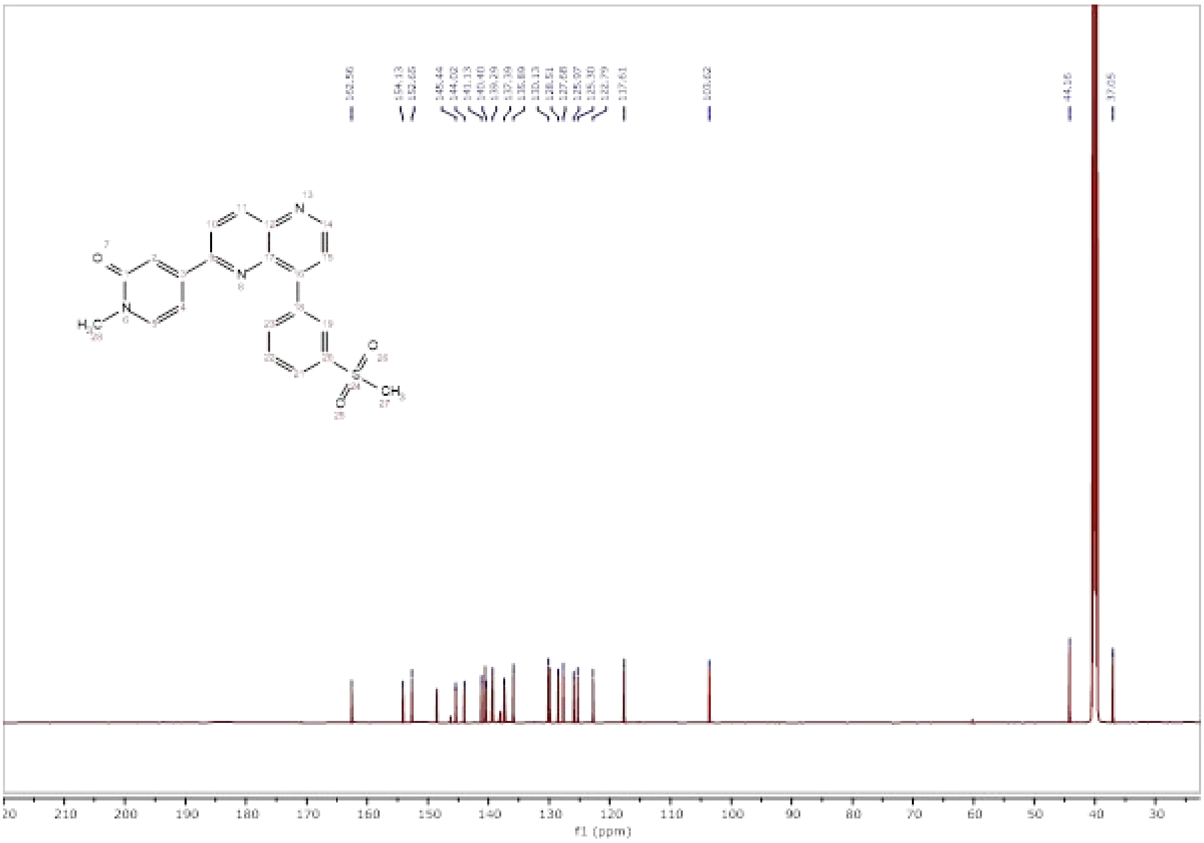

*2.1 Analytical spectra for compound **21**.*

HPLC_UV and MS spectra of **21**

Additional Info : Peak(s) manually integrated

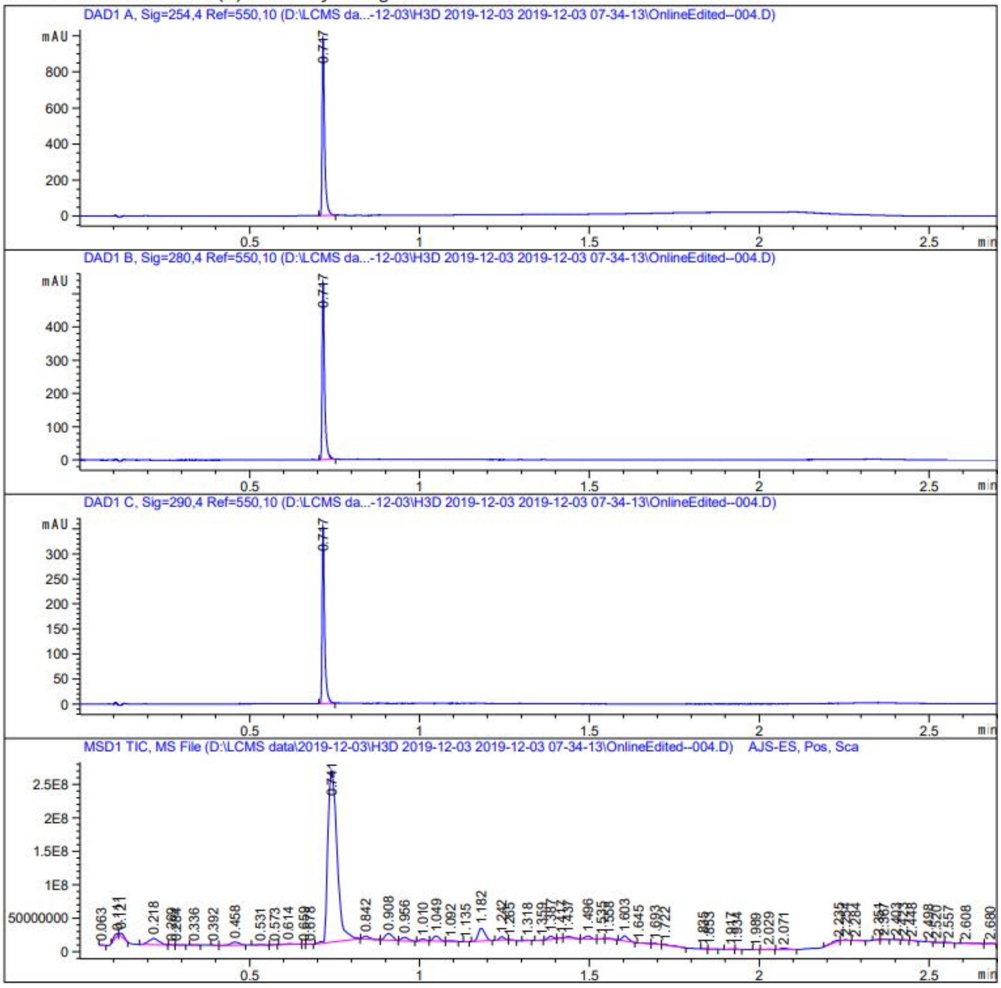

Signal 1: DAD1 A, Sig=254,4 Ref=550,10

Peak #	RetTime [min]	Type	Width [min]	Area [mAU*s]	Height [mAU]	Area %
1	0.717	BB	7.94e-3	496.19510	975.00775	100.0000
Totals :				496.19510	975.00775	

Signal 2: DAD1 B, Sig=280,4 Ref=550,10

Peak #	RetTime [min]	Type	Width [min]	Area [mAU*s]	Height [mAU]	Area %
1	0.717	BB	7.93e-3	268.51974	528.13324	100.0000
Totals :				268.51974	528.13324	

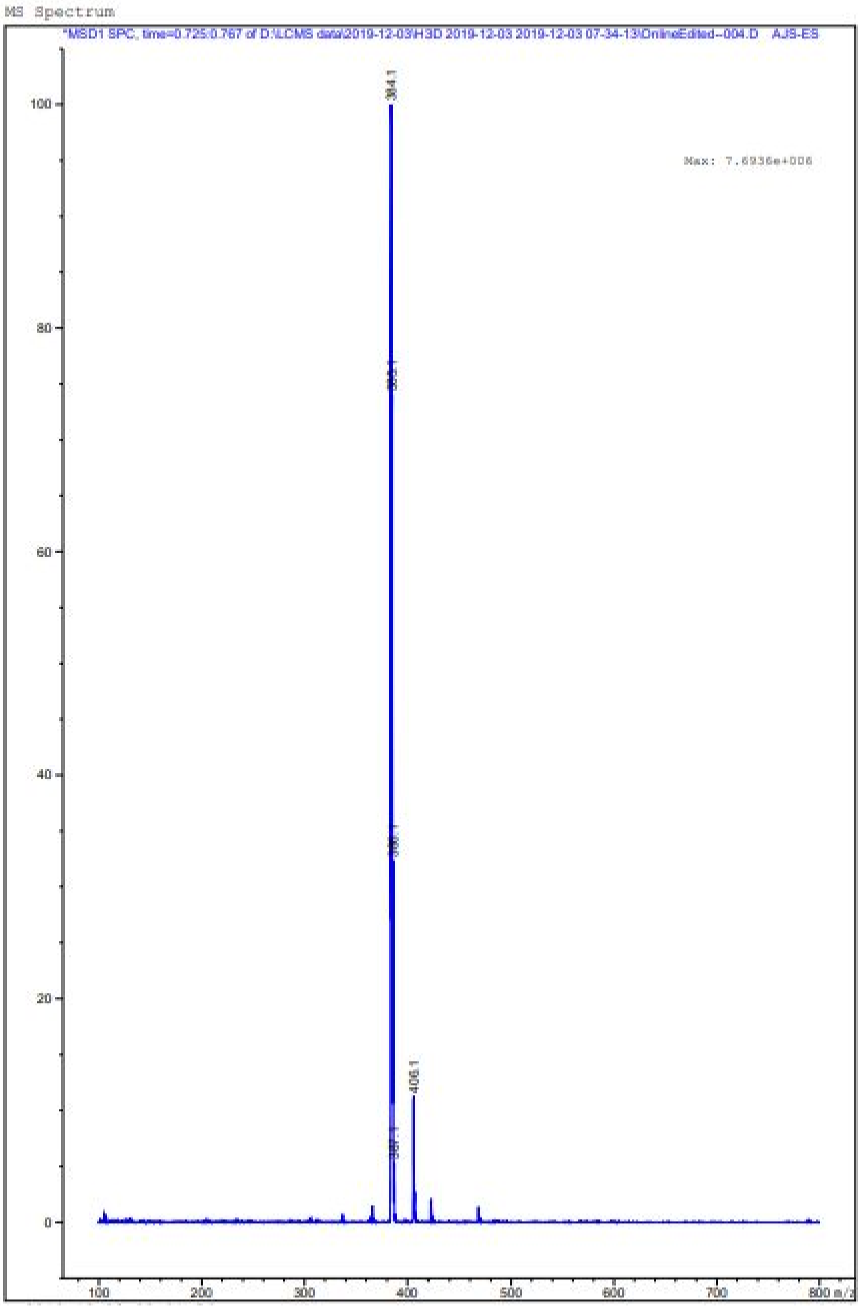
MS Spectrum
*MSD1 SPC, time=0.725:0.767 of D:\LCMS data\2019-12-03\H3D 2019-12-03 2019-12-03 07-34-13\OnlineEdited--004.D AJS-ES
Max: 7.6936e+006
384.1
406.1
100
80
60
40
20
0
100
200
300
400
500
600
700
800 m/z

^{1}H NMR spectrum of **21**

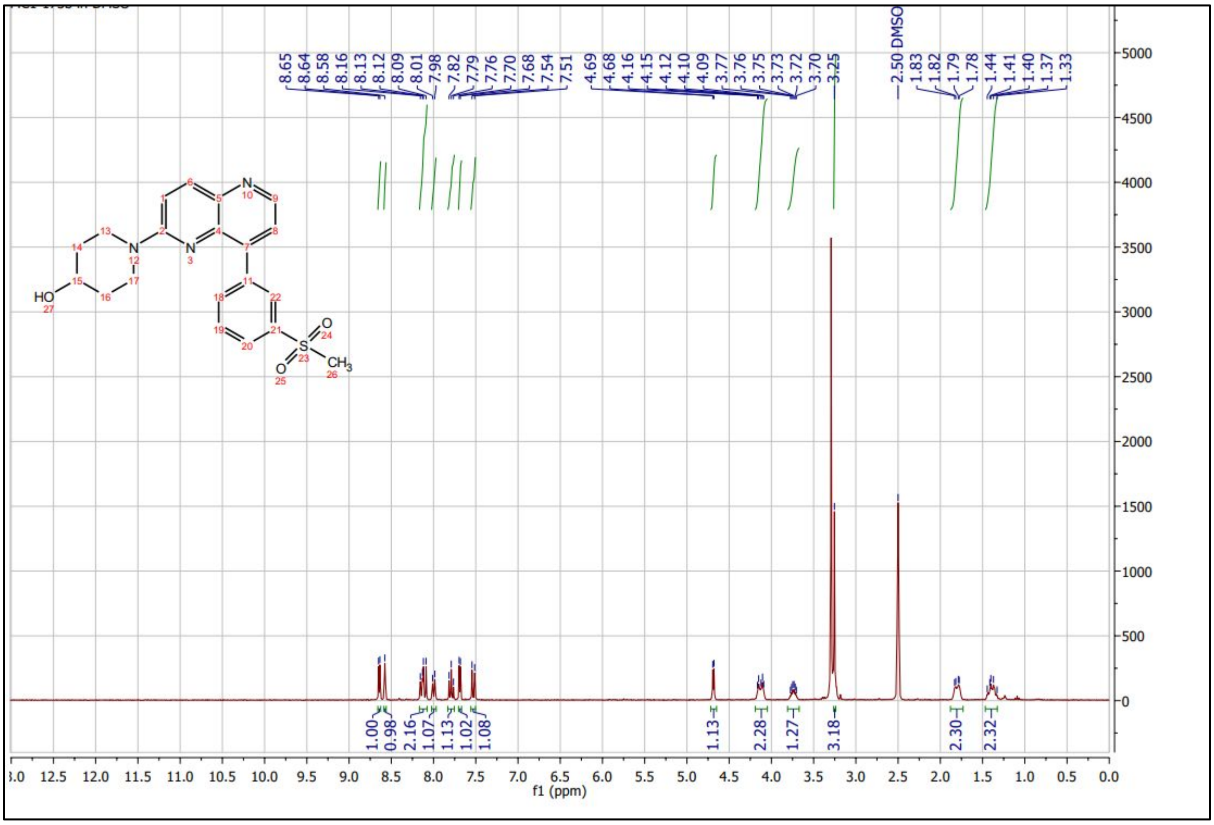

^{13}C NMR spectrum of **21**

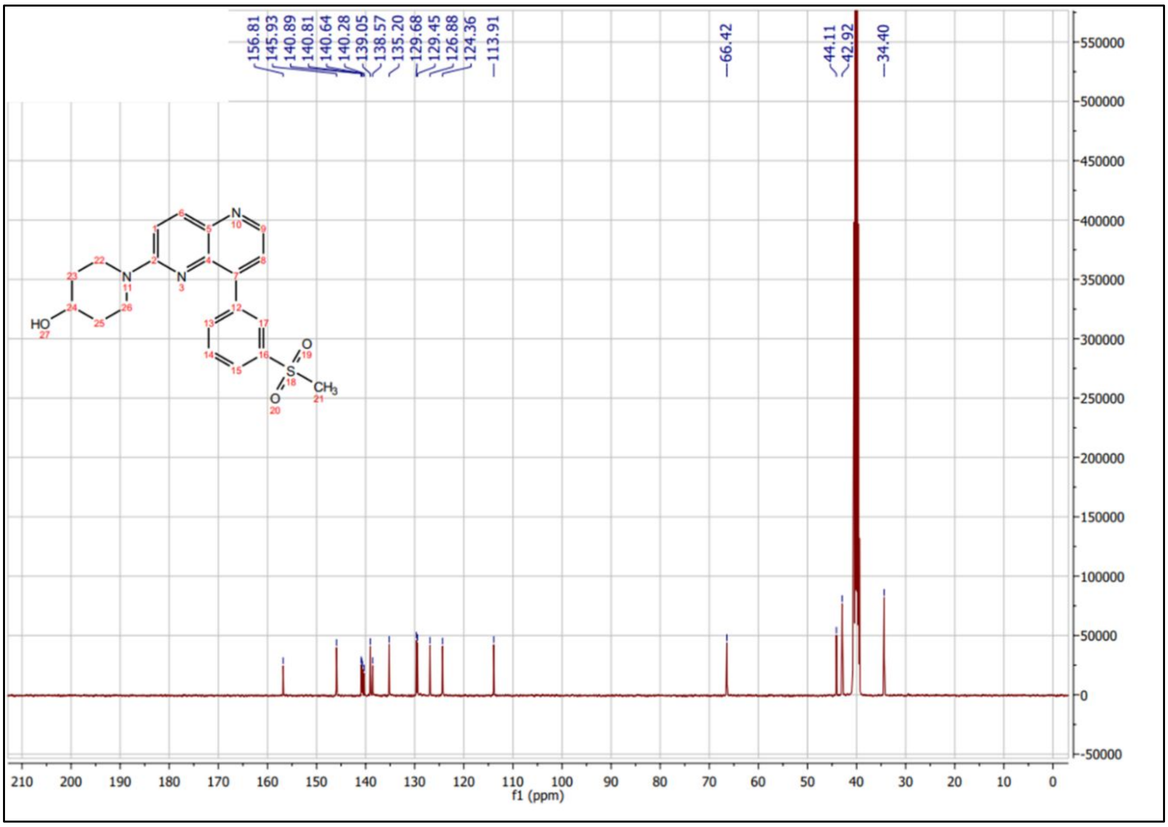

2.2 Analytical spectra for compound 22.

HPLC_UV and MS spectra of **22**

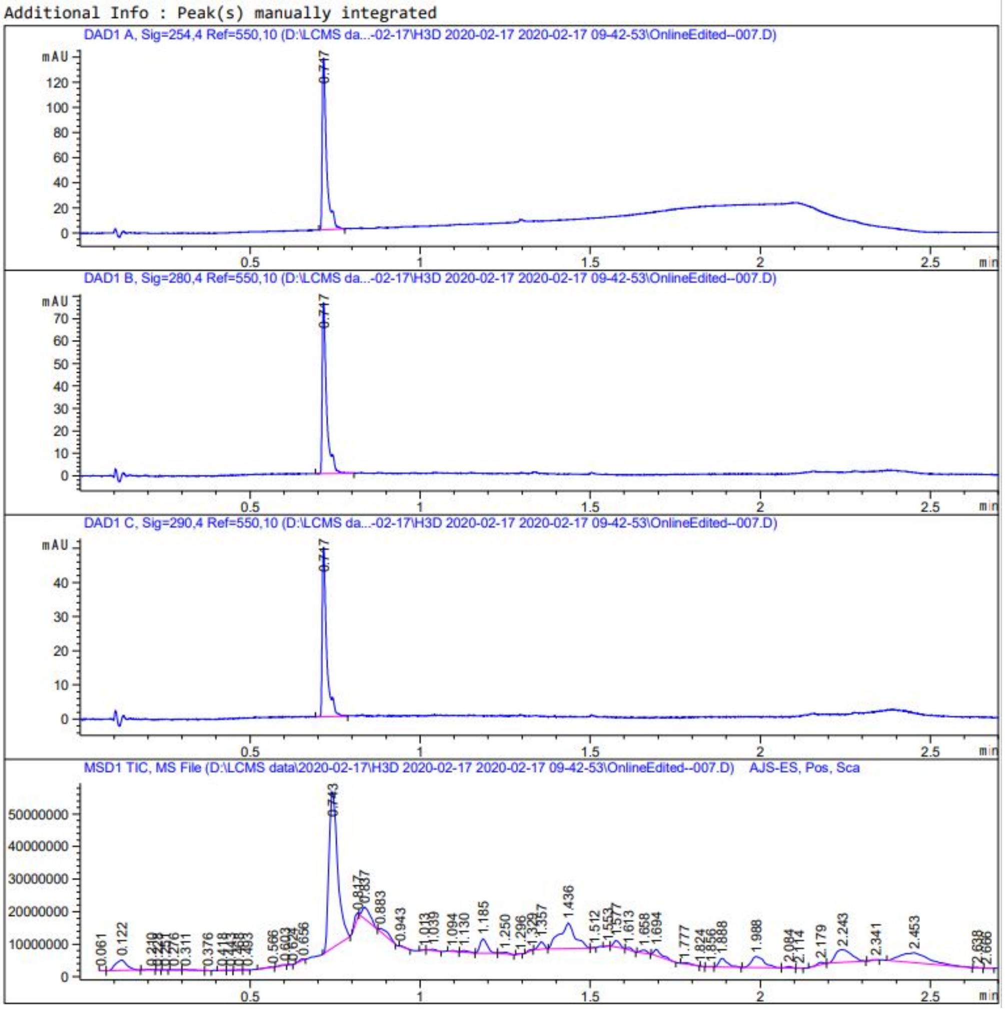

Signal 1: DAD1 A, Sig=254,4 Ref=550,10

Peak #	RetTime [min]	Type	Width [min]	Area [mAU*s]	Height [mAU]	Area %
1	0.717	BB	0.0126	117.96201	136.43069	100.0000
Totals :				117.96201	136.43069	

Signal 2: DAD1 B, Sig=280,4 Ref=550,10

Peak #	RetTime [min]	Type	Width [min]	Area [mAU*s]	Height [mAU]	Area %
1	0.717	BB	0.0127	66.77246	76.32366	100.0000
Totals :				66.77246	76.32366	

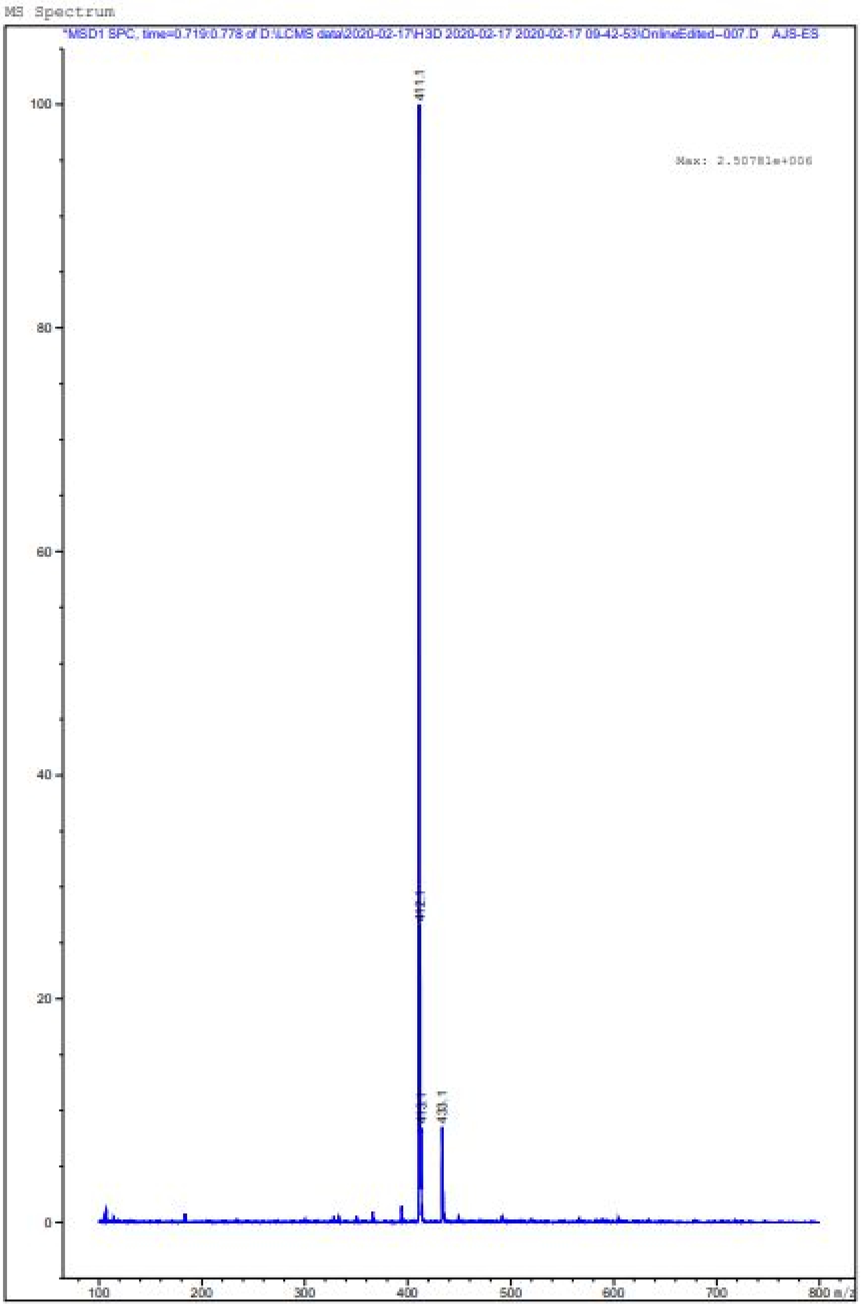
MS Spectrum
*MSD1 SPC, time=0.719:0.778 of D:\LCMS data\2020-02-17\H3D 2020-02-17 2020-02-17 09-42-53\OnlineEdited--007.D AJS-ES
Max: 2.50781e+006
411.1
433.1
100
80
60
40
20
0
100
200
300
400
500
600
700
800 m/z

^{1}H NMR spectrum of **22**

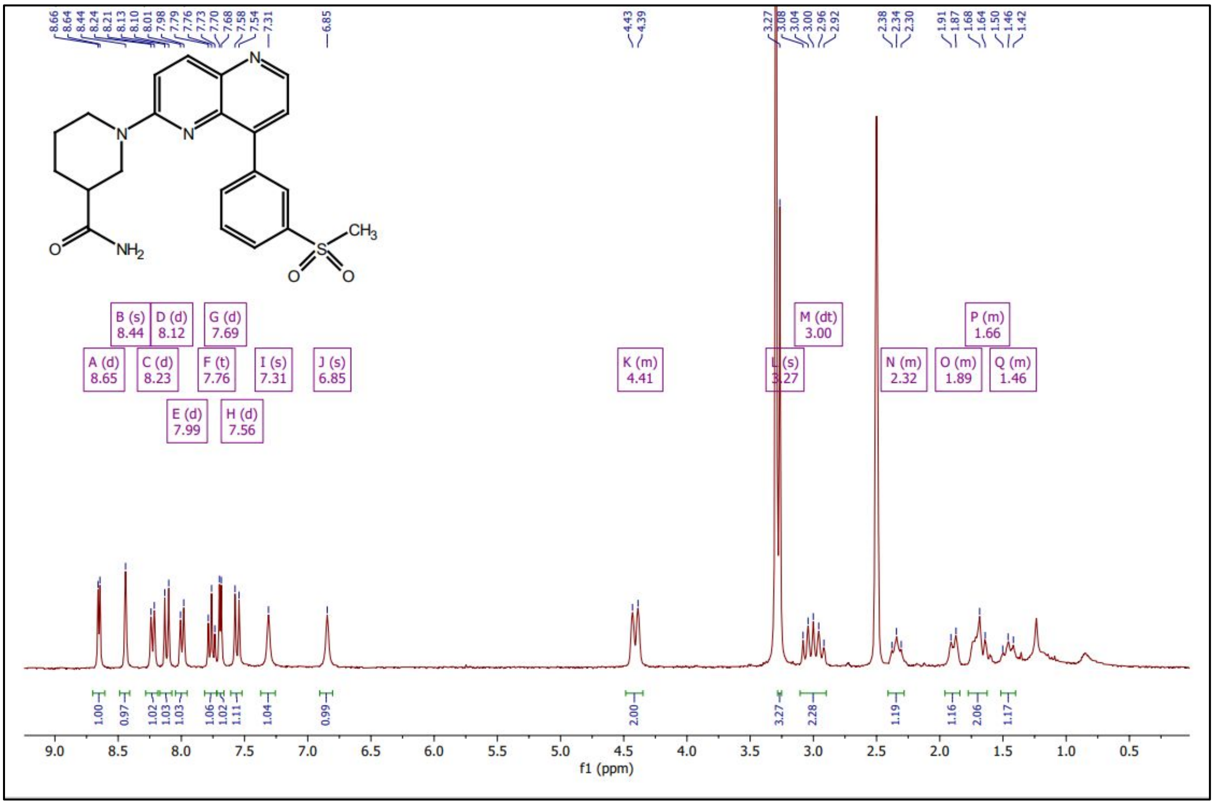

^{13}C NMR spectrum of **22**

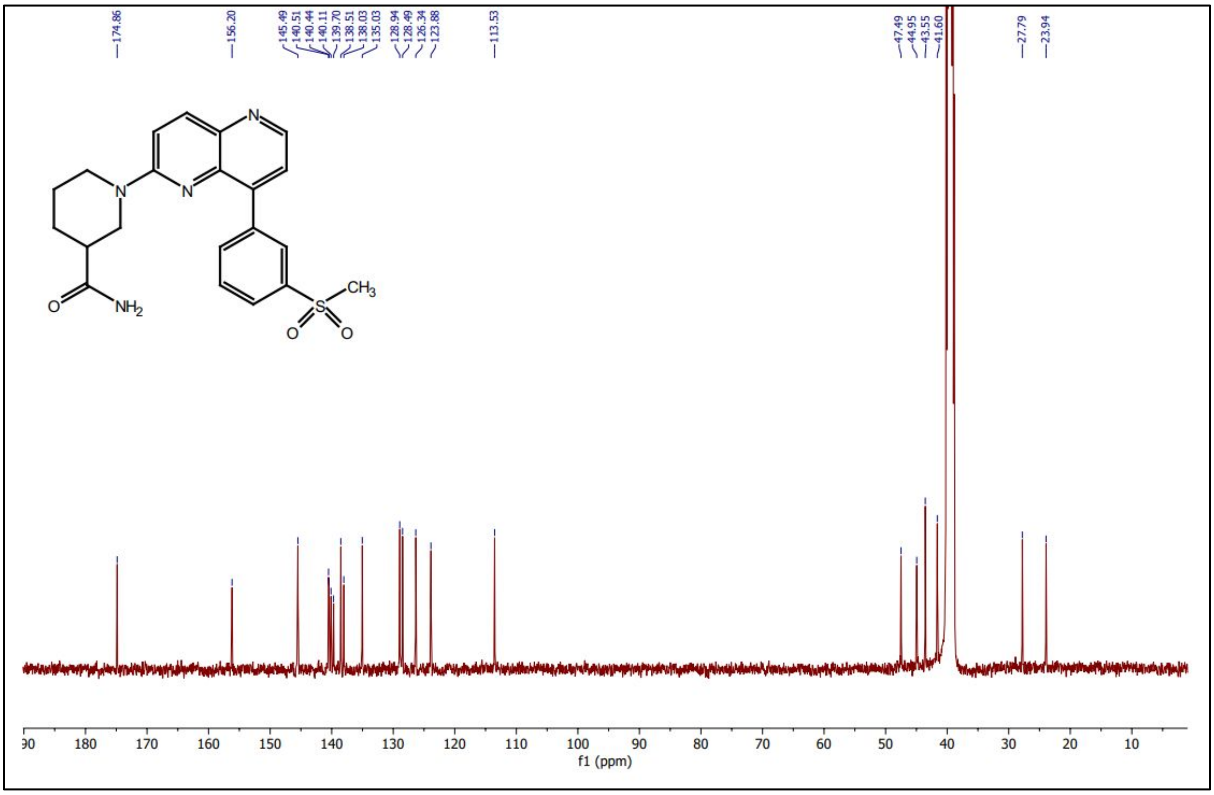

*2.3 Analytical spectra for compound **24**.*

HPLC_UV and MS spectra of **24**

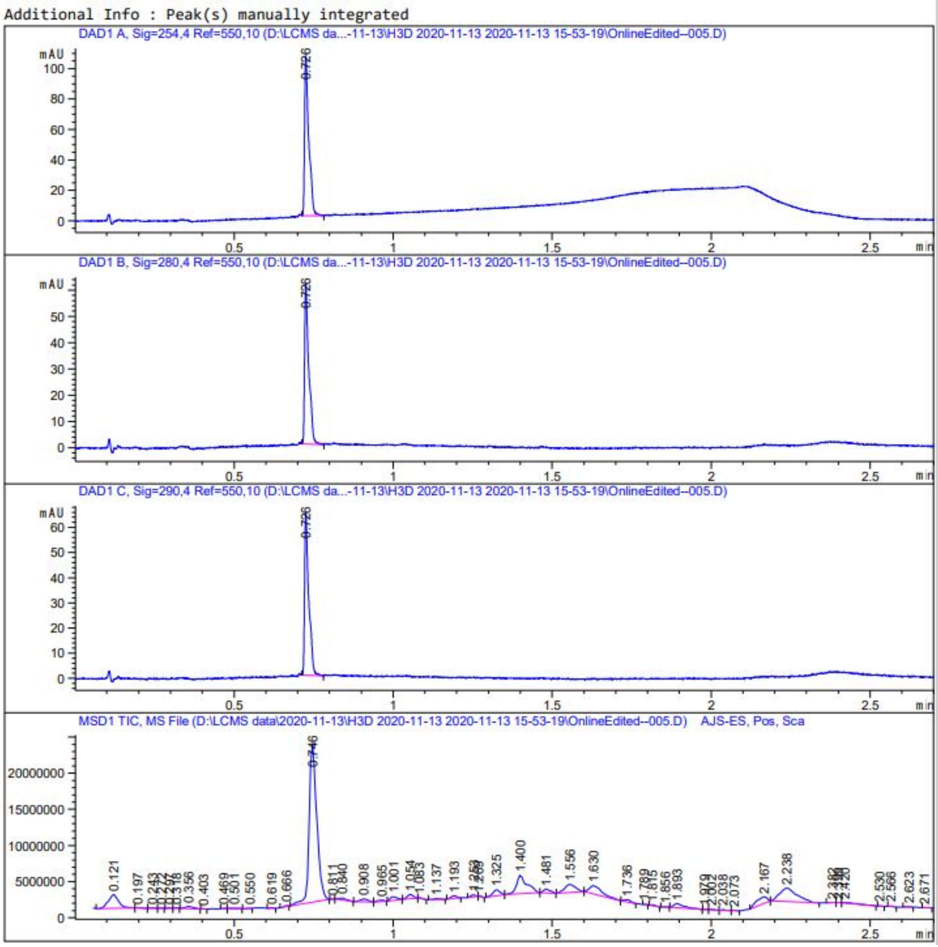

Signal 1: DAD1 A, Sig=254,4 Ref=550,10

Peak #	RetTime [min]	Type	Width [min]	Area [mAU*s]	Height [mAU]	Area %
1	0.726	BB	0.0133	96.52243	103.84609	100.0000
Totals :				96.52243	103.84609	

Signal 2: DAD1 B, Sig=280,4 Ref=550,10

Peak #	RetTime [min]	Type	Width [min]	Area [mAU*s]	Height [mAU]	Area %
1	0.726	BB	0.0133	55.68244	59.96647	100.0000
Totals :				55.68244	59.96647	

MS Spectrum

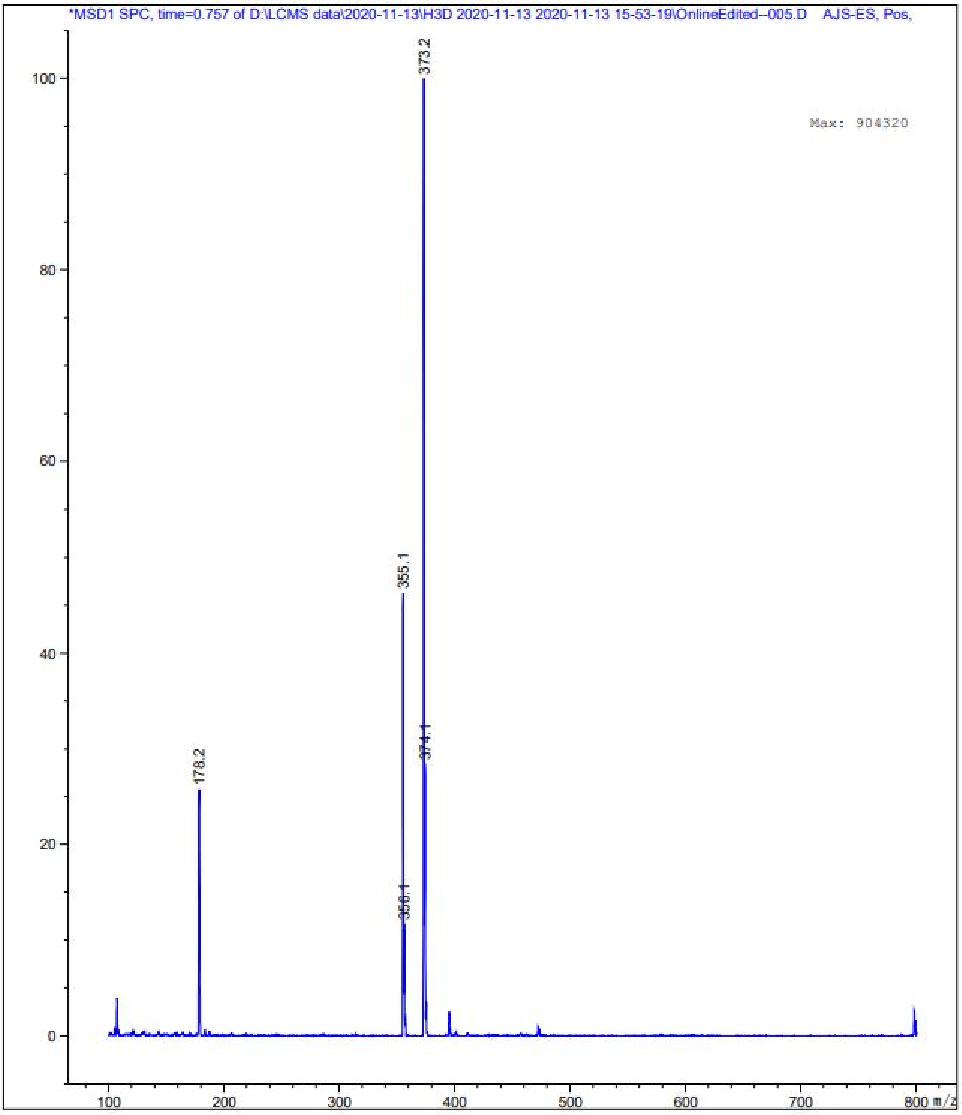

*MSD1 SPC, time=0.757 of D:\LCMS data\2020-11-13\H3D 2020-11-13 2020-11-13 15-53-19\OnlineEdited--005.D AJS-ES, Pos,
Max: 904320
373.2
355.1
374.1
178.2
356.1
100
80
60
40
20
0
100
200
300
400
500
600
700
800 m/z

^{1}H NMR spectrum of **24**

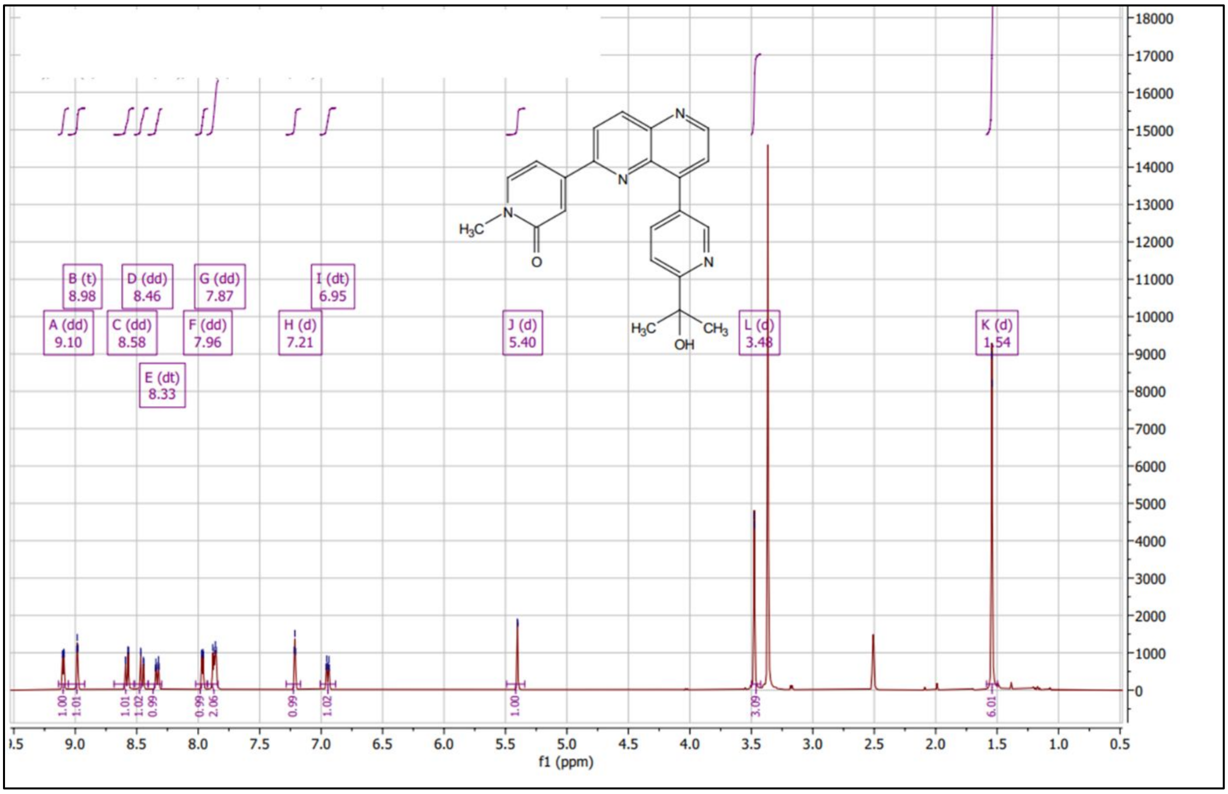

^{13}C NMR spectrum of **24**

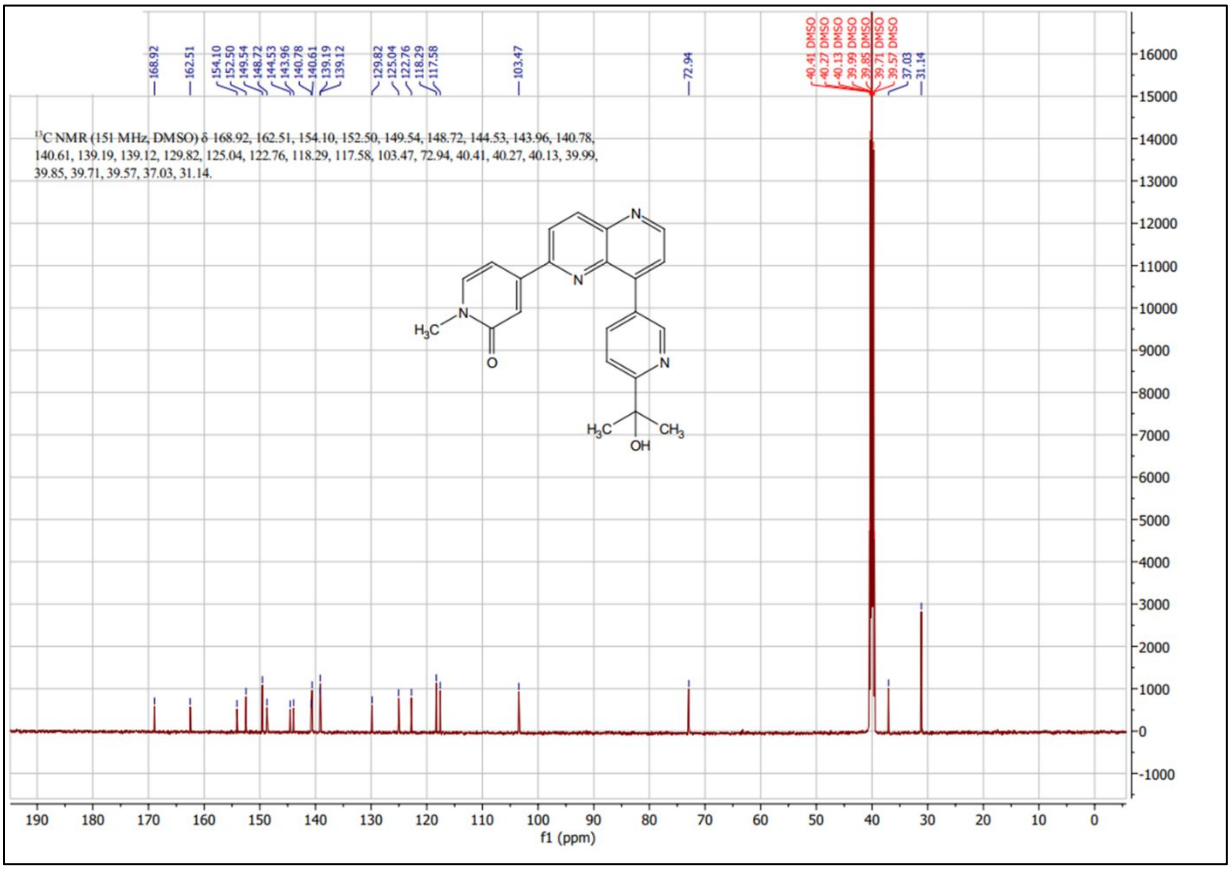

*2.4 Analytical spectra for compound **27**.*

HPLC_UV and MS spectra of **27**

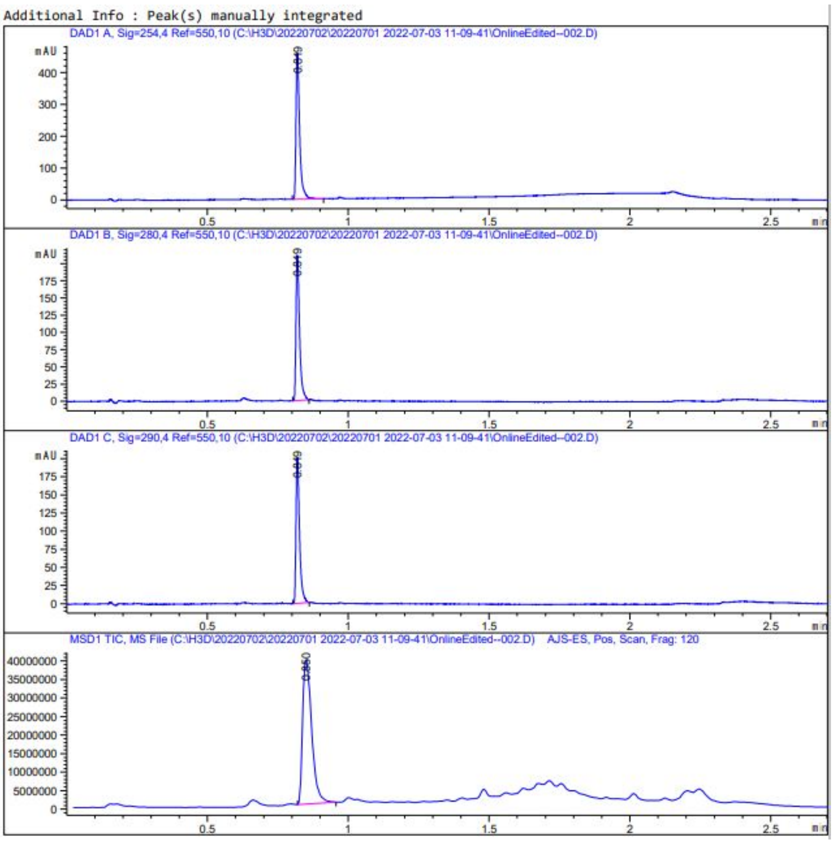

Signal 1: DAD1 A, Sig=254,4 Ref=550,10

Peak #	RetTime [min]	Type	Width [min]	Area [mAU*s]	Height [mAU]	Area %
1	0.819	BB	0.0142	416.86292	456.53391	100.0000
Totals :				416.86292	456.53391	

Signal 2: DAD1 B, Sig=280,4 Ref=550,10

Peak #	RetTime [min]	Type	Width [min]	Area [mAU*s]	Height [mAU]	Area %
1	0.819	BB	0.0139	186.67259	210.22226	100.0000
Totals :				186.67259	210.22226	

MS Spectrum

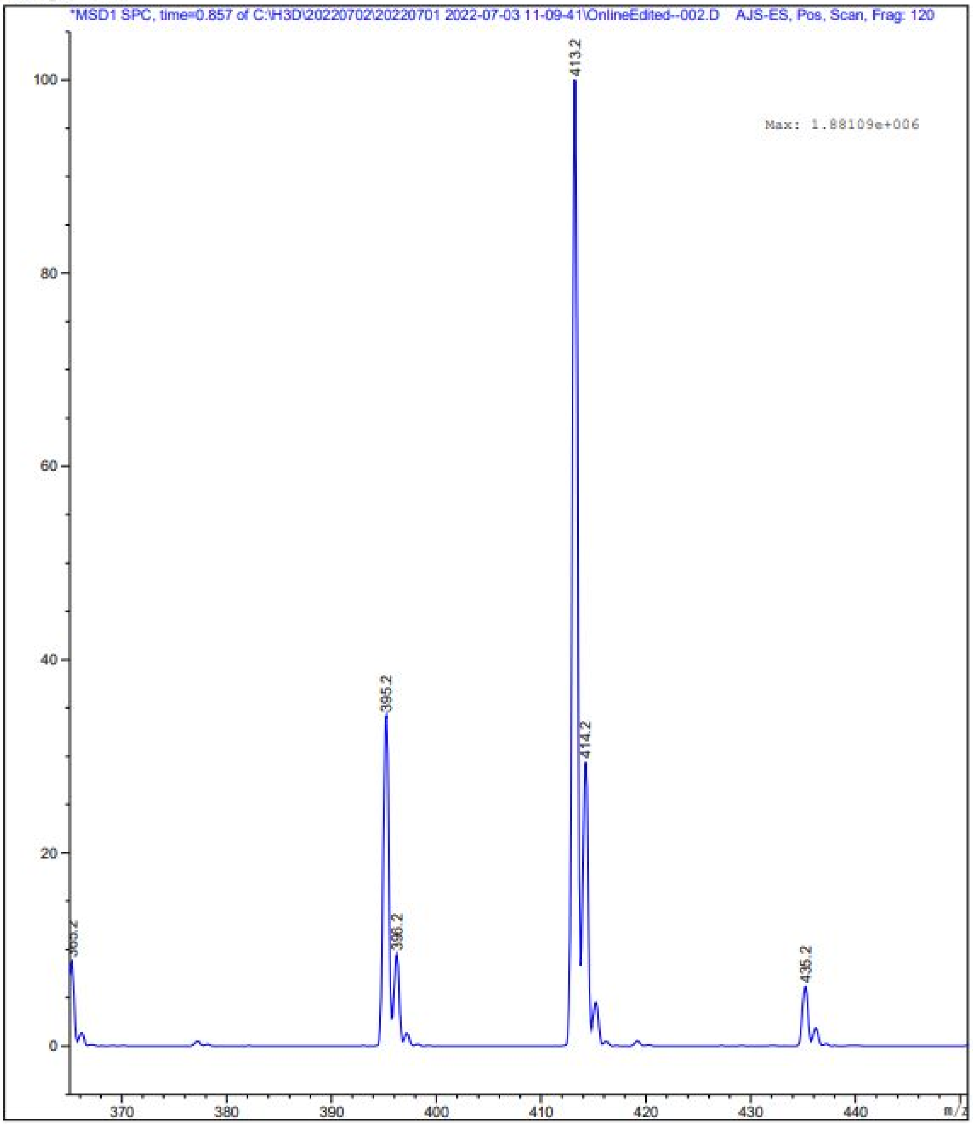

*MSD1 SPC, time=0.857 of C:\H3D\20220702\20220701 2022-07-03 11-09-41\OnlineEdited--002.D AJS-ES, Pos, Scan, Frag: 120
Max: 1.88109e+006
413.2
395.2
414.2
396.2
435.2
100
80
60
40
20
0
370
380
390
400
410
420
430
440
m/z

^{1}H NMR spectrum of **27**

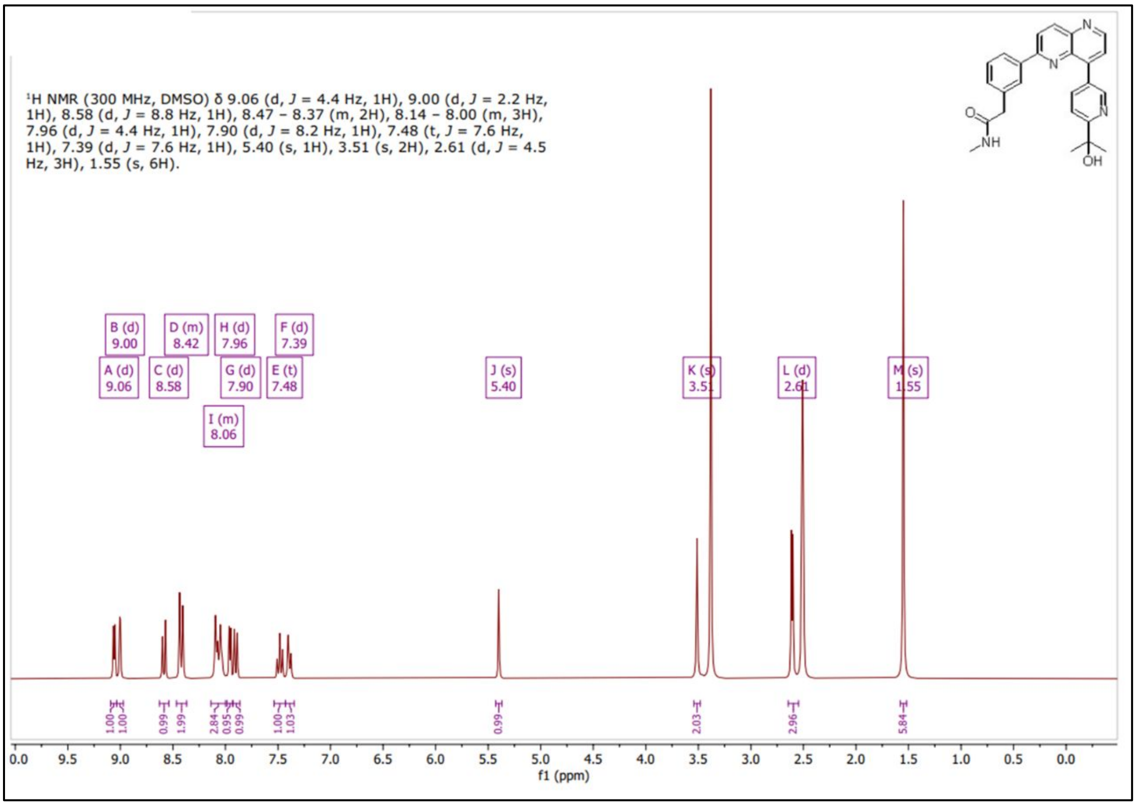

^{13}C NMR spectrum of **27**

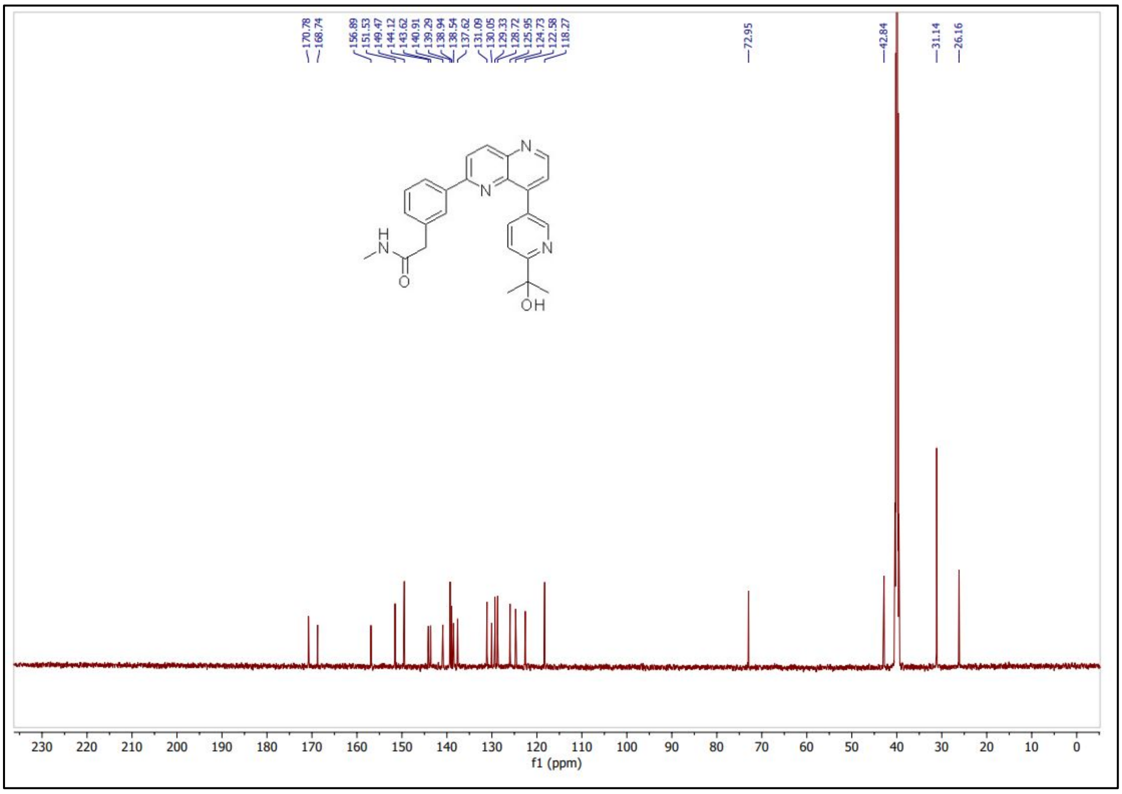

*2.5 Analytical spectra for compound **29**.*

HPLC_UV and MS spectra of **29**

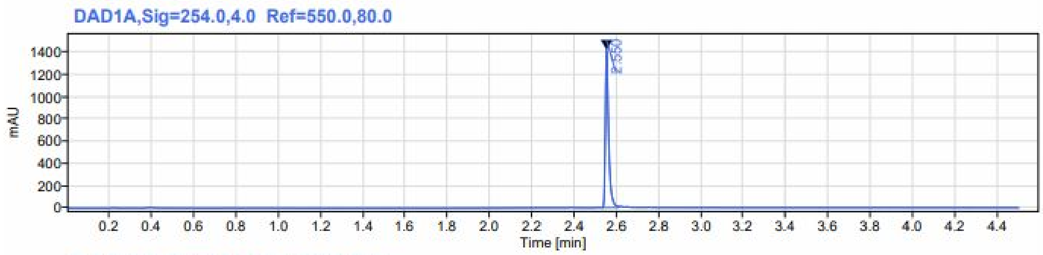

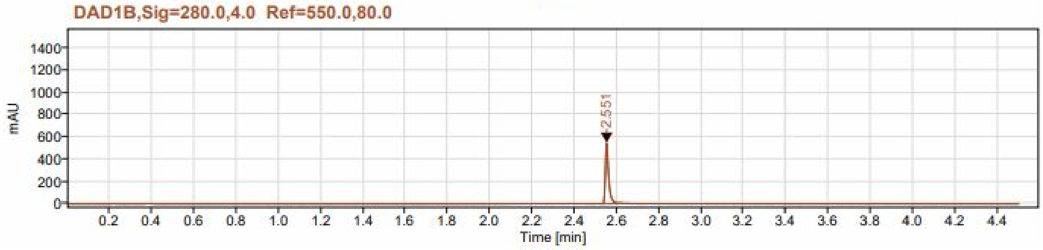

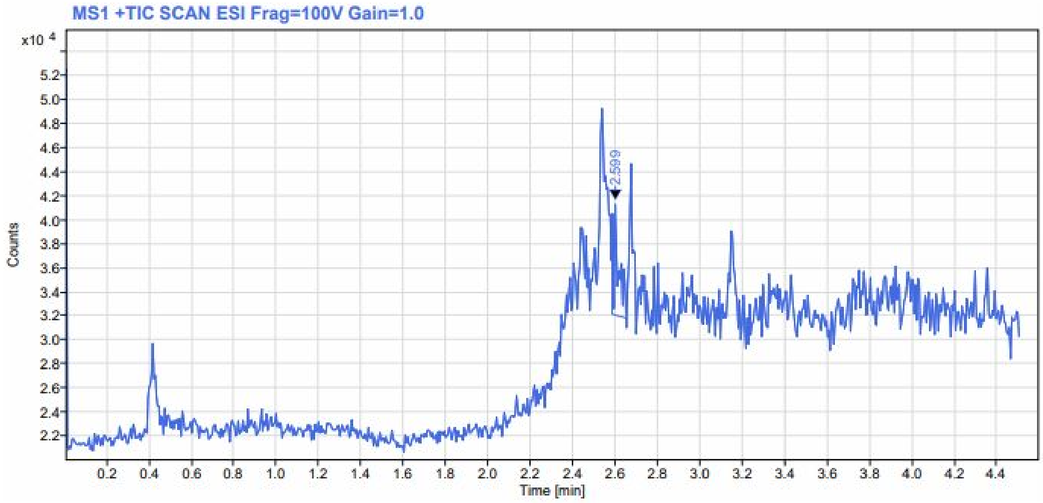

Signal: DAD1A,Sig=254.0,4.0 Ref=550.0,80.0

RT [min]	Type	Width [min]	Area	Height	Area%
2.550	BV	0.08	1559.35	1416.24	100.00
		**Sum**	**1559.35**		

Signal: DAD1B,Sig=280.0,4.0 Ref=550.0,80.0

RT [min]	Type	Width [min]	Area	Height	Area%
2.551	BV	0.08	595.88	544.38	100.00
		**Sum**	**595.88**		

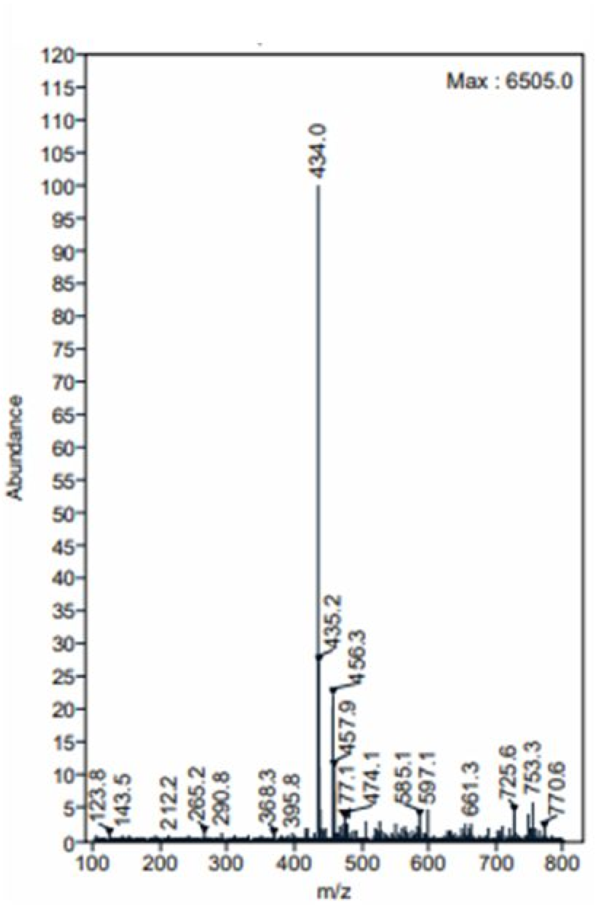
Max : 6505.0
Abundance
m/z
434.0
435.2
456.3
457.9
477.1
474.1
585.1
597.1
661.3
725.6
753.3
770.6
123.8
143.5
212.2
265.2
290.8
368.3
395.8

^{1}H NMR spectrum of **29**

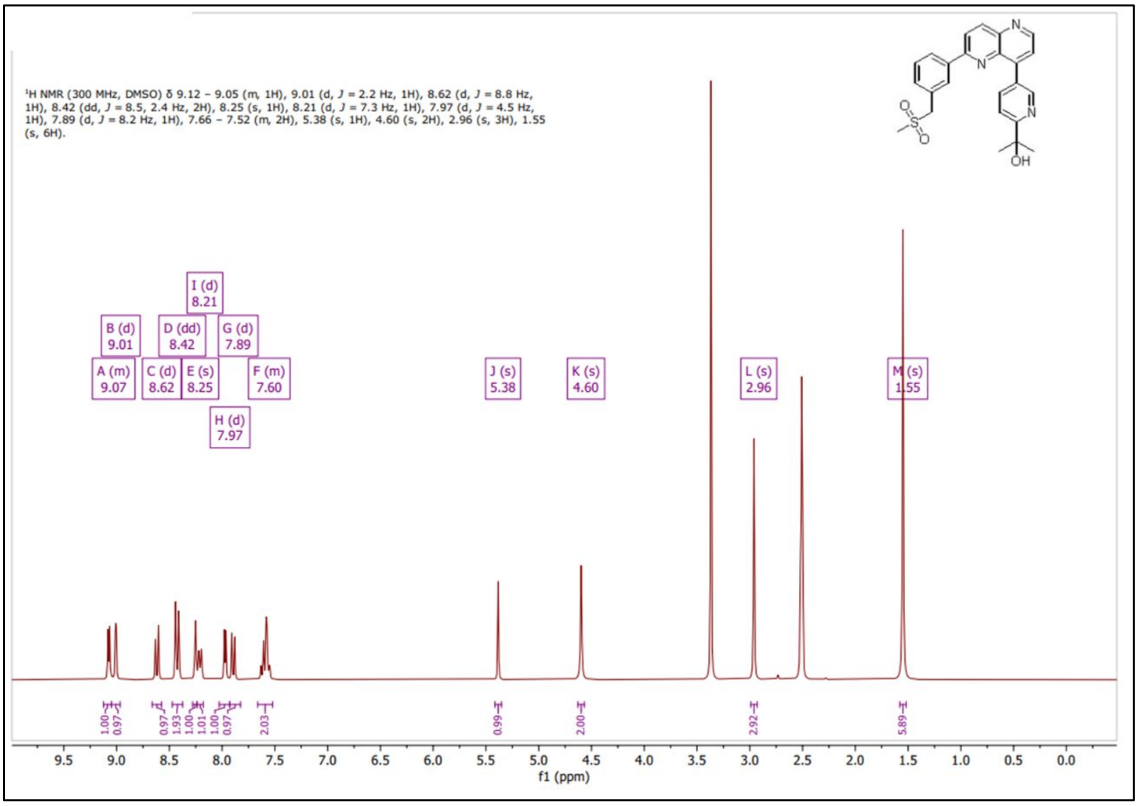

^{13}C NMR spectrum of **29**

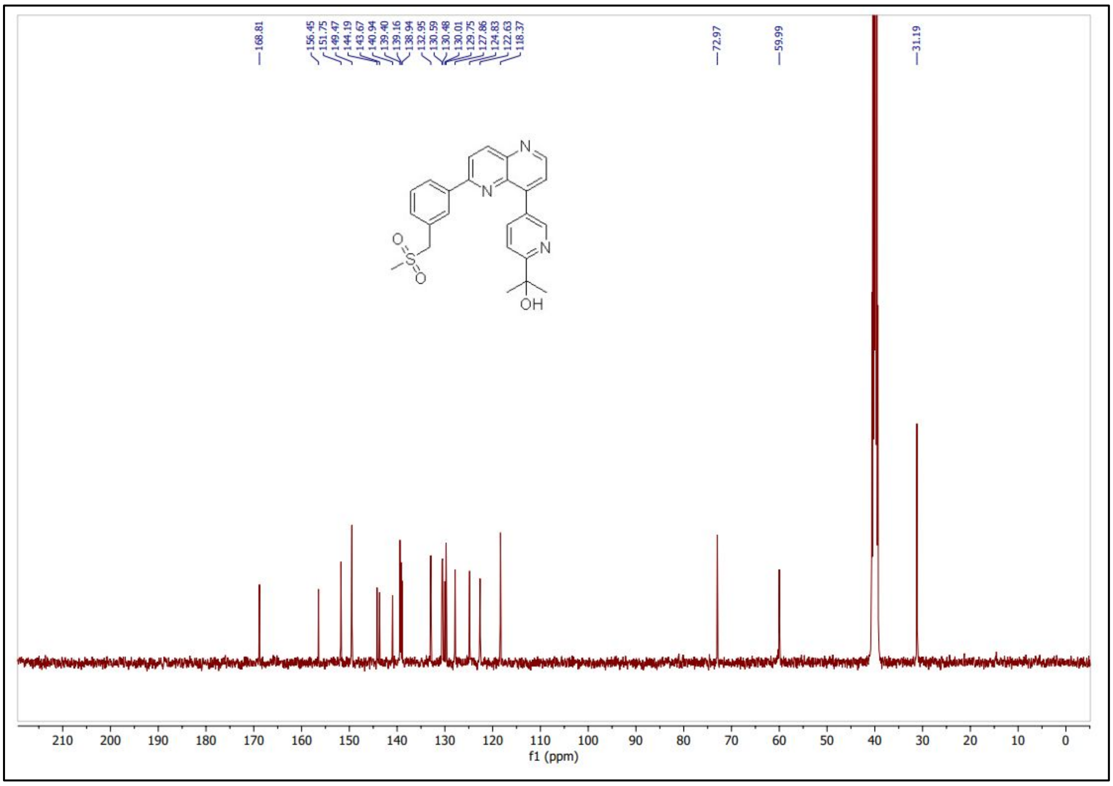

*2.6 Analytical spectra for compound **37**.*

HPLC_UV and MS spectra of **37**

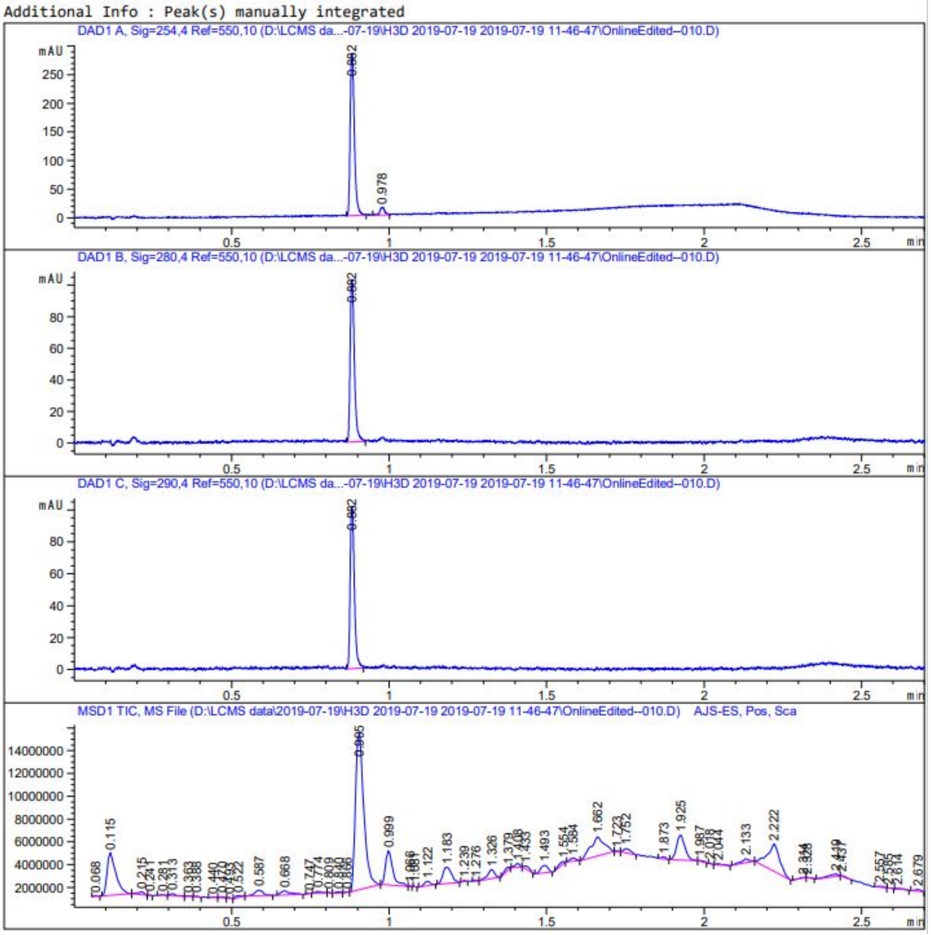

Signal 1: DAD1 A, Sig=254,4 Ref=550,10

Peak #	RetTime [min]	Type	Width [min]	Area [mAU*s]	Height [mAU]	Area %
1	0.882	BB	0.0143	262.46216	282.88861	95.0328
2	0.978	BB	0.0162	13.71848	13.13281	4.9672
Totals :				276.18064	296.02142	

Signal 2: DAD1 B, Sig=280,4 Ref=550,10

Peak #	RetTime [min]	Type	Width [min]	Area [mAU*s]	Height [mAU]	Area %
1	0.882	BB	0.0145	96.04802	102.13979	100.0000
Totals :				96.04802	102.13979	

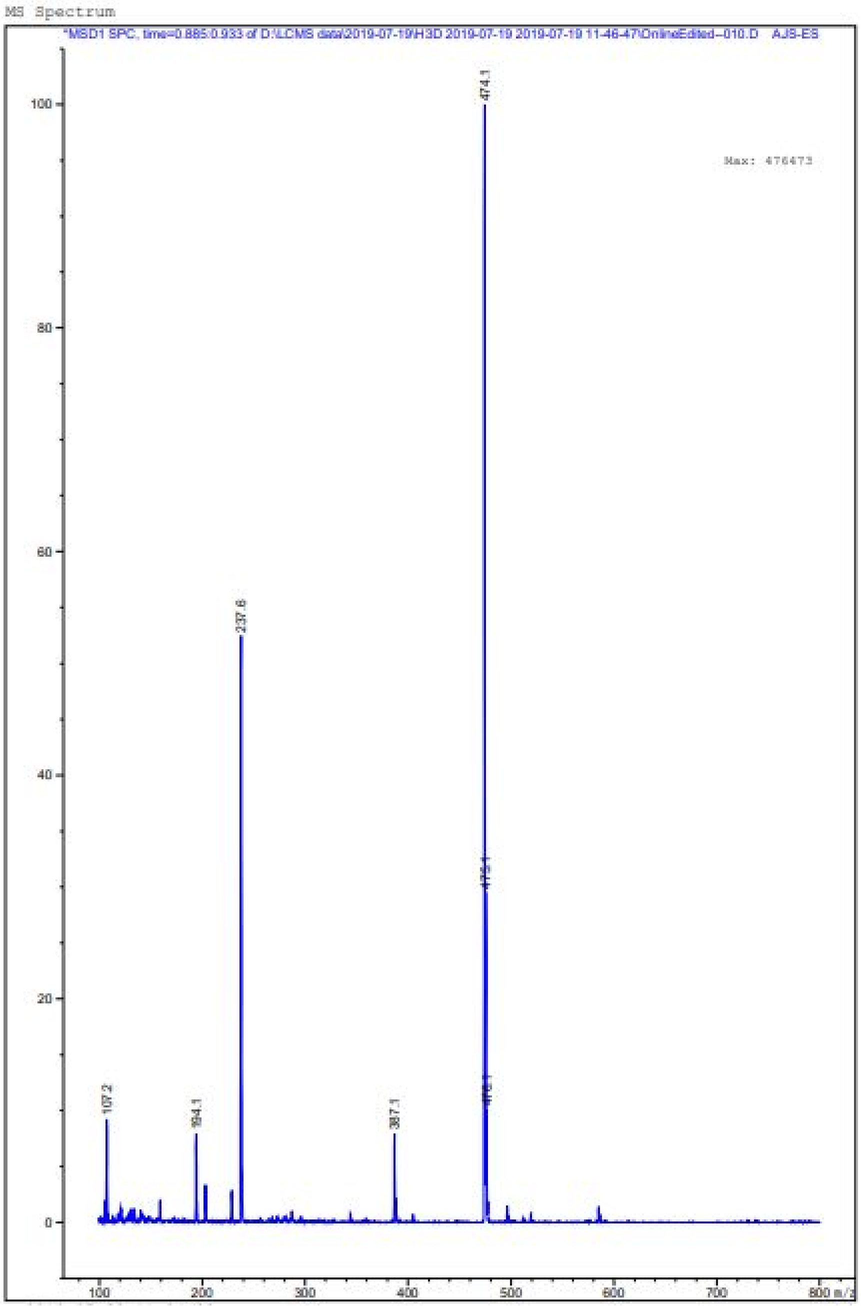
MS Spectrum
*MSD1 SPC, time=0.885:0.933 of D:\LCMS data\2019-07-19\H3D 2019-07-19 2019-07-19 11-46-47\OnlineEdited--010.D AJS-ES
Max: 476473
474.1
237.6
107.2
194.1
387.1
100
80
60
40
20
0
100
200
300
400
500
600
700
800 m/z

^{1}H NMR spectrum of **37**

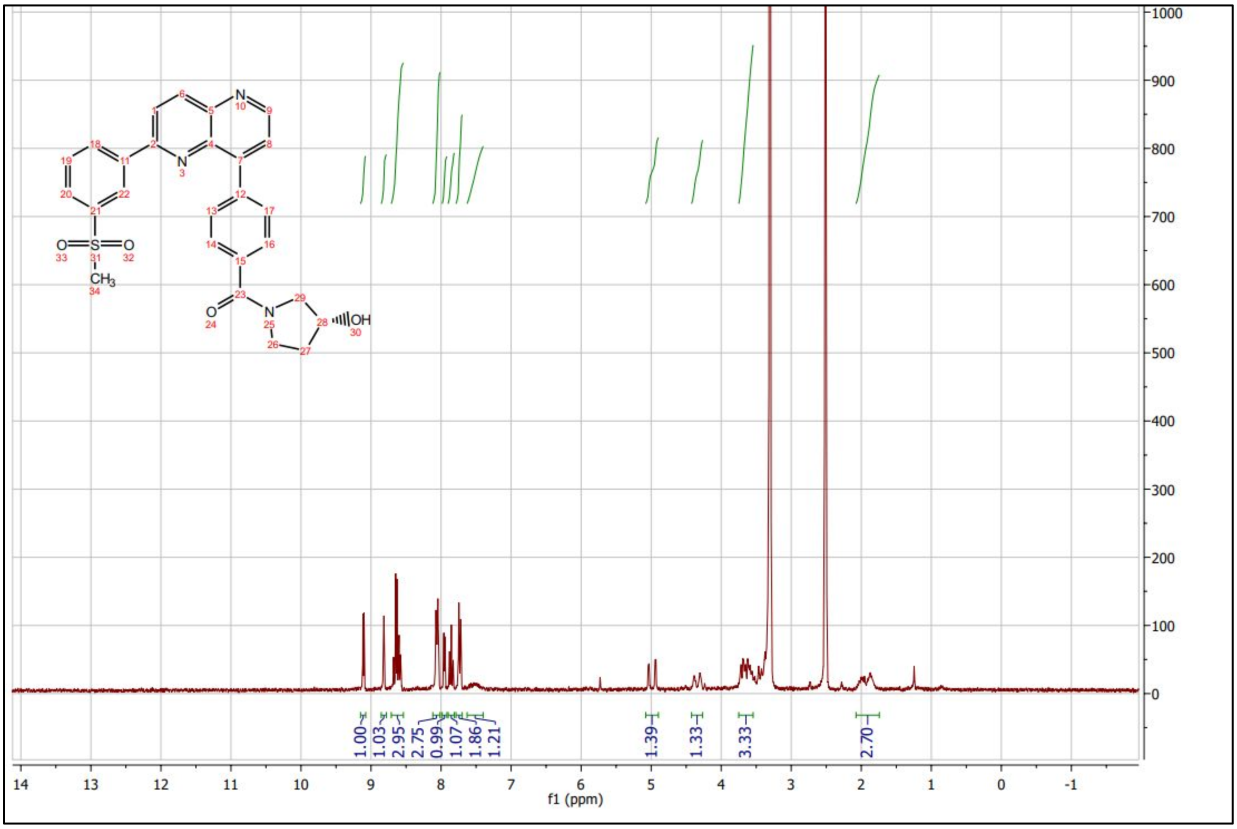

^{13}C NMR spectrum of **37**

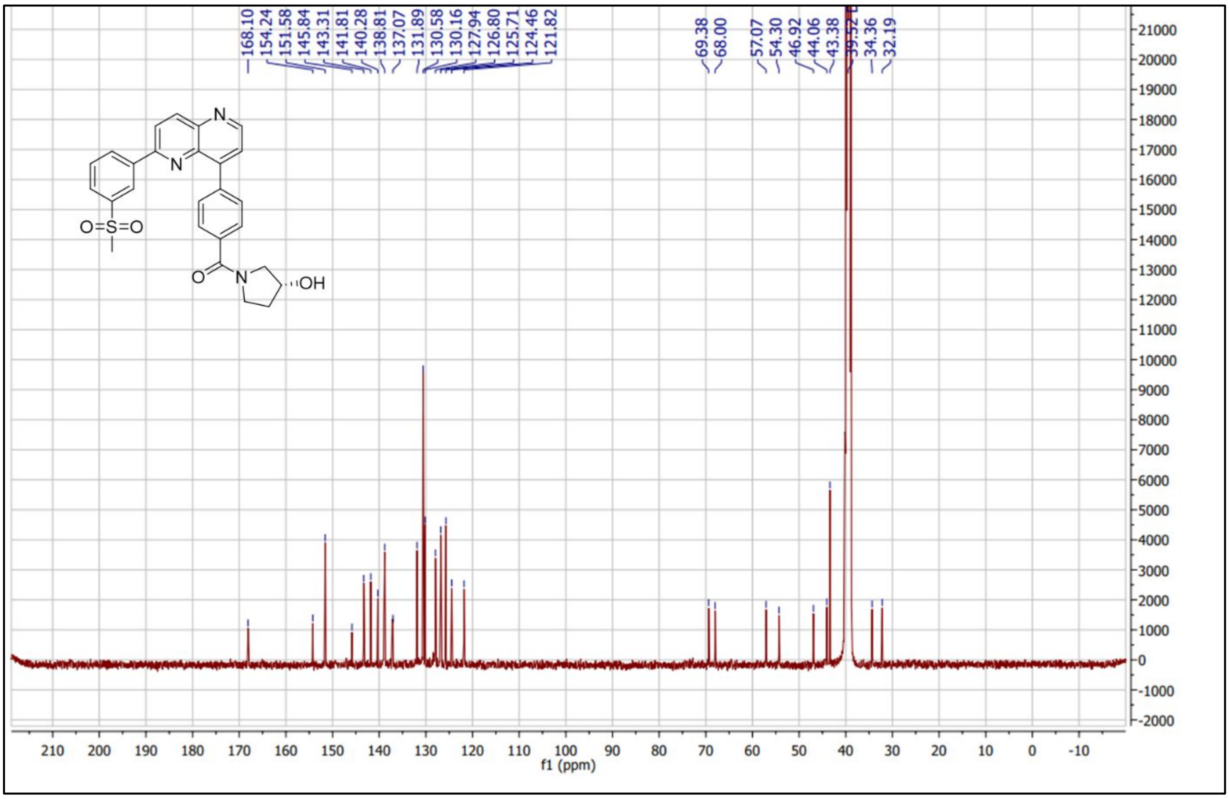

3. *In vitro* biological assays

3.1 Parasite culture

Human malaria parasites were cultured as described previously with minor modifications.[2] Several culture-adapted strains of *Plasmodium falciparum* were used for the different assessments. Parasites were maintained at 5-10% parasitemia in human erythrocytes (type O+) suspended in RPMI-1640 growth media supplemented with HEPES, glucose, bicarbonate and Albumax-II. Cultures were incubated at 37˚C in a mixture of 3% O_2 and 4% CO_2 in nitrogen and had growth media replenished daily to ensure viability.

3.2 Antiplasmodial activity assessments

A full dose-response assessment was performed for all compounds in a 96-well plate to determine the concentration inhibiting 50% of parasite growth (IC_{50}–value), with parasite survival measured using lactate dehydrogenase (pLDH) activity as a proxy.[3] Samples were prepared to a 10 mmol/L stock solution in 100% DMSO and stored at room temperature until testing. Dilutions to the desired starting concentration were freshly prepared in growth media on each occasion of the experiment. The standard antimalarial drugs chloroquine and artesunate were used as the reference drug in all experiments. The highest concentration of solvent to which the parasites were exposed was <0.5% and has no measurable effect on the parasite viability. The assay plate was incubated at 37˚C for 72h in a sealed gas chamber under 3% O_2 and 4% CO_2 in nitrogen.

For activity assessed using the pLDH method, the wells in the assay plate were gently resuspended after 72h, and 15μL from each well was transferred to a corresponding well in a duplicate plate containing 100 μL of Malstat reagent and 25 μL of nitroblue tetrazolium solution. Plates were left to develop for 20 minutes in the dark and then absorbance of each well was quantified using a spectrophotometer at 620 nM wavelength. Regression analysis was performed using the Dotmatics software platform to quantify the IC_{50}.

For activity measured using DNA incorporation, tritiated hypoxanthine was added to each well after 54h and cultures returned to the incubator for the remainder of the 72h period. After that, cells were harvested, washed and transferred to a scintillation counter to determine uptake. Relative amounts were plotted and regression analysis carried out using Graphpad Prism software to determine the IC_{50} value.

3.3 Cytotoxicity assessment

The IC_{50} against mammalian cells (human HepG2 or Chinese Hamster Ovary (CHO)) was determined using the MTT assay over 48h as described.[4] Samples were prepared to a 10 mmol/L stock solution in 100% DMSO and stored at room temperature until testing. Dilutions to the desired starting concentration were freshly prepared in growth media on each occasion of the experiment. Cells were plated 24h prior to exposure and allowed to adhere to the well surfaces.

After 24h, media was aspirated, compounds and fresh media were introduced and plates were returned to the incubator for a further 44h of growth; thereafter 25mL of sterile MTT dye was added to each well and plates were incubated for the final 4h of the experiment. Plates were centrifuged for 10 minutes to concentrate the reduced dye crystals; the supernatant was aspirated and 50mL DMSO added to dissolve the dye. Absorbance was measured at 540nM, and the IC_{50} determined by regression analysis of these values using the Dotmatics software platform.

*3.4 Pv*PI4K enzyme assay

Full-length *Pv*PI4K (PVX_098050) recombinant protein was expressed in a baculovirus-insect cell expression system and purified as previously described.[5,6] Briefly, N-terminal His-tagged recombinant *Pv*PI4K protein was purified using a HisTrap HP column (GE Healthcare), followed by size exclusion chromatography (HiLoad 16/600 Superdex 200 pg column, GE Healthcare). Final buffer composition of purified protein was 20 mM HEPES pH 7.5, 500 mM NaCl, 5% (v/v) glycerol, 10 mM β-mercaptoethanol.

*Pv*PI4K kinase inhibition assays were performed using the ADP-Glo kinase assay kit (Promega) to measure ADP formation as described previously.[7] L-alpha-phosphatidylinositol (PI; Avanti Polar Lipid, cat. 840042P) dissolved in 3% n-Octylglucoside to a stock concentration of 20 mg/mL was used as the lipid substrate. Briefly, a 3-fold serial dilution of each inhibitor was carried out in DMSO and inhibitors were subsequently diluted into assay buffer (25 mM HEPES pH 7.4, 100 mM NaCl, 3 mM MgCl2, 1 mM DTT, 0.025 mg/ml BSA, 0.2% (v/v) Triton-X-100) to 1.5 × the final required concentration. 2 μL of each inhibitor dilution was transferred into a white 384-shallow well plate (Nunc #264706). A MANTIS® Liquid Handler (Formulatrix) was used to dispense the remaining assay components. 0.5 μL *Pv*PI4K protein was added and following a 5-minute pre-incubation with inhibitor, 0.5 μL substrate buffer (ATP and PI) was added to each well. The final 3 μL kinase reaction contains ~6 nM *Pv*PI4K protein, 10 μM ATP, 0.1 mg/ml PI, 1% (v/v) DMSO and inhibitor in assay buffer. Reactions were incubated for 40 minutes at 22˚C (resulting in < 10% ATP conversion). ADP formation was measured using the ADP-Glo Kinase Kit (Promega). Briefly, 2 μL ADP-Glo reagent containing 10 mM MgCl2 was added to each well and incubated for 40 minutes at 22˚C to deplete the remaining ATP. 2 μL of Kinase Detection Reagent was then added and the reaction was incubated for a further 30 minutes at 22˚C. The plate was sealed with an adhesive foil seal for all incubation steps. Luminescent signal was measured using the EnSpire Multimode Plate Reader (PerkinElmer). The data was normalised based on the 100% activity controls (1% DMSO only) and the 100% inhibition controls (10 μM MLN0128 (sapanisertib).[7] Mean IC_{50} values were calculated from $N \geq 2$ independent experiments, each with technical duplicates (log(inhibitor) vs. normalized response - Variable slope). IC_{50} values within 3-fold from independent experiments are considered reproducible.

3.5 Human lipid kinase screening

The biochemical inhibitory activities (IC_{50} values) of the compounds against the human kinases *Hs*PI3Kα, *Hs*PI4Kβ, *Hs*MAP4K4 and *Hs*MINK1 were measured at Reaction Biology Corporation (Devault, Pennsylvania, USA) with an ADP-Glo Kinase Assay platform (Promega). The compounds were tested in 10-dose IC_{50} mode with a three-fold serial dilution starting at 10 μM. The concentration of ATP was 10 μM. A detailed description of the ADP-Glo assay is available online (https://www.promega.co.uk/products/cell-signaling/kinaseassays-and-kinase-biology/adp-glo-kinase-assay/?catNum=V6930).

3.6 PfPI4K-mutant cross-resistance assays

In vitro susceptibility of *Pf*PI4K mutant parasites to compounds **1**, **27**, **37**, and KDU691 was determined using the SYBR green I-based fluorescence assay[8] against 3 *P. falciparum* lines including wildtype Dd2, PI4K-S743F+H1484Y, and PI4K-S1320L+L1418F. Asynchronous *P. falciparum* parasites were diluted to 1% parasitaemia at 1% haematocrit (final concentration in the assay plate), and incubated with a two-fold serial dilution of compounds in complete medium. Untreated parasites or parasites treated with 1 μM dihydroartemisinin (DHA) were included in the assay plate as controls. Parasite growth was evaluated after 72h by lysing parasites using 2× lysis buffer (10 mM Tris-HCl, 5 mM EDTA, 0.1% w/v saponin, and 1% v/v Triton X-100, supplemented with 2× SYBR Green I (Molecular Probes)). The fluorescence was measured using a FluorStar Omega v5.11 plate reader. The half-maximal inhibitory concentration (IC_{50}) analysis was performed using GraphPad Prism v10 and statistical significance was determined by two-sided Mann-Whitney *U* test. All assay were performed in technical triplicates with at least four biological replicates, as noted in the figure legends.

3.7 P. falciparum PI4K conditional knockdown assays

Compound susceptibility assays using *P. falciparum* PI4K cKD lines were carried out as previously described.[7] Briefly, synchronous ring-stage PI4K (PF3D7_0509800) cKD parasites, as well as a control parasite line expressing an aptamer-regulatable fluorescent protein were maintained in the presence of high aTc (500 nM) or no aTc and distributed into 384-well polystyrene microplates (Corning). Stock solutions of compounds were serially diluted and transferred to the parasite-containing plates using the Janus platform (PerkinElmer). DMSO and dihydroartemisinin treatment (500 nM) served as reference controls. Luminescence was measured after 72 hours using the *Renilla*-Glo Luciferase Assay System (Promega E2750) and the GloMax Discover Multimode Microplate Reader (Promega), and IC_{50} values were obtained from corrected dose-response curves using Graph-Pad Prism (Table S1).

4. In vitro ADME assays.

4.1 Solubility

Solubility was performed using a miniaturized shake flask method. 10 mM stock solutions of each compound were used to prepare calibration standards (10-220 µM) in DMSO. The same 10mM stock solutions were accurately dispensed in duplicate into 96-well plates and the DMSO dried down (MiVac GeneVac, 90 min, 37 ˚C). Thereafter, the samples were reconstituted (200 µM) in aqueous solution and shaken (20 hours, 25 ˚C). The solutions were analysed by means of HPLC-DAD (Agilent 1200 Rapid Resolution HPLC with a diode array detector). Solubility was then determined using the peak areas of the aqueous samples and the best fit calibration curves constructed using the calibration standards.[9]

4.2 LogD

The LogD assay was performed in triplicate using a shake-flask procedure. 10 mM stock solutions of each test compound were used to spike (100 µM) a 1:1 mixture of phosphate buffer (pH 7.4) and *n*-octanol. The solutions were shaken vigorously (1500 rpm) on an orbital shaker for 3 hours at room temperature. Thereafter the samples were centrifuged in order to fully separate the two immiscible fluids. The samples were analyzed by HPLC-DAD (Agilent 1200 Rapid Resolution HPLC with a diode array detector) and the amount of compound in the buffer and *n*-octanol were used to determine the partition coefficient, $LogD_{7.4}$.[10]

4.3 In vitro Microsomal stability

The *in vitro* microsomal stability assay was performed in duplicate in a 96-well micro titre plate. The test compounds (1 µM) were incubated individually in mouse, rat and pooled human liver microsomes (final protein concentration of 0.4 mg/mL; XenoTech, Lenexa, KS), suspended in 0.1M phosphate buffer (pH 7.4) for predetermined time points, in the presence and absence of the cofactor NADPH (1 mM). Reactions were quenched by adding 300 µL of ice-cold acetonitrile containing internal standard (carbamazepine, 0.0236 µg/mL). The samples were centrifuged and test in the supernatant were analyzed by means of LC-MS/MS (Agilent Rapid Resolution HPLC, AB SCIEX 4500 MS). The relative loss of parent compound over time was monitored and plots (concentration vs. time) were prepared per compound to determine the first order rate constant for compound depletion. This was in turn used to calculate half-life, in vitro intrinsic clearance and in vivo hepatic extraction ratio.[11]

4.4 Plasma protein binding (PPB)

Plasma protein binding was determined by ultracentrifugation. In brief, pooled human plasma was spiked with test compound (5 μM) from a 10mM DMSO stock. An aliquot was immediately removed and quenched using ice cold acetonitrile containing internal standard (carbamazepine, 0.0236 μg/mL), and placed in the freezer. This served as the total concentration sample. After pre-incubation (37 ˚C for 1 hour) duplicate aliquots of the spiked plasma were transferred to ultra-centrifugation tubes, and ultracentrifuged for 4 hours (42000 rpm, 37 ˚C, Beckman Optima L-80XP). The samples were then analysed by LC-MS/MS (Agilent Rapid Resolution HPLC, AB SCIEX 4500 QTRAP MS). Protein binding was then calculated by comparing analyte:peak area ratios of the ultracentrifuged sample to those of the total concentration sample.

5. *In vivo* studies.

5.1 Ethics Statement

Animal studies were conducted at the Holistic Drug Discovery and Development (H3D) Centre Animal Research Facility, University of Cape Town (UCT). Ethical approval was granted by the UCT Animal Ethics Committee prior to study commencement (ethics approval reference number AEC 022_004 and AEC 021_015), and all procedures were performed in accordance with the South African National Standard (SANS 10386:008) for the Care and use of Animals for Scientific Purposes,[12] and guidelines from the Department of Health.[13]

5.2 Mice pharmacokinetics studies

Male BalbC mice were bred at the University of Cape Town Research Animal Facility, University of Cape Town, South Africa. Compound was administered intravenously (3 mg/kg) to male Balb/C mice (n=3) as a bolus of 10% (v/v) dimethylsulfoxide (DMSO), 60% (v/v) propylene glycol and 30% polyethyleneglycol (PEG) 400. The oral dose (10 mg/kg) was administered to mice (n=3) as an aqueous suspension containing 0.5% (w/v) hydroxypropylmethylcellulose (HPMC) and 0.2% (v/v) Tween 80. Mice were not fasted overnight and were allowed to eat ad libitum. Animals were permitted access ad libitum to water.

Sample analysis

Blood samples were collected from mice into heparinised microcentrifugation tubes at 0.17 (IV only), 0.5, 1, 3, 5, 7, 9, 24 hours after dosing and stored frozen (-80 ˚C) until analysis.

Bioanalytical method

The compound concentration was determined by LC-MS/MS. Samples were thawed and extracted by protein precipitation using acetonitrile containing an internal standard. The supernatant was then submitted for LC-MS/MS analysis. Calibration standards and quality controls prepared in drug-free whole mice blood were processed similarly. Elution of analytes was confirmed by multiple-reaction monitoring and compound concentrations were determined using the analyte response of the analytes relative to the calibration curve. The accuracy, precision, and recovery for each compound were within acceptable limits.

Calculation of pharmacokinetic parameters

Pharmacokinetic parameters were calculated by non-compartmental analysis using PK Solutions 2.0 (Summit Research Services, Montrose, CO, USA) using a method based on curve stripping.

5.3. In vivo efficacy and pharmacokinetics in malaria-infected humanized mice

P. falciparum infection of humanized mice

The antimalarial activity of **27** was determined in the *P. falciparum*-infected NSG mouse model, in 6- to 10-week-old, male NSG mice, weighing between 25 and 30 g, using methods previously described.[14] Briefly, NSG mice were intravenously engrafted daily with prepared human erythrocytes for 10 days, then the mice were intravenously injected in the tail vein with 2 x 10^7 asynchronous *Pf*3D7$^{0087/N9}$-infected human erythrocytes (day 0). *Pf*3D7$^{0087/N9}$ is a chloroquine-sensitive strain that was developed and selected for infection in NSG mice at GlaxoSmithKline, Tres Cantos, Spain. The infection was left to establish for 3 days before commencement of treatment on day 3. The percentage of human erythrocytes was maintained above 50% with daily engraftments until the experimental endpoint on day 7 after infection.

Administration and blood sampling

Compound **27** was administered as four consecutive 50 mg/kg doses, 24 hours apart, starting on the third day after infection with *P. falciparum*. Whole-blood PK and efficacy samples were collected via tail vein bleeding into lithium heparin-coated tubes. PK blood samples were collected at 0.5, 1, 3, 5, 7, 24.5, 48.5, 72.5 and 96 h after administration on day 3. PK samples were stored at -80˚C until bioanalysis. Efficacy blood samples were collected before treatment for all experimental groups on days 3, 4, 5, 6, and 7. These samples were processed immediately after collection, and the percentage of infected human erythrocytes, or the parasitemia, and the percentage engraftment measurements were determined by fluorescence using an Accuri C6 Plus flow cytometer and FlowJo 10.8 software (Becton, Dickinson and Company), as previously described.[14]

6. References

(1) Dziwornu, G. A.; Seanego, D.; Fienberg, S.; Clements, M.; Ferreira, J.; Sypu, V. S.; Samanta, S.; Bhana, A. D.; Korkor, C. M.; Garnie, L. F.; Teixeira, N.; Wicht, K. J.; Taylor, D.; Olckers, R.; Njoroge, M.; Gibhard, L.; Salomane, N.; Wittlin, S.; Mahato, R.; Chakraborty, A.; Sevilleno, N.; Coyle, R.; Lee, M. C. S.; Godoy, L. C.; Pasaje, C. F.; Niles, J. C.; Reader, J.; van der Watt, M.; Birkholtz, L.-M.; Bolscher, J. M.; de Bruijni, M. H. C.; Coulson, L. B.; Basarab, G. S.; Ghorpade, S. R.; Chibale, K. 2,8-Disubstituted-1,5-Naphthyridines as Dual Inhibitors of *Plasmodium Falciparum* Phosphatidylinositol-4-Kinase and Hemozoin Formation with *In Vivo* Efficacy. *J Med Chem* **2024**, *67* (13), 11401–11420. https://doi.org/10.1021/acs.jmedchem.4c01154.

(2) Trager, W.; Jensen, J. B. Human Malaria Parasites in Continuous Culture. *Science (1979)* **1976**, *193* (4254), 673–675. https://doi.org/10.1126/science.781840.

(3) Makler, M. T.; Ries, J. M.; Williams, J. A.; Bancroft, J. E.; Piper, R. C.; Gibbins, B. L.; Hinrichs, D. J. Parasite Lactate Dehydrogenase as an Assay for Plasmodium Falciparum Drug Sensitivity. *Am J Trop Med Hyg* **1993**, *48* (6), 739–741. https://doi.org/10.4269/ajtmh.1993.48.739.

(4) Mosmann, T. Rapid Colorimetric Assay for Cellular Growth and Survival: Application to Proliferation and Cytotoxicity Assays. *J Immunol Methods* **1983**, *65* (1–2), 55–63. https://doi.org/10.1016/0022-1759(83)90303-4.

(5) McNamara, C. W.; Lee, M. C. S.; Lim, C. S.; Lim, S. H.; Roland, J.; Nagle, A.; Simon, O.; Yeung, B. K. S.; Chatterjee, A. K.; McCormack, S. L.; Manary, M. J.; Zeeman, A.-M.; Dechering, K. J.; Kumar, T. R. S.; Henrich, P. P.; Gagaring, K.; Ibanez, M.; Kato, N.; Kuhen, K. L.; Fischli, C.; Rottmann, M.; Plouffe, D. M.; Bursulaya, B.; Meister, S.; Rameh, L.; Trappe, J.; Haasen, D.; Timmerman, M.; Sauerwein, R. W.; Suwanarusk, R.; Russell, B.; Renia, L.; Nosten, F.; Tully, D. C.; Kocken, C. H. M.; Glynne, R. J.; Bodenreider, C.; Fidock, D. A.; Diagana, T. T.; Winzeler, E. A. Targeting Plasmodium PI(4)K to Eliminate Malaria. *Nature* **2013**, *504* (7479), 248–253. https://doi.org/10.1038/nature12782.

(6) Cheuka, P. M.; Centani, L.; Arendse, L. B.; Fienberg, S.; Wambua, L.; Renga, S. S.; Dziwornu, G. A.; Kumar, M.; Lawrence, N.; Taylor, D.; Wittlin, S.; Coertzen, D.; Reader, J.; van der Watt, M.; Birkholtz, L.-M.; Chibale, K. New Amidated 3,6-Diphenylated Imidazopyridazines with Potent Antiplasmodium Activity Are Dual Inhibitors of *Plasmodium* Phosphatidylinositol-4-Kinase and CGMP-Dependent Protein Kinase. *ACS Infect Dis* **2021**, *7* (1), 34–46. https://doi.org/10.1021/acsinfecdis.0c00481.

(7) Arendse, L. B.; Murithi, J. M.; Qahash, T.; Pasaje, C. F. A.; Godoy, L. C.; Dey, S.; Gibhard, L.; Ghidelli-Disse, S.; Drewes, G.; Bantscheff, M.; Lafuente-Monasterio, M. J.; Fienberg, S.; Wambua, L.; Gachuhi, S.; Coertzen, D.; van der Watt, M.; Reader, J.; Aswat, A. S.; Erlank, E.; Venter, N.; Mittal, N.; Luth, M. R.; Ottilie, S.; Winzeler, E.

A.; Koekemoer, L. L.; Birkholtz, L.-M.; Niles, J. C.; Llinás, M.; Fidock, D. A.; Chibale, K. The Anticancer Human MTOR Inhibitor Sapanisertib Potently Inhibits Multiple *Plasmodium* Kinases and Life Cycle Stages. *Sci Transl Med* **2022**, *14* (667). https://doi.org/10.1126/scitranslmed.abo7219.

(8) Johnson, J. D.; Dennull, R. A.; Gerena, L.; Lopez-Sanchez, M.; Roncal, N. E.; Waters, N. C. Assessment and Continued Validation of the Malaria SYBR Green I-Based Fluorescence Assay for Use in Malaria Drug Screening. *Antimicrob Agents Chemother* **2007**, *51* (6), 1926–1933. https://doi.org/10.1128/AAC.01607-06.

(9) Hill, A. P.; Young, R. J. Getting Physical in Drug Discovery: A Contemporary Perspective on Solubility and Hydrophobicity. *Drug Discov Today* **2010**, *15* (15–16), 648–655. https://doi.org/10.1016/j.drudis.2010.05.016.

(10) Alelyunas, Y. W.; Pelosi-Kilby, L.; Turcotte, P.; Kary, M.-B.; Spreen, R. C. A High Throughput Dried DMSO LogD Lipophilicity Measurement Based on 96-Well Shake-Flask and Atmospheric Pressure Photoionization Mass Spectrometry Detection. *J Chromatogr A* **2010**, *1217* (12), 1950–1955. https://doi.org/10.1016/j.chroma.2010.01.071.

(11) Obach, R. S. Prediction of Human Clearance of Twenty-Nine Drugs from Hepatic Microsomal Intrinsic Clearance Data: An Examination of in Vitro Half-Life Approach and Nonspecific Binding to Microsomes. *Drug Metab Dispos* **1999**, *27* (11), 1350–1359.

(12) South African Bureau of Standards. *South African National Standard: The Care and Use of Animals for Scientific Purposes*, 1st ed.; SABS Standards Division, Pretoria, South Africa, 2008; Vol. SANS 10386:2008.

(13) Department of Health. *Ethics in Health Research: Principles, Processes and Structures*, 2nd ed.; Department of Health, Republic of South Africa, Pretoria, South Africa, 2015.

(14) Gibhard, L.; Njoroge, M.; Paquet, T.; Brunschwig, C.; Taylor, D.; Lawrence, N.; Abay, E.; Wittlin, S.; Wiesner, L.; Street, L. J.; Chibale, K.; Basarab, G. S. Investigating Sulfoxide-to-Sulfone Conversion as a Prodrug Strategy for a Phosphatidylinositol 4-Kinase Inhibitor in a Humanized Mouse Model of Malaria. *Antimicrob Agents Chemother* **2018**, *62* (12). https://doi.org/10.1128/AAC.00261-18.
